# Early-Stage Corticostriatal Hyperactivity Impairs Cognitive Flexibility Alongside Striatal Cholinergic Dysfunction in an Alzheimer’s Disease Model

**DOI:** 10.1038/s41467-026-74817-z

**Published:** 2026-06-26

**Authors:** Yufei Huang, Xueyi Xie, Zhenbo Huang, Ruifeng Chen, Himanshu Gangal, Xuehua Wang, Karienn A. de Souza, Julia Hunter, Xin Wu, Doodipala Samba Reddy, Jeannie Chin, Jun Wang

**Affiliations:** 1 Department of Neuroscience and Experimental Therapeutics, Naresh K. Vashisht College of Medicine, Texas A&M University Health Science Center, Bryan, TX USA; 2 Institute for Neuroscience, Texas A&M University, College Station, TX USA; 3 Interdisciplinary Faculty of Toxicology, College of Veterinary Medicine and Biomedical Sciences, Texas A&M University, College Station, TX USA; 4 Department of Neuroscience, Baylor College of Medicine, Houston, TX USA

**Keywords:** Neural circuits, Alzheimer's disease

## Abstract

Cognitive flexibility declines early in Alzheimer’s disease, yet the underlying circuit mechanisms remain unknown. Here, we report that young 5xFAD mice exhibit deficits in instrumental reversal learning prior to spatial memory impairment. This behavioral inflexibility is associated with abnormal neuronal reactivation in the medial prefrontal cortex and dorsomedial striatum. Electrophysiological recordings reveal that medial prefrontal cortex neurons are hyperexcitable and receive increased excitatory input. Furthermore, glutamatergic transmission from the medial prefrontal cortex to striatal direct-pathway medium spiny neurons is enhanced and coincides with strengthened inhibitory transmission onto striatal cholinergic interneurons, reduced spontaneous firing, and diminished striatal acetylcholine release. Critically, sustained chemogenetic inhibition of this corticostriatal circuit attenuates cortical amyloid accumulation, reduces glutamatergic transmission, and increases acetylcholine levels. This also rescues reversal learning deficits in 5xFAD mice. Here, we show that pathological corticostriatal hyperactivity contributes to early cognitive inflexibility in a mouse model of Alzheimer’s disease.

## Abstract (web summary)

Why flexible decision-making declines in early Alzheimer’s disease remains unclear. Here, the authors show that abnormal communication between the cortex and dorsal striatum impairs behavioral flexibility in a mouse model.

## Introduction

Alzheimer’s disease (AD) is the most common cause of dementia worldwide, affecting over 10% of individuals over 65 years old in the United States^1^. AD is characterized by the accumulation of amyloid-beta (Aβ) plaques and tau neurofibrillary tangles and by a progressive impairment of cognitive function^2–5^. Despite the development of FDA-approved Aβ-targeting antibodies and acetylcholinesterase (AChE) inhibitors, current treatments have limited effectiveness at later stages of the disease, when substantial neuronal loss has already occurred^6^. Therefore, identifying mechanisms driving early-stage pathophysiology is critical for developing therapeutic strategies capable of preserving cognitive function and modifying disease progression.

Although both Aβ and tau play critical roles in AD, there is evidence that the increase in Aβ occurs first and drives the preclinical and early stages of the disease^7–9^. Therefore, much effort has been devoted to understanding the mechanisms by which Aβ affects neuronal function early in AD. Several studies have demonstrated that Aβ accumulation disrupts glutamate reuptake, establishing a vicious cycle between Aβ deposition and neuronal hyperactivity^10–13^. Notably, Aβ accumulation initially emerges in cortical areas, including the medial prefrontal cortex (mPFC), and subsequently extends to innervated regions that include the hippocampus and striatum^14–16^. Although much attention has been paid to understanding how Aβ affects hippocampal function, less is known about how Aβ affects striatal circuits.

The dorsomedial striatum (DMS), a major target of mPFC projections, plays a critical role in goal-directed behavior and cognitive flexibility, which are core components of executive function that are compromised early in AD^17–25^. Cognitive flexibility refers to the ability of individuals to adapt their behaviors in response to changing circumstances or environmental cues^26^. Within the DMS, principal medium spiny neurons (MSNs) and cholinergic interneurons (CINs) interact to support this flexibility. CINs release acetylcholine (ACh), which regulates MSN activity to guide adaptive, goal-directed behaviors^27,28^. Although cholinergic dysfunction is a hallmark of AD, and the striatum is one of the brain regions with the highest AChE activity^29^, the impact of Aβ on striatal cholinergic signaling is not well characterized. In particular, it remains unclear whether cortical Aβ disrupts CIN function in the DMS and whether such disruption contributes to early deficits in cognitive flexibility. Addressing these questions could clarify the circuit-level basis of early executive dysfunction in AD and reveal potential therapeutic targets to restore cognitive function and alter disease trajectory.

In this work, we demonstrate that early cortical hyperactivity within the mPFC-to-DMS pathway contributes to reversal learning deficits using the 5xFAD mouse model of AD neuropathology^30^. Sustained chemogenetic inhibition of DMS-projecting mPFC neurons attenuates Aβ accumulation, normalizes corticostriatal excitatory transmission, restores cholinergic tone, and rescues cognitive flexibility. These findings reveal a circuit-level mechanism linking early Aβ pathology to cognitive inflexibility and suggest that targeting corticostriatal hyperactivity may offer a therapeutic entry point to slow disease progression in early-stage AD.

## Results

### Three-month-old 5xFAD mice show instrumental reversal learning deficits indicative of cognitive inflexibility

**Fig. 1:**
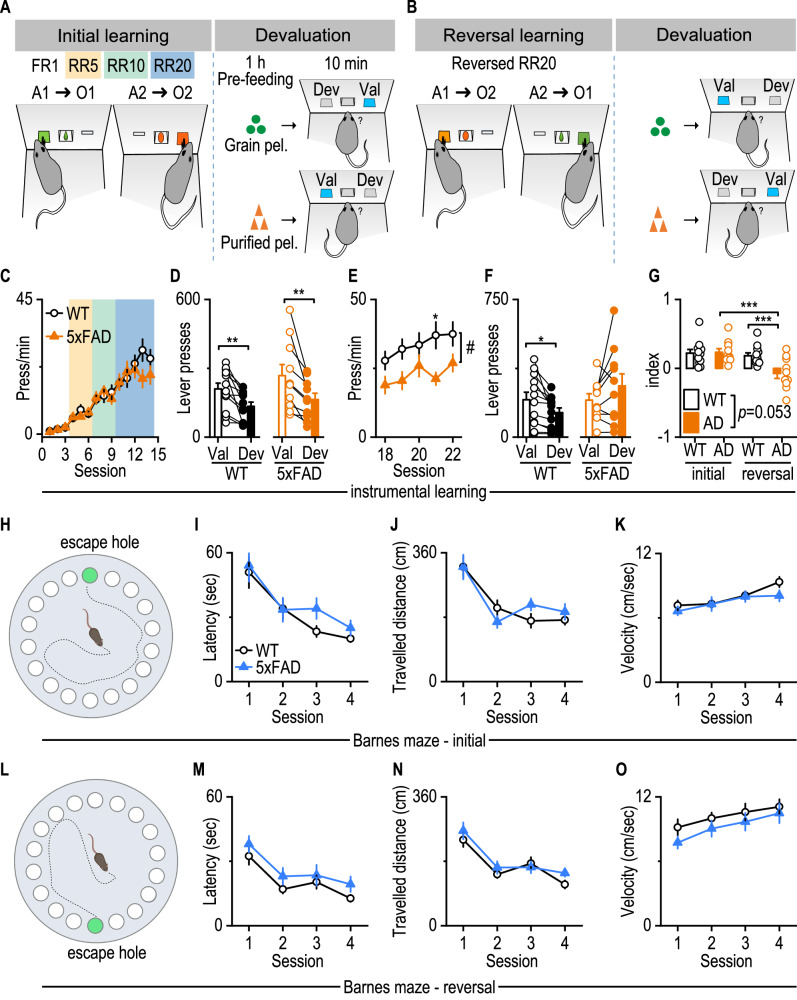
Early cognitive inflexibility emerges in 5xFAD mice during instrumental reversal learning. **A**, **B** Reversal instrumental learning paradigm. **C** Initial lever-press learning was similar in 3-month-old WT and 5xFAD mice. Two-way RM ANOVA. Genotype: F_(1,20)_ = 0.4, *p* = 0.534; session: F_(3.765,75.30)_ = 46.649, *p* < 0.001. **D** Both groups showed initial outcome devaluation. Paired t-test, t_11_ = 3.942, *p* = 0.0023 for WT; t_9_ = 3.628, *p* = 0.0055 for 5xFAD. *p *<* 0.05, **p *<* 0.01. **E** 5xFAD mice exhibited lower pressing rates than WT mice during reversal contingency training. Mixed-effects model. Genotype: F_(1,20)_ = 5.434, *p* = 0.0303. ^#^*p* *<* 0.05; post-hoc, ^*^p < 0.05. **F** WT, but not 5xFAD, mice showed reversal outcome devaluation. Paired t-test, t_11_ = 2.954, *p* = 0.0131 for WT; t_9_ = −*1.551*, *p* = 0.155 for 5xFAD. ^*^p *<* 0.05. **G** Reversal index, (Val–Dev)/(Val+Dev), was reduced in 5xFAD mice. Two-way RM ANOVA, genotype: F_(1,20)_ = 4.216, *p* = 0.053; interaction: F_(1,20)_ = 8.518, *p* = 0.0085; post-hoc, 5xFAD initial vs reversal: t = 4.49, *p* = 0.0002; 5xFAD reversal vs WT reversal: t = 3.41, *p* = 0.0015. ***p *<* 0.001. **H** Initial Barnes maze schematic. **I**–**K** Latency (**I**), distance (**J**) and velocity (**K**) did not differ by genotype during initial learning. Two-way RM ANOVA. Genotype: *F*_(1, 36)_ = 0.9137, *p* = 0.3455 (**I**); *F*_(1, 36)_ = 0.1402, *p* = 0.7103 (J); *F*_(1, 36)_ = 0.6623, *p* = 0.4211 (**K**). **L** Reversal Barnes maze schematic. **M**–**O** Latency (**M**), distance (**N**) and velocity (**O**) did not differ by genotype during reversal learning. Two-way RM ANOVA (**M**, **O**). GLMM (N). Genotype: *F*_(1, 36)_ = 2.184, *p* = 0.1482 (M); *F*_(1, 36)_ = 1.382, *p* = 0.2476 (N); *F*_(1, 36)_ = 1.117, *p* = 0.2975 (**O**). *n* = 12 (WT) and 10 (5xFAD) mice for panels (**C**–**G**); *n* = 22 (WT) and 16 (5xFAD) mice for panels (**I**–**O**). Data are mean ± SEM; tests were two-sided, with adjusted *P* values where applicable. Source data are provided as a Source Data file. Panels **H** and **L** were created with BioRender. Huang, Y. (https://BioRender.com/kh5jchl).

We examined reversal learning in young 5xFAD mice to assess cognitive flexibility at the early stages of Aβ deposition. In this task (Fig. 1A), 3-month-old mice were trained to associate the left lever (A1) with grain pellets (O1) and the right lever (A2) with purified pellets (O2), as established in prior studies^23,25,31–33^. These two pellet types are similarly palatable and preferred over standard chow, with no intrinsic bias. To verify contingency learning, we performed a devaluation test in which mice were pre-fed either O1 or O2 prior to a choice test. Animals that had successfully learned the action-outcome association pressed the lever associated with the devalued (pre-fed) outcome less. After confirming initial learning, we reversed the action-outcome contingencies, such that A1 now delivered O2 and A2 delivered O1 (Reversal RR20 schedule; Fig. 1B). Successful adaptation to this reversal is widely used as a behavioral measure of cognitive flexibility^34–36^.

During the initial training phase, 3-month-old wild-type (WT) and 5xFAD mice showed similar lever-pressing rates (Fig. 1C). Both groups increased their responses in subsequent sessions (Fig. 1C), indicating intact initial learning. To evaluate whether both groups learned the action-outcome contingency, we performed the devaluation test described above. We found that both WT and 5xFAD mice pressed less on devalued levers (associated with the pre-fed outcome) than on valued levers (associated with the non-pre-fed outcome). This demonstrated successful learning of the initial action-outcome contingencies (Fig. 1D).

During the reversal RR20 learning phase, 5xFAD mice showed significantly reduced lever-pressing rates, as compared to WT mice (Fig. 1E). Furthermore, while WT mice pressed the devalued lever less than the valued lever during the devaluation test, 5xFAD mice did not (Fig. 1F). These results suggested that 5xFAD mice exhibited a deficit in acquiring the new action-outcome contingencies. Quantification of the devaluation index further confirmed this impairment: 5xFAD mice showed a significantly lower reversal learning index, as compared to their own initial devaluation performance (Fig. 1G). Additionally, the reversal devaluation index in 5xFAD mice was marginally lower than in WT controls (Fig. 1G), suggesting impaired cognitive flexibility during instrumental reversal learning.

To determine whether these deficits generalized to other cognitive domains, we assessed spatial memory using the Barnes maze (Fig. 1H, L)^37,38^. At 3 months of age, 5xFAD and WT mice showed no significant differences in latency to locate the target escape chamber, distance traveled, or movement speed during both initial and reversal phases (Fig. 1I–O). To further assess reward-motivated spatial reversal learning^34^, we employed a radial arm maze task in which distinct subsets of arms were baited during the acquisition and reversal phases (Supplementary Fig. 1). No significant differences were observed between 5xFAD and WT mice in learning performance or reversal accuracy at 3–4 months of age, indicating intact reward-based spatial reversal learning in young 5xFAD mice. These results indicated that 5xFAD mice had no spatial learning or memory impairments at this age.

We next examined whether the reversal learning deficit persisted at later stages by testing 6-month-old 5xFAD and WT mice using the same instrumental reversal task. During initial training, both genotypes showed similar lever-pressing rates (Supplementary Fig. 2A), and both groups learned the initial action-outcome contingencies, although this was less robust in 5xFAD mice (Supplementary Fig. 2B). Lever-pressing rates remained comparable in the reversal learning phase (Supplementary Fig. 2C). However, while WT mice reduced their responses for the devalued outcome in the reversal devaluation test, 5xFAD mice did not (Supplementary Fig. 2D), suggesting continued impairment in learning new action-outcome associations in 5xFAD mice. Devaluation index analysis confirmed a significant impairment in reversal learning in 5xFAD mice, compared to both their initial learning and WT mice (Supplementary Fig. 2E). This indicated that cognitive flexibility deficits were still present in 6-month-old 5xFAD mice.

To assess spatial learning at 6 months of age, we repeated the Barnes maze test. In contrast to the 3-month-old cohort, 5xFAD mice at this age showed longer latencies and traveled greater distances to locate the escape hole during both the initial and reversal learning phases (Supplementary Fig. 2F-I), indicating spatial learning deficits. Importantly, spontaneous locomotor activity was similar in WT and 5xFAD mice of both sexes (Supplementary Fig. 3), excluding motor dysfunction as a confounding factor. To rule out motivational or reinforcer valuation confounds, we quantified satiety induction and reward motivation in WT and 5xFAD mice (Supplementary Fig. 4). WT and 5xFAD mice consumed similar amounts of food and showed comparable consumption rates during the one-hour prefeeding period (Supplementary Fig. 4E–F). Both genotypes exhibited intact outcome-specific satiety, consuming more valued than devalued pellets after prefeeding, with no difference in preference indices (Supplementary Fig. 4G–H). In addition, progressive ratio testing revealed no genotype differences in breakpoint or total responding for either pellet type (Supplementary Fig. 4I–N), indicating preserved motivation.

Together, these findings indicate that 5xFAD mice display cognitive inflexibility in instrumental reversal learning as early as 3 months of age, while spatial learning deficits emerge later, by 6 months of age.

### More mPFC and DMS neurons are reactivated during reversal learning in 5xFAD mice than in control animals

Given that 5xFAD mice exhibited intact initial learning and impaired reversal learning, we next examined whether this deficit was associated with an abnormal recruitment or reactivation of task-relevant neural ensembles. The mPFC and DMS represent key regions to investigate because they are critical for flexible, goal-directed behaviors^31–33,39–44^. To monitor neuronal activation during instrumental learning, we employed the ArcTRAP system^45–47^. This system uses the immediate early gene, *Arc*, to label recently activated neurons. Arc is rapidly induced by experience-dependent activity and is well-established as a marker of neural engagement in learning and memory^48^. We crossed ArcTRAP mice with Ai14 and 5xFAD lines to generate ArcTRAP;Ai14;5xFAD and control (ArcTRAP;Ai14) cohorts.

**Fig. 2:**
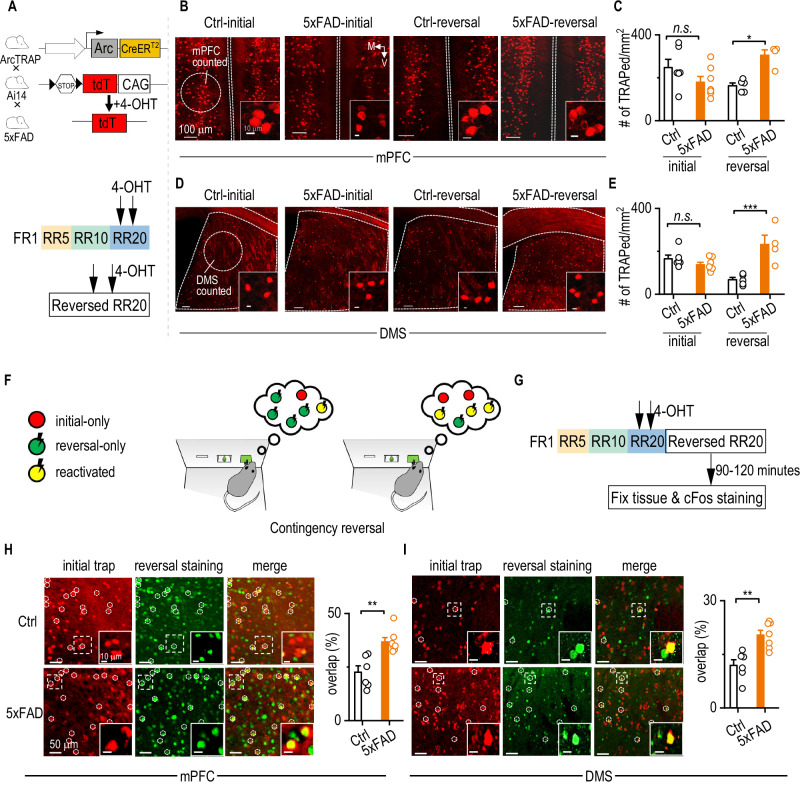
Reversal learning in 5xFAD mice reactivates more mPFC and DMS neurons. **A** Schematic diagrams illustrating the ArcTRAP and trapping strategy. **B** Representative images of tdTomato-labeled mPFC neurons. **C** 5xFAD mice exhibited a greater number of activated neurons in the mPFC during the reversal session compared with WT mice. Two-way ANOVA, interaction: F_(1, 18)_ = 12.40, *p* = 0.0024; post-hoc: reversal-WT vs reversal-5xFAD, *p* = 0.0372. *p < 0.05. *n* = 6 mice for Ctrl-initial; 7 mice for 5xFAD-initial; 5 mice for Ctrl-reversal and 4 mice for 5xFAD-reversal. **D** Representative images of tdTomato-labeled DMS neurons. **E** 5xFAD mice exhibited a greater number of activated neurons in the DMS during the reversal session compared with WT mice. Two-way ANOVA, interaction: *F*_(1, 18)_ = 21.63, *p* = 0.0002; post-hoc: reversal-WT vs. reversal-5xFAD: *p* = 0.0004. ***p < 0.001. *n* = 6 mice for Ctrl-initial; 7 mice for 5xFAD-initial; 5 mice for Ctrl-reversal and 4 mice for 5xFAD-reversal. **F**, **G** Schematic illustration of longitudinal tracking of neuronal activation during reversal learning. **H** Representative images of mPFC neurons co-labeled with tdTomato and c-Fos. 5xFAD mice showed greater overlap between initial- and reversal-learning activated mPFC neurons than control mice. Mann-Whitney Rank Sum Test, U = 0, *p* = 0.0012. ^**^p < 0.01. *n* = 6 mice for Ctrl; 7 mice for 5xFAD. **I** Representative images of DMS neurons co-labeled with tdTomato and c-Fos. 5xFAD mice showed greater DMS neuronal reactivation during reversal learning than control mice. Unpaired t-test, t_11_ = 4.042, *p* = 0.0019. ^**^p < 0.01. *n* = 6 mice for Ctrl; 7 mice for 5xFAD. Each data point represents one biologically independent mouse; values from multiple tissue sections per mouse were averaged before statistical analysis (panels **C**, **E**, **H**, and **I**). Data are mean ± SEM; tests were two-sided, with adjusted *P* values where applicable. Source data are provided as a Source Data file.

To capture learning-related neuronal activation, 4-hydroxytamoxifen (4-OHT) was administered immediately after the second and third RR20 training sessions during both initial and reversal learning phases, triggering tdTomato expression in Arc-expressing neurons (Fig. 2A). We then quantified tdTomato-labeled neurons in the mPFC, DMS, and dentate gyrus of the hippocampus. During initial learning, ArcTRAP;Ai14 controls and ArcTRAP;Ai14;5xFAD mice showed comparable densities of activated neurons in the mPFC and DMS (Fig. 2B-E). However, during reversal learning, ArcTRAP;Ai14;5xFAD mice displayed significantly greater neuronal activation than ArcTRAP;Ai14 control mice. This difference was significant in both the mPFC (Fig. 2C) and DMS (Fig. 2E). In contrast, there was no significant difference in the number of activated neurons in the hippocampal dentate gyrus between learning phases in either ArcTRAP;Ai14 or ArcTRAP;Ai14;5xFAD mice (Supplementary Fig. 5). These findings suggest that the mPFC and DMS, but not the hippocampus, are more active in 5xFAD animals than in controls during reversal learning.

To determine whether this heightened activation reflected aberrant re-engagement of the same neuronal ensembles involved in initial learning, we performed a longitudinal tagging experiment. Neurons activated during initial learning were labeled using tdTomato (via ArcTRAP;Ai14), while those recruited during reversal learning were identified using c-Fos immunostaining (Fig. 2F, G). This approach allowed us to quantify the proportion of initial learning-activated neurons (tdTomato + ) that were reactivated during reversal learning (tdTomato+ & c-Fos + ) within the same animal. We found that ArcTRAP;Ai14;5xFAD mice exhibited significantly greater co-expression of tdTomato and c-Fos in mPFC neurons (Fig. 2H) and DMS neurons (Fig. 2I), as compared to ArcTRAP;Ai14 mice. Together, these results suggest that more mPFC and DMS neurons are reactivated during reversal learning in 5xFAD mice than in control animals.

### mPFC neurons in young 5xFAD mice are hyperexcitable and receive increased excitatory input

To examine whether functional abnormalities in mPFC neurons are also evident at the cellular and synaptic level, we next performed whole-cell recordings. We focused on 4-month-old 5xFAD mice because early-stage cortical amyloid pathology^14,49^ and neuronal hyperactivity^12,50^ have been reported at this age.

**Fig. 3:**
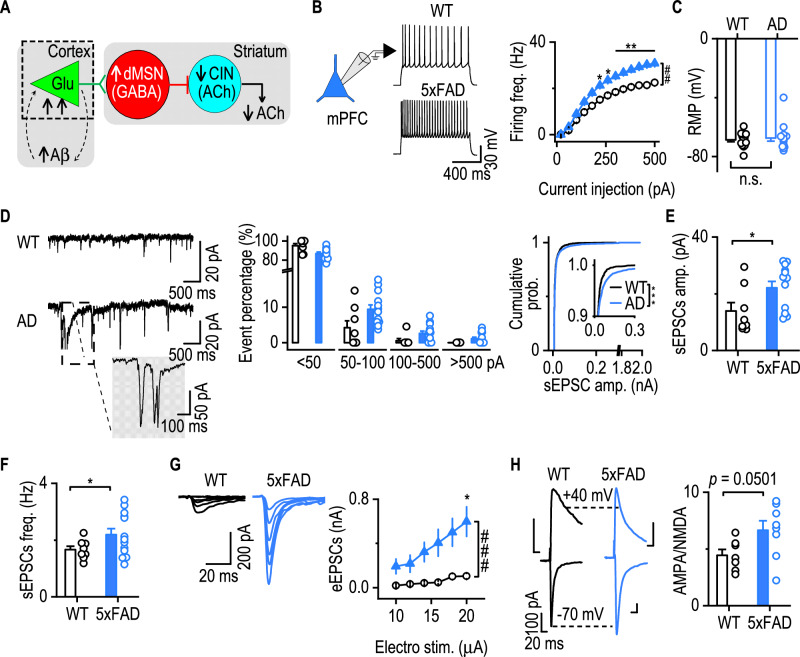
mPFC neurons in young 5xFAD mice are hyperexcitable and receive increased excitatory input. mPFC slices were prepared from 4-month-old WT and 5xFAD mice. mPFC neurons were patched from the prelimbic cortex, layer 5. **A** Proposed circuit mechanism; dashed area indicates the mPFC recording focus. **B** mPFC neurons from 5xFAD mice showed increased evoked firing. Two-way RM ANOVA, genotype: *F*_(1,29)_ = 13.10, *p* = 0.0011. ^##^*p* < 0.01; post-hoc analysis, *p < 0.05, **p < 0.01 at matched current steps. Current injection: 300 pA, 1 sec. *n* = 15 neurons from 3 mice (WT) and 16 neurons from 5 mice (5xFAD). **C** Resting membrane potential did not differ by genotype. Mann–Whitney test. U = 112.0, *p* = 0.7625. *n* = 15 neurons from 3 mice (WT) and 16 neurons from 5 mice (5xFAD). **D** Representative sEPSC traces and amplitude distributions showed increased sEPSC amplitudes in 5xFAD mice. Kolmogorov-Smirnov Test, D = 0.05046, *p* = 0.000297. ^***^p < 0.001. *n* = 2520 events from 3 WT; 5414 events from 3 5xFAD mice. **E**, **F** sEPSC amplitude (**E**) and frequency (**F**) were increased in 5xFAD mice. Mann-Whitney Test, *U* = 20, *p* = 0.0199 (amplitude); Welch’s unpaired t-test, *t*_17.80_ = 2.196, *p* = 0.0416 (frequency); *p < 0.05. *n* = 8 neurons from 3 mice (WT) and 13 neurons from 3 mice (5xFAD). **G** Evoked EPSCs were larger in 5xFAD mice. Two-way RM ANOVA, interaction: *F*_(1.590, 30.21)_ = 15.09, *p* < 0.0001; genotype: *F*_(1,19)_ = 15.881, *p* = 0.0008. ^###^*p* < 0.001; post-hoc, ^*^p < 0.05, **p < 0.01, ***p < 0.001 at matched intensities. *n* = 13 neurons from 3 mice (WT) and 8 neurons from 3 mice (5xFAD). **H** Sample trace of AMPA/NMDA ratio. AMPA/NMDA ratio was higher in 5xFAD mice. Unpaired t-test, *t*_13_ = −2.160, *p* = 0.0501. *n* = 7 neurons from 3 mice (WT) and 8 neurons from 3 mice (5xFAD). Data are mean ± SEM; tests were two-sided, with adjusted *P* values where applicable. Source data are provided as a Source Data file.

We performed whole-cell patch-clamp recordings from pyramidal neurons in layer 5 in the mPFC, mainly prelimbic cortex, to assess their intrinsic excitability (Fig. 3A). Compared to age-matched WT controls, mPFC neurons from 5xFAD mice exhibited significantly higher firing rates in response to increasing current injections (Fig. 3B), indicating greater intrinsic excitability. The resting membrane potential remains similar between groups (Fig. 3C). To examine synaptic input, we recorded spontaneous excitatory postsynaptic currents (EPSCs) (Fig. 3D). We sampled the event distribution based on sEPSC amplitude and observed that most EPSCs in both groups were small ( < 50 pA), whereas large-amplitude events, including rare events exceeding 500 pA, were observed in recordings from mPFC neurons of 5xFAD mice (Fig. 3D). Both the amplitude (Fig. 3E) and frequency (Fig. 3F) of spontaneous EPSCs were significantly elevated in 5xFAD mice, suggesting enhanced baseline glutamatergic transmission.

We next evaluated evoked synaptic responses to assess functional synaptic drive. Electrically evoked EPSCs were significantly larger in 5xFAD mice than in WT controls (Fig. 3G). The AMPA/NMDA ratio was also elevated in 5xFAD mice (Fig. 3H), indicating a greater contribution of AMPA receptor-mediated currents.

Together, these findings demonstrate that mPFC neurons in young 5xFAD mice are intrinsically hyperexcitable and receive augmented excitatory synaptic input.

### The mPFC-to-dMSN circuit and dMSNs are hyperactive in 5xFAD mice

**Fig. 4:**
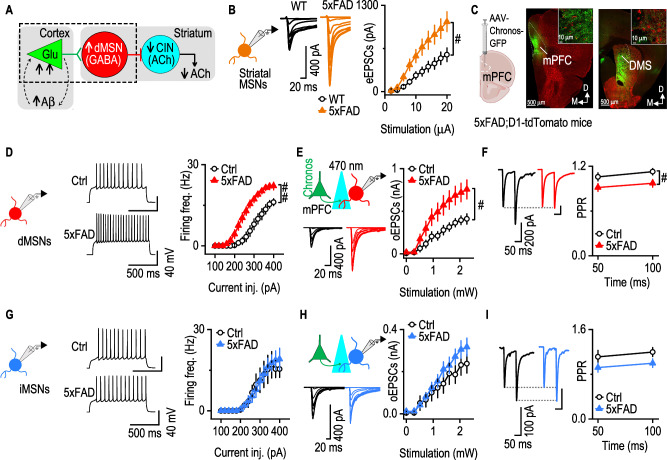
The mPFC-to-dMSN circuit and dMSNs are hyperactive in 5xFAD mice. **A** Proposed neurocircuit mechanism; dashed area indicates the mPFC-to-dMSN projection tested. **B** Evoked EPSC amplitudes in DMS MSNs were larger in 4-month-old 5xFAD mice than WT controls. Two-way RM ANOVA, genotype: *F*_(1,40)_ = 6.836, *p* = 0.013, ^#^*p* < 0.05. *n* = 22 neurons from 5 mice (WT); 20 neurons from 5 mice (5xFAD). **C** Representative image showing Chronos-eGFP expression in mPFC and projections to DMS in 5xFAD;D1-tdTomato mice. **D** Diagram illustrating recording of dMSNs. dMSN recordings showed increased evoked firing in 5xFAD mice. GLMM, genotype: *F*_(1, 14)_ = 11.03, *p* = 0.0050, ^##^*p* < 0.01. *n* = 8 neurons from 4 mice (Ctrl and 5xFAD) **E** oEPSC amplitude in DMS dMSNs was greater in 4-month-old 5xFAD mice than in controls. Two-way RM ANOVA, interaction: *F*_(1.639, 37.70)_ = 4.298, *p* = 0.0272; genotype: *F*_(1, 23)_ = 4.694, *p* = 0.0409, ^#^*p* < 0.05. *n* = 10 neurons from 4 mice (WT); 15 neurons from 4 mice (5xFAD). **F** PPR was smaller in 5xFAD mice than in controls. Two-way RM ANOVA, genotype: *F*_(1, 16)_ = 5.280, *p* = 0.0354, ^#^*p* < 0.05. *n* = 8 neurons from 4 mice (WT); 10 neurons from 4 mice (5xFAD). **G** Diagram illustrating recording of iMSNs. iMSNs in 5xFAD mice show similar evoked firing frequency to that in WT mice. GLMM, genotype: *F*_(1, 14)_ = 0.2383, *p* = 0.6330. *n* = 7 neurons from 4 mice (Ctrl); 9 neurons from 4 mice (5xFAD). **H** oEPSC amplitude in DMS iMSNs was similar in 5xFAD mice to that in controls. Two-way RM ANOVA, genotype: *F*_(1, 18)_ = 0.8385, *p* = 0.3719. *n* = 10 neurons from 4 mice (Ctrl and 5xFAD). **I** PPR of oEPSCs in iMSNs did not differ by genotype. Two-way RM ANOVA, genotype: *F*_(1, 12)_ = 2.715, *p* = 0.1253. *n* = 7 neurons from 4 mice (Ctrl and 5xFAD). Data are mean ± SEM; tests were two-sided, with adjusted *P* values where applicable. Source data are provided as a Source Data file.

Given the hyperexcitability of mPFC neurons observed in 4-month-old 5xFAD mice, we next investigated whether this elevated cortical activity altered downstream striatal function, particularly within the mPFC-to-DMS circuit. The mPFC projects directly to the DMS^51,52^, a critical region for flexible behavior and reversal learning^21,33,53^. We first examined excitatory synaptic transmission onto striatal MSNs, the principal output neurons of the DMS (Fig. 4A). Electrically evoked EPSCs were significantly larger in 5xFAD mice than in WT controls (Fig. 4B), indicating enhanced excitatory transmission.

Since MSNs are subdivided into dMSNs and indirect-pathway MSNs (iMSNs), we next asked whether altered mPFC inputs affect one or both MSN subpopulations in 4-month-old 5xFAD mice. To selectively activate mPFC projections, we infused AAV-Chronos-GFP into the mPFC of D1-tdTomato;5xFAD and D1-tdTomato (control) mice, enabling optogenetic stimulation of mPFC terminals in the DMS while distinguishing dMSNs (tdTomato-positive) from iMSNs (tdTomato-negative) (Fig. 4C). Viral expression was verified during electrophysiological recordings by confirming GFP expression at the mPFC injection site and the presence of labeled mPFC projections in the DMS. Slices were excluded when GFP expression at the injection site was absent or when labeled DMS projections were not observed. In 4-month-old D1-tdTomato;5xFAD mice, dMSNs exhibited significantly greater excitability in response to current injections than they did in D1-tdTomato controls (Fig. 4D). Moreover, optogenetically evoked EPSCs in dMSNs were also significantly larger in D1-tdTomato;5xFAD mice (Fig. 4E), and the paired-pulse ratio (PPR) of the EPSCs was reduced (Fig. 4F), indicating increased presynaptic glutamate release from mPFC terminals. Notably, this hyperactivity persisted in 12-month-old D1-tdTomato;5xFAD mice, suggesting a sustained and progressive synaptic alteration (Supplementary Fig. 6A and B).

In contrast, the mPFC-to-iMSN pathway appeared unaffected. Comparison of 4-month-old D1-tdTomato;5xFAD and D1-tdTomato control mice revealed no significant differences in iMSN excitability (Fig. 4G), EPSC amplitude (Fig. 4H), or PPR (Fig. 4I). In addition, no differences in these parameters were observed at 12 months of age (Supplementary Fig. 6C and D). These ex vivo findings indicated that the observed hyperactivity was associated with the mPFC-to-dMSN circuit rather than the mPFC-to-iMSN pathway.

Separately, to determine whether 5xFAD mice also exhibit broader network hyperexcitability in vivo, we examined EEG activity before and after pilocarpine injection. Using 6 Hz corneal stimulation (Supplementary Fig. 7A), we found that 5xFAD mice had a lower threshold for seizure induction (Supplementary Fig. 7B) and displayed significantly longer seizures of greater severity, as compared to WT mice (Supplementary Fig. 7C, D). We further confirmed this in vivo hyperactivity using EEG recordings before and after pilocarpine injection (Supplementary Fig. 7E). Following pilocarpine administration, 5xFAD mice exhibited significantly elevated spike rates relative to WT controls (Supplementary Fig. 7F, G). Survival analysis also showed a clear difference between groups because 5xFAD mice failed to survive pilocarpine injection (Supplementary Fig. 7H), indicating increased seizure vulnerability.

Together, our ex vivo recordings indicate enhanced excitatory drive onto dMSNs within the mPFC–DMS circuit, whereas the seizure and EEG assays provide independent evidence of broader network-level hyperexcitability in 5xFAD mice and were not intended as pathway-specific confirmation.

### Enhanced dMSN-to-CIN inhibition coincides with reduced CIN activity and striatal ACh release in 5xFAD mice

**Fig. 5:**
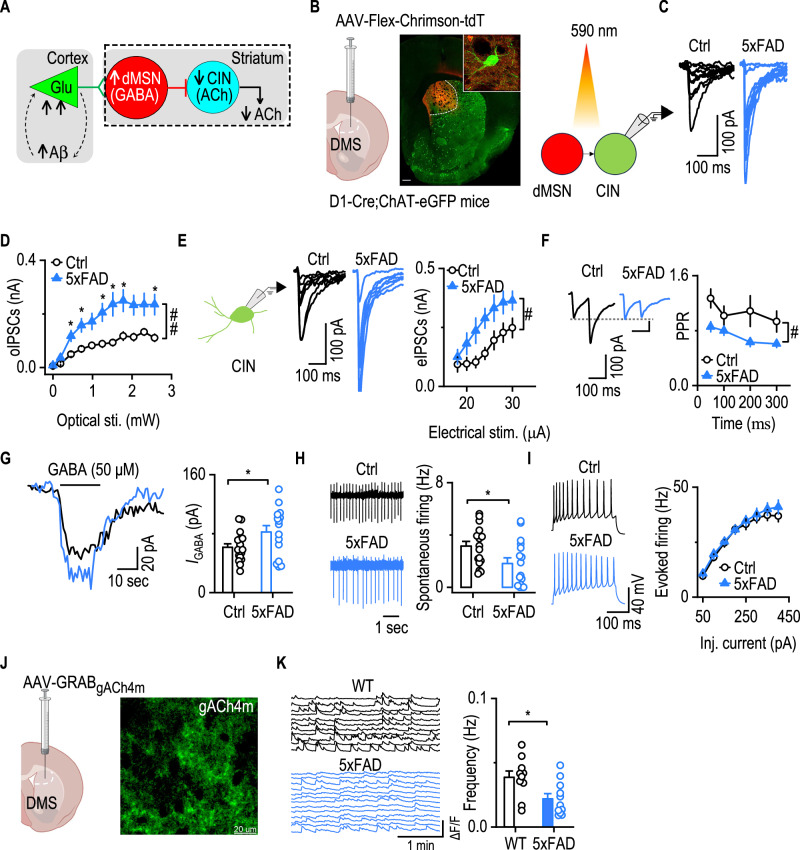
Enhanced dMSN-to-CIN inhibition coincides with reduced CIN activity and striatal ACh release in 5xFAD mice. DMS-CINs were identified by GFP in 4- to 5-month-old 5xFAD; ChAT-eGFP and ChAT-eGFP control slices. **A** Proposed circuit mechanism; dashed area highlights CIN and dMSN-to-CIN transmission. **B** Virus expression; CINs were recorded in tdTomato-positive fiber-rich area. **C** Representative oIPSC traces. **D** CINs in 5xFAD mice received stronger oIPSCs from dMSNs than controls. Two-way RM ANOVA, interaction: *F*_(2.309, 103.9)_ = 5.519, *p* = 0.0035; genotype: *F*_(1, 45)_ = 10.02, *p* = 0.0028. ^##^*p* < 0.01; post-hoc, *p < 0.05 at matched stimulation. *n* = 23 (Ctrl) and 24 (5xFAD) neurons from 3 mice/group. **E** CINs in 5xFAD mice displayed stronger eIPSCs than in Ctrl mice. Two-way RM ANOVA, genotype: *F*_(1, 27)_ = 5.502, *p* = 0.0266. ^#^*p* < 0.05. *n* = 14 (Ctrl) and 15 (5xFAD) neurons from 4 mice/group. **F** PPR was smaller in 5xFAD mice than in controls. Two-way RM ANOVA, genotype: *F*_(1, 27)_ = 6.545, *p* = 0.0164. ^#^*p* < 0.05. *n* = 14 (Ctrl) and 15 (5xFAD) neurons from 4 mice/group. **G** CINs in 5xFAD mice showed higher GABA (50 µM)-induced currents. Welch’s unpaired t-test, *t*_21.57_ = 2.155, *p* = 0.0426. *p < 0.05. *n* = 18 (Ctrl) and 15 (5xFAD) neurons from 4 mice/group. **H** CINs in 5xFAD mice displayed reduced spontaneous firing rates. Mann-Whitney Rank Sum Test, U = 72, *p* = 0.022. *p < 0.05. *n* = 17 (Ctrl) and 16 (5xFAD) neurons from 4 mice/group. **I** Evoked firing of CINs was similar between the two groups. Two-way RM ANOVA, genotype: *F*_(1,29)_ = 0.434, *p* = 0.515. *n* = 17 neurons from 4 mice (Ctrl); 14 neurons from 5 mice (5xFAD). **J** Confocal image of acetylcholine sensor. **K** Representative spontaneous ACh release traces; event frequency was reduced in 5xFAD mice. Unpaired t-test, *t*_19_ = 2.656, *p* = 0.0156. *p < 0.05. *n* = 10 (WT) and 11 (5xFAD) slices from 4 mice/group. Data are mean ± SEM; tests were two-sided, with adjusted *P* values where applicable. Source data are provided as a Source Data file. Panels **B** and **J** were created in BioRender. Huang, Y. https://BioRender.com/kh5jchl.

Having demonstrated that dMSNs were hyperactive in 5xFAD mice and given prior reports that dMSNs directly inhibited CINs in the striatum^25,54^, we hypothesized that elevated dMSN activity would disrupt CIN function (Fig. 5A). To test this hypothesis, we optogenetically activated dMSNs while recording from CINs in 4- to 5-month-old D1-Cre;ChAT-eGFP;5xFAD and D1-Cre;ChAT-eGFP (control) mice (Fig. 5B). AAV-FLEX-Chrimson-tdTomato was infused into the DMS to selectively express Chrimson in dMSNs, and 590-nm light was used to activate these projections. CINs in D1-Cre;ChAT-eGFP;5xFAD mice exhibited significantly larger optically evoked inhibitory postsynaptic currents (IPSCs), as compared to D1-Cre;ChAT-eGFP controls (Fig. 5C-D). This indicated that dMSN-to-CIN inhibition was significantly enhanced in this mouse model of AD.

Next, we investigated whether the enhanced dMSN-to-CIN inhibition observed with optogenetic activation could also be detected using electrical stimulation, which recruits all synaptic inputs, including those from dMSNs. To do so, we performed whole-cell recordings of IPSCs evoked by electrical stimulation. CINs from 5xFAD mice exhibited significantly larger IPSC amplitudes than WT mice (Fig. 5E). We then explored the mechanism underlying this increased CIN inhibition by evaluating presynaptic and postsynaptic components. PPR values were significantly reduced in D1-Cre;ChAT-eGFP;5xFAD mice (Fig. 5F), indicating increased presynaptic GABA release from inhibitory inputs onto CINs. To assess postsynaptic changes, we bath-applied GABA (50 µM) and measured the induced current in CINs. GABA-evoked responses were significantly greater in D1-Cre;ChAT-eGFP;5xFAD mice than in D1-Cre;ChAT-eGFP control mice (Fig. 5G), indicating increased postsynaptic GABA_A_ receptor responsiveness. We then assessed the functional impact of this increased inhibitory tone on CIN activity. Spontaneous firing of CINs was significantly reduced in D1-Cre;ChAT-eGFP;5xFAD mice, as compared to D1-Cre;ChAT-eGFP controls (Fig. 5H), whereas evoked firing responses remained intact (Fig. 5I). These results are consistent with CIN hypoactivity arising from increased inhibitory input rather than changes in intrinsic excitability.

To assess whether reduced CIN activity translated to decreased cholinergic output, we expressed the genetically encoded ACh sensor, GRAB_gACh4m_^55^, in the DMS (Fig. 5J) of 5xFAD and WT control mice. We observed a marked reduction in the frequency of spontaneous ACh release events in 5xFAD mice, as compared to WT controls (Fig. 5K), indicating diminished cholinergic tone. Given that the ACh released by CINs modulates MSNs indirectly via GABAergic interneurons^27^, we next examined whether CIN-to-MSN inhibition was also altered. To address this, we generated ChAT-Cre;Ai32;5xFAD and ChAT-Cre;Ai32 (control) mice, and optogenetically stimulated CINs while recording IPSCs in MSNs. We found that CIN-to-MSN IPSCs were significantly lower in ChAT-Cre;Ai32;5xFAD mice than in ChAT-Cre;Ai32 controls (Supplementary Fig. 8).

Together, these findings indicate coordinated disruption of dMSN–CIN interactions and cholinergic signaling in 5xFAD mice, consistent with altered striatal circuit function associated with increased mPFC-to-DMS drive.

### Sustained mPFC-to-DMS inhibition slows Aβ accumulation, reduces glutamatergic transmission, and enhances striatal ACh in 5xFAD mice

Given that mPFC hyperactivity emerges early in 5xFAD mice alongside cortical Aβ accumulation (Fig. 3) and that the mPFC-to-DMS circuit shows excessive output (Fig. 4) and is associated with downstream cholinergic alterations (Fig. 5), we next asked whether sustained inhibition of this pathway affected these pathophysiological changes. Prior studies have suggested the existence of a feed-forward loop in which neuronal hyperactivity promotes Aβ accumulation, which in turn impairs glutamate clearance and further increases neuronal excitability^13,56,57^. Also, we found little to no detectable Aβ accumulation in the striatum at early stages of AD (Supplementary Fig. 9). We, therefore, tested whether chemogenetic inhibition of the mPFC-to-DMS projection could reduce cortical Aβ accumulation, normalize glutamatergic output, and restore striatal cholinergic tone.

**Fig. 6:**
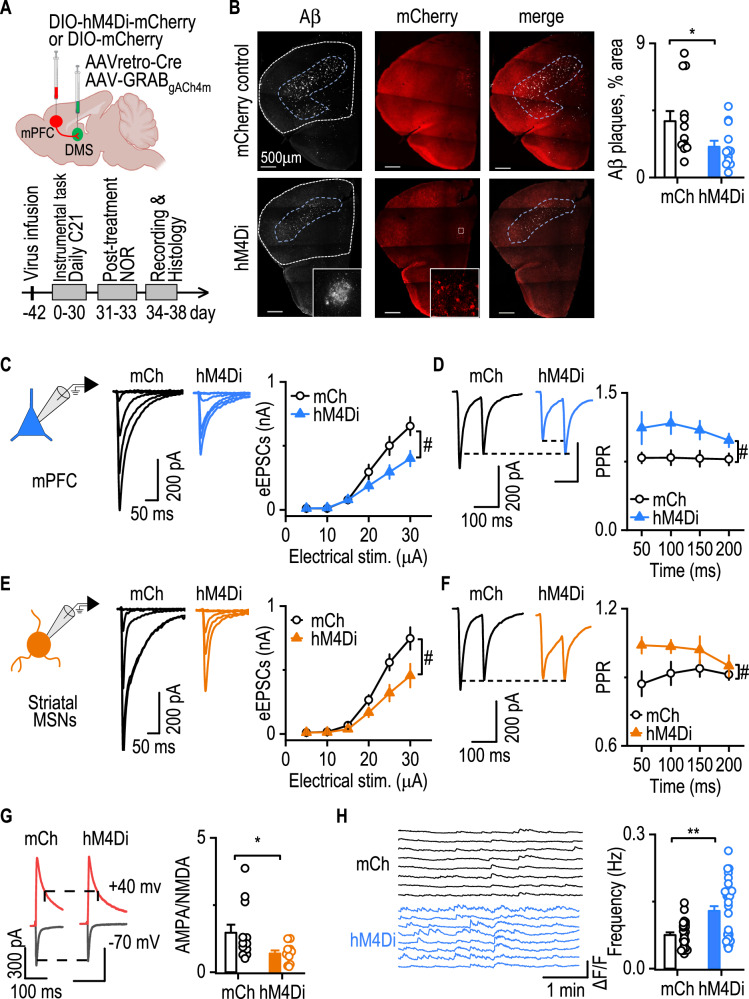
Sustained mPFC-to-DMS inhibition slows Aβ accumulation, reduces glutamatergic transmission, and enhances striatal ACh in 5xFAD mice. **A** Viral infusion strategy and experimental timeline. **B** Representative Aβ immunostaining images. White dashed outline marks the region of interest; blue dashed outline indicates the Aβ-positive area. hM4Di-injected 5xFAD mice showed reduced Aβ plaque coverage versus mCherry controls. Mann-Whitney Rank Sum Test, *U* = 37, *p* = 0.0257. *p < 0.05. *n* = 13 mice (mCh) and 12 mice (hM4Di). **C** eEPSC amplitudes in mPFC-mCherry positive neurons were lower in the hM4Di group than in mCherry controls. Two-way RM ANOVA, group: *F*_(1, 28)_ = 4.621, *p* = 0.0404. ^#^*p* < 0.05. *n* = 17 (mCherry) and 13 (hM4Di) neurons from 4 mice/group. **D** PPRs in mPFC-mCherry positive neurons were higher in hM4Di-injected mice than mCherry controls. Two-way RM ANOVA, group: *F*_(1, 25)_ = 4.782, *p* = 0.0383. ^#^*p* < 0.05. *n* = 15 (mCherry) and 12 (hM4Di) neurons from 4 mice/group. **E** eEPSC amplitudes in DMS MSNs were smaller in hM4Di-injected 5xFAD mice than in mCherry controls. Two-way RM ANOVA, group: *F*_(1, 25)_ = 6.343, *p* = 0.0186. ^#^*p* < 0.05. *n* = 14 (mCherry) and 13 (hM4Di) neurons from 4 mice/group. **F** PPR in DMS MSNs was higher in hM4Di- than in mCherry-injected 5xFAD mice. Two-way RM ANOVA, group: *F*_(1, 16)_ = 4.695, *p* = 0.0457. ^#^*p* < 0.05. *n* = 10 (mCherry) and 8 (hM4Di) neurons from 4 mice/group. **G** AMPA/NMDA ratio was lower in hM4Di- than in mCherry-injected 5xFAD mice. Mann-Whitney Rank Sum Test, *U* = 31, *p* = 0.0317. ^*^p < 0.05. *n* = 12 (mCherry) and 11 (hM4Di) neurons from 4 mice/group. **H** Sample traces of spontaneous striatal ACh release. The spontaneous ACh release frequency was higher in hM4Di-injected mice than in mCherry-injected 5xFAD mice. Mann-Whitney Test, *U* = 221, *p* = 0.0014. **p < 0.01. *n* = 25 slices from 13 mice (mCh); 34 slices from 12 mice (hM4Di). Data are mean ± SEM; two-sided tests with adjusted *P* values where applicable. Source data are provided as a Source Data file. Panel A was created in BioRender. Huang, Y. (https://BioRender.com/kh5jchl).

**Fig. 7:**
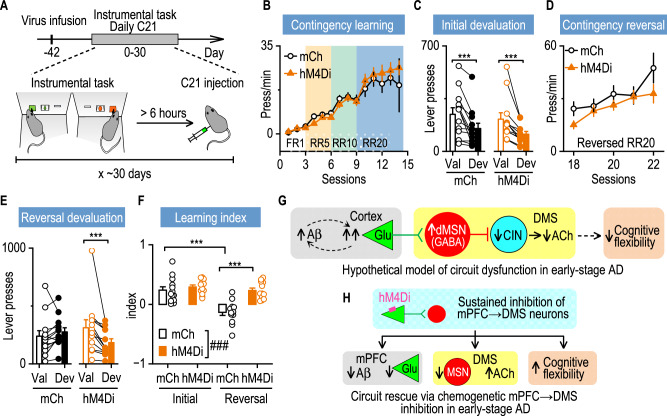
Sustained mPFC-to-DMS inhibition rescues reversal learning deficits in 5xFAD mice. **A** Expanded view of instrumental training and daily C21 treatment shown in Fig. 6A; C21 was administered ≥6 h after each session for ~30 days. **B** hM4Di- and mCherry-injected 5xFAD mice exhibited similar lever-press rates during initial contingency learning. Two-way RM ANOVA, group: F_(1, 23)_ = 0.182, *p* = 0.675. **C** Both groups showed initial outcome devaluation. Wilcoxon Signed Rank Test, W = −*91*, *p* = 0.0002 for mCherry; W = −*78*, *p* = 0.0005 for hM4Di. ^***^p *<* 0.001. n = 13 (mCh) and 12 (hM4Di). **D** During reversal contingency training, mCherry- (mCh) and hM4Di-injected (hM4Di) mice exhibited similar pressing rates. Two-way RM ANOVA, group: F_(1,23)_ = 1.836, *p* = 0.189. **E** hM4Di-injected (hM4Di) mice, but not mCherry- (mCh) 5xFAD, mice displayed significantly fewer lever presses for devalued than valued outcomes (Val) during the reversal devaluation test. Wilcoxon Signed Rank Test, W = 55, *p* = 0.057 for mCherry; W = −*78*, *p* = 0.0005 for hM4Di. ^***^p < 0.001. **F**, hM4Di-injected (hM4Di) mice exhibited a significantly higher reversal index than the reversal devaluation index of mCherry-injected (mCh) mice. The index is calculated as (Val-Dev)/(Val + Dev). Two-way RM ANOVA, group: F_(1,23)_ = 37.974, *p* < 0.001. ^###^*p* < 0.001; post-hoc, mCh-initial vs mCh-reversal: t = 4.529, *p* = 0.0003; hM4Di-reversal vs mCh-reversal: t = 5.352, *p* < 0.0001 ***p *<* 0.001. **G**, **H** Schematic models of corticostriatal alterations and intervention effects in early-stage AD. Increased corticostriatal activity in early-stage AD is associated with elevated dMSN activity, reduced CIN/ACh signaling, and concurrent impairment in cognitive flexibility (**G**). Chemogenetic inhibition of DMS-projecting mPFC neurons reduces cortical Aβ, normalizes corticostriatal transmission, and increases striatal cholinergic signaling, with concurrent improvement in cognitive flexibility in 5xFAD mice. (**H**) *n* = 13 mCherry- and 12 hM4Di-injected mice for panels B-F. Data are mean ± SEM; two-sided tests with adjusted *P* values where applicable. Source data are provided as a Source Data file.

As a first step, we confirmed that acute chemogenetic inhibition of mPFC neurons reduced both intrinsic excitability and synaptic transmission in ex vivo slices (Supplementary Fig. 10). However, previous work indicated that sustained neuronal silencing over several weeks was necessary to impact Aβ pathology^58^. To achieve long-term, projection-defined inhibition of mPFC-to-DMS neurons, we injected AAVretro-Cre into the DMS and Cre-dependent AAV-DIO-hM4Di-mCherry (or AAV-DIO-mCherry as a control) into the mPFC (PL/IL) of ~2-month-old 5xFAD mice. This dual-virus strategy restricted hM4Di expression to mPFC neurons projecting to the DMS via retrograde Cre delivery (Fig. 6A and Supplementary Fig. 11). We also co-injected AAV-GRAB_gACh4m_ into the DMS to monitor striatal ACh dynamics. After allowing ~42 days for viral expression, mice underwent a 30-day instrumental task together with daily C21 treatment. After completion of the treatment period, C21 administration was discontinued, and mice underwent post-treatment novel object recognition (NOR), electrophysiological recordings, and histological analyses within one week of the final injection (Fig. 6A; for instrumental task results, see Fig. 7).

Cortical Aβ plaque analysis revealed a significantly lower plaque-covered area in hM4Di-injected mice, as compared to mCherry controls (Fig. 6B). This indicated that sustained inhibition of the mPFC-to-DMS circuit slowed Aβ accumulation. Electrophysiological recordings showed reduced excitatory drive in both the mPFC and DMS. Specifically, evoked EPSCs were smaller in mPFC neurons from hM4Di-injected mice (Fig. 6C), and PPRs were elevated (Fig. 6D), suggesting reduced presynaptic glutamate release in the mPFC. Similarly, DMS MSNs in hM4Di-injected mice exhibited lower evoked EPSC amplitudes (Fig. 6E), increased PPRs (Fig. 6F), and a lower AMPA/NMDA ratio (Fig. 6G). This indicated reduced excitatory transmission in the striatum.

We next monitored spontaneous ACh release in the DMS using the GRAB_gACh4m_ sensor following sustained inhibition of mPFC neurons. Selective inhibition of DMS-projecting mPFC neurons via hM4Di significantly increased ACh event frequency compared to controls (Fig. 6H).

To determine whether these effects extended beyond projection-specific inhibition to more widespread cortical suppression, we performed a parallel experiment using global mPFC inhibition by broadly expressing hM4Di in mPFC neurons (Supplementary Fig. 12A). Global mPFC inhibition similarly reduced cortical Aβ accumulation (Supplementary Fig. 12B, C), suppressed glutamatergic transmission in both the mPFC (Supplementary Fig. 12D–J) and striatum (Supplementary Fig. 13A–D), and increased striatal ACh release (Supplementary Fig. 13E–L).

Taken together, sustained inhibition of the mPFC-to-DMS circuit led to concurrent reductions in cortical Aβ accumulation, normalization of glutamatergic transmission, and increases in striatal ACh release.

### Sustained mPFC-to-DMS inhibition rescues reversal learning deficits in 5xFAD mice

Having shown that the mPFC-to-DMS circuit is hyperactive in 5xFAD mice and that sustained inhibition of this pathway alters multiple circuit and molecular features, we next examined whether this manipulation also affected reversal learning deficits in 5xFAD mice.

Mice underwent the instrumental task once daily for 30 days while corticostriatal inhibition was maintained (Fig. 6A). To minimize an effect of chemogenetic inhibition on memory consolidation shortly after each session^59,60^, C21 was administered at least six hours after the daily instrumental task (Fig. 7A). During the initial learning phase, both hM4Di- and mCherry-injected 5xFAD mice exhibited similar lever-pressing rates (Fig. 7B), and both groups preferentially pressed the valued over the devalued lever during the devaluation test (Fig. 7C), confirming intact acquisition of the initial action-outcome contingency.

In the reversal phase, lever-pressing rates remained comparable between groups (Fig. 7D). However, hM4Di-injected mice showed significantly more presses for the valued lever in the reversal devaluation test, while mCherry-injected controls did not (Fig. 7E). This indicated that only the treated group successfully acquired the reversed contingency. Importantly, hM4Di-injected mice exhibited a significantly higher reversal learning index than mCherry-injected controls (Fig. 7F), indicating improved cognitive flexibility following circuit inhibition.

Post-hoc comparisons showed that the reversal learning index was significantly lower than the initial learning index in mCherry-injected control mice (Fig. 7F), suggesting a reversal learning deficit in untreated 5xFAD mice. In contrast, hM4Di-injected mice showed no significant difference between their initial and reversal devaluation indices (Fig. 7F). This was consistent with the results observed in WT mice (Fig. 1G). Furthermore, hM4Di-injected mice exhibited a significantly higher reversal learning index than mCherry-injected controls (Fig. 7F). This suggested that sustained inhibition of the mPFC-to-DMS circuit effectively rescued the reversal learning deficits observed in young 5xFAD mice (Fig. 1 and Fig. 7).

We also evaluated broader cognitive performance using the novel object recognition test. While both groups preferred the novel object, hM4Di-injected 5xFAD mice displayed a marginally higher discrimination index than mCherry controls (Supplementary Fig. 14), indicating selective inhibition of DMS-projecting mPFC neurons enhanced recognition memory. Similarly, global mPFC inhibition by broadly expressing hM4Di in mPFC neurons also improved object recognition in 5xFAD mice (Supplementary Fig. 15A–D) without altering locomotor activity (Supplementary Fig. 15E and F) in 5xFAD mice. These findings support the broader cognitive benefits of reducing mPFC hyperactivity.

Taken together, these results indicate that sustained inhibition of the mPFC–DMS pathway, primarily involving PL/IL neurons projecting to the DMS, rescues reversal learning deficits in 5xFAD mice.

## Discussion

Cognitive flexibility, the ability to update action–outcome contingencies, is impaired early in AD. Here, we propose a circuit-based model in which early Aβ-associated cortical hyperactivity is associated with multiple circuit-level changes, including increased mPFC-to-DMS excitatory drive, elevated dMSN activity, enhanced inhibition of CINs, reduced striatal ACh release, and impaired reversal learning performance (Fig. 7G). Consistent with this framework, sustained chemogenetic inhibition of the mPFC-to-DMS circuit during early-stage AD reduces cortical Aβ accumulation, normalizes glutamatergic transmission in both the mPFC and DMS, and increases striatal cholinergic tone, while also improving reversal learning performance (Fig. 7H). Together, these findings identify corticostriatal hyperactivity as an early circuit alteration associated with both neurotransmitter changes, including cholinergic signaling, and deficits in behavioral flexibility in AD, and highlight this pathway as a potential target for early intervention.

Although striatal cholinergic dysfunction is linked to cognitive flexibility deficits in early-stage AD, the cholinergic hypothesis of AD focuses on the basal forebrain cholinergic system. This hypothesis is supported by postmortem studies showing significantly reduced cholinergic markers in the cortex and selective degeneration of the nucleus basalis of Meynert neurons in advanced AD^61,62^. However, the striatum also contains large densities of CINs that modulate cognitive flexibility in goal-directed behaviors^22,23,25,31^. Here, we report that 5xFAD mice exhibit deficits in striatum-dependent instrumental reversal learning, which precede the impairment of hippocampus-dependent spatial reversal learning. During instrumental reversal learning, 5xFAD mice acquired the initial action–outcome contingency normally, indicating that goal-directed behavior is preserved at this stage^63^. However, these mice exhibited marked impairments during the reversal phase, suggesting a deficit in updating previously learned action–outcome associations rather than a primary impairment in goal-directed control. Moreover, our findings were consistent with a previous report that spatial learning deficits emerged at around six months of age in 5xFAD mice^38^. These observations suggest that deficits in cognitive flexibility are more sensitive to striatal dysfunction than to dysfunction in the basal forebrain cholinergic neurons, with the latter predominantly innervating the hippocampus. Although we also imaged activity-trapped neurons in the hippocampus, we did not detect genotype-dependent differences in dentate gyrus activation during instrumental reversal learning. Hippocampal neurogenesis has been shown to support cognitive flexibility by reducing memory interference through inhibition of mature granule cells, thereby facilitating reversal learning in both neutral and aversive contexts^64^. The lack of detectable hippocampal differences in the present study is likely related to task demands, as instrumental reversal learning relies predominantly on striatal and fronto-striatal circuitry and may be less sensitive to hippocampal contributions.

The critical role of striatal cholinergic activity in early-stage AD could relate to the high levels of AChE in the striatum^65^. Changes in striatal ACh, such as those associated with corticostriatal hyperactivity, likely impair striatum-dependent executive functions, including instrumental reversal learning. Although the precise reason why striatal, rather than basal forebrain, cholinergic dysfunction predominates at this stage remains unclear, our findings suggest that CIN hypoactivity in 5xFAD mice is not due to cell loss but is consistent with excessive GABAergic inhibition by hyperactive dMSNs receiving elevated excitatory input from Aβ-enriched mPFC neurons. This multilevel circuit disinhibition alteration may compromise striatal output and contribute to behavioral rigidity. While mPFC-to-DMS circuit inhibition restores both cholinergic signaling and behavioral performance, the relative contribution of striatal cholinergic mechanisms to this rescue remains to be determined.

Our study also raises intriguing questions about the interaction between CINs and MSNs under pathological conditions. Reduced ACh release may impair striatal neuron activity via direct and indirect pathways. Prior work suggested that ACh indirectly inhibited MSNs via GABAergic interneurons such as NPY-expressing neurons^27,66,67^, suggesting that CIN hypoactivity could lead to aberrant MSN recruitment during reversal learning. Additionally, elevated corticostriatal transmission may potentiate maladaptive memory trace reactivation, as enhanced input to engram neurons can artificially strengthen memory retrieval^68^. These mechanisms may converge to explain the greater reactivation of cortical and striatal ensembles we observed during reversal learning in 5xFAD mice.

Mechanistically, our findings are consistent with the view that Aβ and neuronal hyperactivity form a pathological feedback loop. Activity-dependent Aβ release promotes further cortical hyperexcitability and impairs glutamate reuptake^10,12,56^. Consistent with this, we directly observed increased excitatory synaptic transmission in mPFC neurons from 5xFAD mice. In addition, we detected occasional large-amplitude spontaneous EPSC events in these neurons. Such events may reflect impaired glutamate clearance and consequent glutamate spillover, as suggested by previous studies, which could permit recruitment of neighboring synapses and amplification of excitatory currents. While Aβ accumulation is minimal in the striatum itself, the DMS receives dense glutamatergic input from Aβ-rich cortical areas. However, it is unclear how mPFC transmission to the striosome or matrix is altered under AD conditions^69^. The elevated mPFC-to-dMSN transmission observed in the present study using 5xFAD mice and in our  previous study using hAPP knock-in mice^70^ suggests that cortical hyperactivity can drive striatal circuit dysfunction across distinct AD mouse models, despite limited local Aβ. Our findings highlight the need to explore regions such as the striatum, which receives glutamatergic inputs from Aβ-rich cortical areas but is less affected by AD-related pathology.

Our study further demonstrates that sustained suppression of mPFC-to-DMS activity reduced cortical Aβ pathology. This was consistent with previous reports in hippocampal or entorhinal cortex regulation^57,58,71^. Moreover, we found that sustained mPFC-to-DMS suppression normalized excitatory transmission and resulted in elevated levels of ACh in the striatum. These effects were associated with improved reversal learning and recognition memory. Notably, general suppression of mPFC neurons produced a similar rescue of the circuit and behavior. This suggests that broad mPFC hyperactivity, rather than only within projection-defined subcircuits, can result in downstream dysfunction. Importantly, these effects persisted beyond the treatment period, indicating lasting circuit-level adaptations rather than transient pharmacological effects or simple learning-related changes. Early intervention to prevent Aβ accumulation in AD is important, as prolonged Aβ deposition can lead to lasting changes in corticostriatal circuits and even neuronal death, potentially limiting the clinical benefits of subsequent anti-Aβ treatments. While the development of anti-Aβ therapeutic antibodies continues, the findings of the present study indicate that reducing abnormal neuronal activity could provide a promising interim strategy for mitigating Aβ accumulation and reducing the associated cognitive deficits.

Although our study extends the characterization of early Alzheimer’s disease–related circuit dysfunction to the striatal circuitry, several limitations and important directions for future work should be considered. The ArcTRAP-based activity-labeling strategy used here captures neuronal populations engaged over extended behavioral time windows and lacks the temporal resolution required to resolve activity during discrete task epochs. Activity-dependent reporters with higher temporal precision, such as CaMPARI or Cal-Light, may enable more precise labeling of neurons activated during specific behavioral events in future studies^72–75^. In addition to functional circuit alterations, complementary structural analyses, including measurements of dendritic spine density and spine subtype composition, could provide further insight into early synaptic remodeling associated with Aβ pathology. Moreover, although instrumental reversal learning is an important component of executive function, the present data do not establish a generalized executive dysfunction phenotype at this stage and are limited to impaired updating of action–outcome contingencies in this task.

Additionally, although the present study focuses on circuit-level mechanisms, identifying the molecular substrates underlying altered neuronal excitability may help clarify how early circuit dysfunction emerges and may also suggest therapeutic targets for Alzheimer’s disease. The increased intrinsic excitability of mPFC pyramidal neurons observed here may reflect impaired repolarizing conductances, as prior studies have shown that dysfunction of Kv4.2- and Kv1-family potassium channels reduces outward currents and promotes neuronal hyperexcitability^76–80^. Reduced adaptation currents, governed by potassium and calcium channel activity, may likewise weaken spike-frequency adaptation and sustain cortical firing^81,82^. Enhanced corticostriatal glutamatergic transmission may also arise through multiple mechanisms spanning cortical, presynaptic, and postsynaptic levels. At the cortical level, impaired astrocytic glutamate clearance may contribute to increased mPFC activity, as Aβ has been shown to disrupt GLT-1/EAAT2-dependent glutamate uptake, prolong extracellular glutamate signaling, and promote synaptic spillover, thereby enhancing cortical excitability and increasing excitatory drive into the corticostriatal pathway^12,83^. At mPFC terminals, increased presynaptic release probability would be consistent with the reduced paired-pulse ratio observed here. At the postsynaptic level, altered AMPA receptor-mediated signaling in dMSNs may increase responsiveness to glutamatergic input and contribute to the elevated AMPA/NMDA ratio^84,85^. Together, these mechanisms may amplify corticostriatal excitation and bias dMSN activity toward persistent or maladaptive recruitment. Finally, although CIN hypoactivity in our model is most directly consistent with enhanced inhibitory input from hyperactive dMSNs, additional cholinergic mechanisms may further exacerbate this deficit. Muscarinic autoreceptors expressed on CINs, including M2-type receptors, regulate acetylcholine release and neuronal excitability; thus, their dysregulation could further suppress CIN firing under conditions of heightened inhibition^86,87^. Likewise, nicotinic acetylcholine receptors expressed on presynaptic terminals within striatal circuits modulate neurotransmitter release and network excitability, and their dysfunction or desensitization in Aβ pathology could impair cholinergic modulation and further reduce striatal cholinergic tone^65,88^. These possibilities remain speculative, but they provide testable molecular hypotheses linking early cortical hyperexcitability to downstream cholinergic dysfunction and cognitive inflexibility.

In summary, our study identifies a causal role for early AD-associated cortical hyperactivity in driving circuit and behavioral dysfunction and reveals downstream neurocircuit changes in the 5xFAD mouse model. These findings highlight the potential of circuit-based interventions to restore neuromodulatory balance and cognitive performance, particularly when applied at an early stage of disease progression before synaptic loss and neurodegeneration become widespread.

## Methods

### Animals

ChAT-eGFP (stock # 007902), 5xFAD (stock # 034848), ArcTRAP (#021881), Ai14- tdTomato (#007914), and Drd1a-tdTomato (stock # 016204) mice were purchased from the Jackson Laboratory. D1-Cre mice were obtained from Mutant Mouse Regional Resource Centers. All mice were backcrossed onto a C57BL/6 J background. ChAT-eGFP mice were crossed with 5xFAD to generate a 5xFAD;ChAT-eGFP line. 5xFAD;ChAT-eGFP mice were crossed with D1-Cre to generate D1-Cre;ChAT-eGFP;5xFAD line. D1- tdTomato mice were crossed with 5xFAD to generate a 5xFAD;D1-tdT mouse line. ArcTRAP mice were crossed with Ai14 to generate Arc;Ai14 mice. Arc;Ai14 mice were crossed with 5xFAD mice to generate triple transgenic Arc;Ai14;5xFAD mice. Mice were group-housed at 23 °C with a 12-h light:dark cycle (lights on at 11:00 pm). Relative humidity limits are set between 30% and 70%. Food and water were provided ad libitum. Both male and female mice were used, and sexes were balanced across experimental groups. Unless otherwise specified, mice used for electrophysiological recordings were 4–5 months old. All animal care procedures and experimental protocols were approved by the Texas A&M University Institutional Animal Care and Use Committee and were performed in accordance with the National Research Council Guide for the Care and Use of Laboratory Animals.

### Operant instrumental reversal learning tasks

This procedure was adapted from Bradfield and Balleine^31–33^, Ma and Huang^23^, and Gangal et al.^25^.

### Apparatus designs

The operant chambers (Med Associates Inc.) used in this study featured a central magazine for delivering rewards and two lever presses, one located on the left and the other on the right. A house light was also present. Each training session consisted of two sub-sessions, one involving the delivery of regular food pellets and the other involving purified food pellets, with the order of the sub-sessions selected randomly. A 2.5-minute time-out period separated the sub-sessions. Training sessions lasted a maximum of 62.5 minutes, with each sub-session ending at 30 minutes or when 20 rewards were earned, whichever came first. During the acquisition phase, the house light was on, and one of the lever presses was available for use, whereas during the time-out session, the house light was off, and all lever presses were retracted. The training schedule included four phases: initial learning, initial devaluation testing, reversal learning, and reversal devaluation testing.

### Acquisition of initial contingencies

Training began with either a purified or grain pellet sub-session, with the house light on and one of the lever presses presented. During initial training, the contingency is that the left lever (A1) results in a grain pellet reward (O1), and the right lever (A2) results in a purified pellet reward (O2). Animals were trained on a fixed ratio 1 (FR1, with one lever press for one reward delivery) schedule for 3 days, where one lever press triggered one reward delivery. They then progressed to a variable ratio schedule, starting with a ratio of 5 (RR5, with a probability of 0.2 for reward delivery) for 3 days, followed by RR10 (with a probability of 0.1 for reward delivery) for 3 days, and finally reaching RR20 (with a probability of 0.05 for reward delivery) for five days.

### Initial devaluation test

The devaluation test was conducted 24 h after the last initial RR20 training. Before the test, animals were given one h of free access to 40 randomly assigned purified or grain pellets to induce satiety. Ten min after the animal consumed all the pellets, or 60 min later, whichever came first, the animals were placed into the operant chamber where the house light and both lever presses were presented. Lever presses during the 10-min testing period were recorded and did not trigger reward delivery. The lever for the pre-feed pellet is considered a devaluated (Dev) lever, whereas the lever for a non-pre-feed pellet is considered a valued (Val) lever. The same devaluation test procedure for the other pellet type was conducted the following day.

### Reversal learning

Following a 2-day initial devaluation test, animals received an additional day of training on the initial RR-20 schedule and were then transitioned to training under the reversal contingency, beginning with a reversal RR-20 schedule. In this procedure, the action-outcome contingency was reversed from the initial action-outcome contingency, where the left lever (A1) was paired with purified pellet (O2), which was previously paired with grain pellet (O1), and the right lever (A2) was paired with grain pellet (O1), which was previously paired with purified pellet (O2), for five sessions.

### Reversal devaluation test

The procedure for this devaluation test was the same as that for the initial devaluation test. The devaluation index is calculated as (Val-Dev)/(Val+Dev).

### Post-feeding satiety (free-choice) test

After each outcome devaluation session, mice were returned to their home cage and immediately given simultaneous access to both outcomes (grain-based pellets and purified pellets; 50 pellets each). Animals were allowed to consume freely for 10 min. Pellets remaining were counted at the end of the session, and consumption for each outcome was calculated as the number of pellets consumed (50 − remaining).

### Progressive ratio (PR) test

Motivation for food reinforcement was assessed using a progressive ratio schedule. During the PR session (60 min; same duration as training sessions), the response requirement increased according to the following breakpoint series: 1, 3, 7, 12, 20, 32, 51, 78, 118, 178, 268, 403, 603, 902, 1348, 2013, 3000. Only one lever, associated with either grain or purified pellet, was tested per session. Breakpoint was defined as the highest ratio requirement completed within the 60-min session.

### ArcTRAP approach

The procedure followed previously described protocols, with details and modifications described below^69,89,90^. The mice were habituated to receiving saline injections (i.p.) for three consecutive days before receiving 4-OHT (50 mg/kg, i.p.) injection. The 4-OHT solution was freshly prepared on the tagging day. In brief, the 4-OHT powder was dissolved in 200-proof ethanol (20 mg/mL) at room temperature, with continuous shaking until the 4-OHT was fully dissolved. An equal volume of a 1:4 mixture of castor oil and sunflower seed oil was added, resulting in a final concentration of 10 mg/mL 4-OHT, and the ethanol was removed by centrifugation under vacuum. The final 4-OHT in oil was stored at 4 °C for a maximum of 12 h before use. The 4-OHT was administered immediately following either the completion of the middle two initial RR20 sessions or after the reversal RR20 sessions. After completing the operant behavioral session, mice were placed in an isoflurane chamber and briefly anesthetized. While under isoflurane anesthesia, 4-OHT was administered intraperitoneally to minimize pain and distress associated with the injection of the oil-based solution. Following the injection, mice were returned to their home cages to recover and were left undisturbed for the subsequent 6 h. No behavioral testing was performed during or immediately after anesthesia. Three weeks post-tagging, the mice were perfused. Subsequently, their brain slices were imaged using confocal microscopy.

### Confocal imaging and cell counting

Mice were intracardially perfused with 4% paraformaldehyde (PFA) in phosphate-buffered saline (PBS) 14 days after Arc;Ai14 labeling of activated neurons to allow sufficient time for replication and transcription^91^. The brains were extracted and post-fixed overnight in a 4% PFA/PBS solution, followed by dehydration in 30% sucrose. Each whole brain was sectioned serially into 50-µm coronal slices using a cryostat. Confocal images were obtained using a confocal laser-scanning microscope (Fluoview 3000, Olympus). Fluorescent images were reconstructed in three dimensions, and cell counts from these scans were manually acquired using Bitplane Imaris 8.3.1 (Bitplane, Zurich, Switzerland), as previously reported^92^. Neurons were counted using the Spot module within Imaris, which also calculated colocalization. To calculate the density of neurons, we conducted neuron counting in a circular region of interest (ROI) created in Imaris within the brain region. Neuron density is determined by dividing the number of neurons by the area of the defined ROI. All neuronal counts were performed with experimenters blinded to experimental conditions. Brain structures were registered using the Paxinos mouse atlas as a reference^93^.

### Barnes maze

The Barnes maze followed previously described protocols, with details and modifications described below^94^. The Barnes circular platform maze consists of a 66 cm diameter circular platform elevated on a 1.4 m stand. It features 20 evenly spaced 5.08 cm diameter holes around the circumference, with a black box (escape tunnel) positioned beneath one of the holes (San Diego Instruments, San Diego, CA, USA). Briefly, in each trial, the subject was initially placed at the center of the maze and given a maximum of 3 minutes to find the target escape hole. The trial ended when the mouse entered the escape hole. Spatial cues were present in the walls surrounding the maze to assist the animal in identifying the target location. A bright light was paired with the start of the experiment, and the light was turned off when the mouse reached the escape or at the end of each trial if the escape was not found. The test comprised four sessions: initial learning, probe testing, reversal learning, and reversal probe testing. During initial and reversal learning, mice underwent 4 consecutive training days, with 4 trials per day and a 15-min intertrial interval. In the reversal learning session, the location of the target escape hole was shifted 180 degrees opposite of its position during initial learning. In both probe tests, the escape hole was removed, permitting mice to freely explore for 3 min. The entire test was monitored with an overhead camera and analyzed using video-tracking software (Ethovision XT, Noldus). The analysis included measures of velocity (cm/s), distance traveled (cm), immobility (%), and latency (s).

### Radial maze

Spatial discrimination and reversal learning were assessed using an elevated 8-arm radial arm maze. All behavioral testing was conducted during the light phase under consistent room lighting and stable distal visual cues. The maze was cleaned between animals using an unscented disinfectant to minimize odor cues. Animal position, arm entries, and occupancy were recorded using EthoVision XT (Noldus), with the central platform and each arm defined as regions of interest.

Mice were maintained under mild food restriction throughout behavioral testing and kept at ~85–90% of their free-feeding body weight. Grain-based food pellets were used as rewards.

### Arm assignment

The eight arms of the maze were assigned to three functional categories: baited (reward) arms (*n* = 3), dummy (reversal) arms (*n* = 3), and control arms (*n* = 2). During the acquisition phase, baited arms contained food pellets, while dummy and control arms contained no pellets. During reversal learning, the dummy arms became the baited (rewarded) arms, while the original baited arms and control arms were not baited. Control arms were never baited during either phase.

### Habituation (2 days)

Mice underwent two days of habituation to reduce novelty-induced anxiety and promote exploration of the maze. During each habituation session, mice were placed on the central platform and allowed to freely explore the maze for 2 min. One grain pellet was placed in each arm (8 pellets total) to encourage entry into all arms. Mice were returned to their home cages at the end of each session.

### Acquisition training (4 days)

Following habituation, mice were trained to learn a fixed spatial reward contingency. At the start of each trial, five grain pellets were placed at the distal end of each of the three baited arms (15 pellets total per trial). Dummy arms and control arms contained no pellets. Mice were placed on the central platform and allowed to explore the maze for 2 min. Animals completed two trials per day during the acquisition phase. After each trial, remaining pellets were counted, and the number of pellets consumed was calculated as a measure of reward collection and motivation.

### Reversal learning (4 days)

To assess cognitive flexibility, reward contingencies were reversed following acquisition. The three dummy arms became the baited arms, with five grain pellets placed at the distal end of each, while the original baited arms and control arms contained no pellets. Reversal trials were conducted using the same trial duration (2 min) and reward configuration as during acquisition. Mice completed two trials per day for the first two days of reversal learning, followed by one trial per day for the final two days, to assess the persistence of reversal learning.

### Behavioral analysis

Behavioral performance was quantified using time spent in each arm as measured by EthoVision XT. Time spent in baited, dummy, and control arms was used as the primary measure of spatial learning and reversal performance. Reward collection was quantified by counting the number of pellets consumed during each trial.

### Locomotor activity test

The locomotor activity test followed previously described protocols, with details and modifications described below^95^. A transparent, open-field activity chamber (Med Associates, 43 cm × 43 cm × 21 cm height) was equipped with an infrared beam detector connected to a computer. Animals were moved to the locomotor activity test room 30 minutes before testing. The distance traveled and velocity were analyzed using Activity Monitor software (MED Associates, St Albans, VT). This test was conducted right after the last devaluation test of reversal instrumental learning, the final Barnes maze test, or 24 hours after the last C21 injection.

### Chemogenetic activation

A DREADD agonist, Compound 21 (C21) (dissolved in saline, 1 mg/kg, i.p.), was injected once every 24 h for 30 continuous days at 3 months old of 5xFAD mice. For those 5xFAD animals that need to train with reversal learning, C21 injection was conducted 6 h after instrumental training.

### Novel Object Recognition Test

The test was comprised of three parts: habituation, familiarization, and testing.

Habituation: On the same day as the locomotor activity test, animals were given 10 min to explore an empty arena, an opaque, uncapped, cube-shaped chamber measuring 43 cm × 43 cm × 21 cm in height.

Familiarization: Twenty-four h after habituation, the animals were exposed to the familiar arena with two identical objects for 10 min.

Testing: Twenty-four h after familiarization, the mice were allowed to explore an open field containing both the familiar object and a novel object to assess long-term recognition memory. The test mouse was given 5 min to explore the chamber freely, and its behavior was monitored using an overhead camera and video-tracking software (Ethovision XT, Noldus). Ethovision was used to analyze the time spent in the ‘novel object zone’ and ‘familiar object zone,’ as well as the number of entries into each zone. The interaction zone is defined as the 2.5 cm area around each object.

### Stereotaxic virus infusion

Stereotaxic viral infusions followed previously described protocols, with details and modifications described below^96–99^. The skin was opened to uncover the skull and expose the bregma and lambda, and the location of the desired injection site was determined. A three-axis micromanipulator was used to measure the spatial coordinates for bregma and lambda. Small drill holes were made in the skull at the appropriate coordinates, according to the Paxinos atlas. Brain infusions were performed at a rate of 0.1 µL/min. Two microinjectors were loaded with 0.5 µL of AAV2/9-hSyn- GRAB_gACh4m_ (BrainVTA, PT-7021), AAV-hsyn-rACh1.7-WPRE-hGH-polyA (BrainVTA, PT-5488), or rAAV2Retro/CAG-Cre (UNC, AV7703H) and were bilaterally injected into the striatum (AP: 0.26 mm, ML: ± 2.00 mm, DV: −3.50 mm). rAAV8/hSyn-hM4Di-mCherry (UNC, AV5360c), rAAV8/hSyn-mCherry (UNC, AV6443D), AAV9-hsyn-DIO-hM4Di-mCherry (Addgene, 44362), or AAV9-hsyn-DIO-mCherry (Addgene, 50459) were bilaterally injected into the mPFC, prelimbic cortex (PL/IL), region (AP: 1.94 mm, ML: ± 0.35 mm, DV: −2.20 mm) in 5xFAD or WT control mice. 0.5 µL of rAAV8/svn-ChR90-GFP(Svn-Chronos-GFP) (UNC, AV5842E) was bilaterally injected into the mPFC region in 5xFAD;D1-tdT or D1-tdT mice. 0.5 µL of rAAV9/syn-Flex-ChrimsonR-tdT (UNC, AV6556B) was infused bilaterally into the DMS of D1-Cre;ChAT-eGFP; 5xFAD or D1-Cre;ChAT-eGFP mice. To avoid backflow of the virus, microinjectors were left in place for 10 min after the infusion was complete and then removed. The skin was sutured, and the mice were allowed to recover for at least 1 week prior to further experiments.

### Electrophysiological recordings of brain slices

#### Brain slice preparation

The brains were promptly removed, and slices of the striatum were cut 250 µm thick in an ice-cold cutting solution. The cutting solution consisted of the following (in mM): 40 NaCl, 143.5 sucrose, 4 KCl, 1.25 NaH_2_PO4, 26 NaHCO_3_, 0.5 CaCl_2_, 7 MgCl2, 10 glucose, 1 sodium ascorbate, and 3 sodium pyruvate, and had a pH of 7.35 and an osmolarity of 305-310 mOsm. The solution was saturated with 95% O_2_ + 5% CO_2_. Subsequently, the slices were incubated at 32 °C for 45 min in a 1:1 mixture of the cutting and external solutions. The external solution, which had a pH of 7.35 and an osmolarity of 305-310 mOsm, was composed of the following (in mM): 125 NaCl, 4.5 KCl, 2 CaCl_2_, 1 MgCl_2_, 1.25 NaH_2_PO_4_, 25 NaHCO_3_, 15 sucrose, and 15 glucose. The external solution was also saturated with 95% O_2_ + 5% CO_2_. The slices were maintained in the external solution at room temperature until needed.

#### Electrophysiological recordings

The brain slice was positioned in a recording chamber attached to the fixed stage of an upright microscope (Olympus). The slice was then perfused with an oxygenated external solution at 32 °C, with a 2 mL/min flow rate. Neurons were visualized using a 40x water-immersion lens and an infrared-sensitive CCD camera. A Multiclamp 700B amplifier with Clampex 10.6 software and Digidata1550A data acquisition system (Molecular Devices in Sunnyvale, CA) was employed for recording purposes. Recording pipettes with a resistance of 3-6 MΩ were made from a borosilicate glass capillary (World Precision Instruments, Sarasota, FL) by using a micropipette puller (Model P-97, Sutter Instrument Co).

To measure electrically evoked EPSCs in cortical neurons or dMSNs, a bipolar glass stimulating electrode was placed in the same compartment. Electrical stimulation was delivered as single biphasic pulses (200 μs duration) at 0.05 Hz. Input–output relationships were assessed by applying a range of stimulation intensities. Cesium intracellular solution was used for EPSC recordings; this contained (in mM) 119 CsMeSO_4_, 8 TEA.Cl, 15 HEPES, 0.6 ethylene glycol tetraacetic acid (EGTA), 0.3 Na_3_GTP, 4MgATP, 5 QX-314.Cl and 7 phosphocreatine, with the pH adjusted to 7.3 using NaOH. For optically-evoked EPSC (oEPSC) recordings, 2 ms flash of light via an objective lens was used (470 nm for Chronos and 590 nm for Chrimson stimulation). Picrotoxin (PTX, 100 μM; Cat. No. 1128, Tocris) was added to the ACSF during EPSC recordings.

For the measurement of AMPAR/NMDAR ratio, the peak currents of AMPAR-mediated EPSCs were measured at a holding potential of –70 mV, and the NMDAR-mediated EPSCs were estimated as the EPSCs at + 40 mV, 30 ms after the peak AMPAR EPSCs, when the contribution of the AMPAR component was minimal. The AMPA/NMDA ratio was calculated by dividing the AMPAR EPSCs by the NMDAR EPSCs.

To measure electrically evoked IPSCs (eIPSCs) or optically evoked IPSCs (oIPSCs) in cholinergic interneurons (CINs), a high-chloride intracellular solution was used. This solution contained the following components (in mM): 125 CsCl, 6 NaCl, 10 HEPES, 1 EGTA, 10 QX-314.Cl, 2 MgATP, 0.6 Na₃GTP, and 2 Na₂CrPO₄. The pH was adjusted to 7.25, and the osmolarity was set at 280 mOsm. GABA currents (I_GABA_) in CINs were detected under whole-cell voltage-clamp mode (hold potential: −60 mV), and a high-chloride intracellular solution was used. To measure the I_GABA_ currents in CINs, CINs were held at −60 mV in ACSF for 1 min for baseline recording and 5 mL of GABA (50 μM) was applied. Holding currents were measured every 5 s. DNQX (10 μM; Cat. No. 0189, Tocris) was added to the ACSF during IPSC recordings.

Cell-attached voltage-clamp or whole-cell current-clamp techniques were performed to record spontaneous action potentials of CINs without antagonists in the external solution. Whole-cell current-clamp was performed to record evoked firing. The pipette was filled with a K^+^-based intracellular solution consisting of 123 potassium gluconate, 10 HEPES, 0.2 EGTA, 8 NaCl, 2 MgATP, and 0.3 NaGTP (pH 7.25, 280 mOsm). Unlike whole-cell patch-clamp recordings, used to clamp neurons close to −70 mV, cell-attached patch-clamp was used to record neurons firing without a holding potential when the resistance exceeded 1 GΩ. Evoked action potentials were measured with whole-cell current-clamping while increasing current injections from 0 to 500 pA in incremental steps of 1-s.

Two consecutive optical or electrical stimuli with fixed intervals (e.g., 50 or 100 ms), based on neuron type and experimental conditions, were applied to generate two post-synaptic currents. The peak amplitudes of the first (P1) and second (P2) responses were measured, and the paired-pulse ratio (PPR) was calculated as the ratio of P2 to P1. A minimum of three trials per condition were averaged for statistical analysis.

All patch-clamp recording data were analyzed using Clampfit (in pClamp 10.7, Molecular Devices), except that the sEPSC/sIPSC data were analyzed in the Mini Analysis Program.

### Immunohistochemistry

Aβ immunostaining was conducted as previously described^100^. Antigen exposure was performed using 90% formic acid (Sigma, SHBQ5806). Non-specific proteins were blocked using 10% goat serum (Jackson ImmunoResearch, AB_2336990). Cortical sections of 50 μm thickness were labeled using rabbit anti-Aβ overnight at 4 °C (Invitrogen, 71-5800, LOT YB369857, diluted at 1:300 in 2% goat serum of 0.5% PBS-Triton™ X-100) (Fisher BioReagents, BP151-500) (PBS-TX). This was followed by incubation with Alexa Fluor 647 conjugated goat anti-rabbit IgG (H + L) antibody (Invitrogen, A21245, LOT 2291604, diluted 1:500 in 2% goat serum of 0.5% PBS-TX) for 2 h at room temperature and three washes in 0.5% PBS-TX. Quantifying the percentage area covered by Aβ plaques and the size of Aβ plaques was performed using ImageJ (NIH).

For the c-Fos immunostaining, sections were first placed into 0.5% PBS-TX (PBST) for 10 min, followed by blocking in 10% Bovine Serum Albumin (BSA) (Sigma, A7030) in PBS-TX for 1 h and incubation in primary antibody (rabbit anti-cFos,1:2000, EMD-Millipore, cat# ABE457, LOT 3988907) diluted in blocking solution overnight under 4 degrees. Sections were then incubated in 1:500 biotinylated donkey anti-rabbit (Jackson Immuno Research #711-065-152, LOT 165233) for 1 h. Sections were visualized via incubation for 1 h in Streptavidin-conjugated Alexa 647 (1:1000, Thermo Fisher, #21374, LOT 2291604, 2 mg/ml).

### Ex vivo live-tissue confocal imaging of ACh release

Acute brain slices were imaged in a custom-made chamber using an Olympus FluoView FV3000 confocal microscope, with ACSF flowing and saturated with 95% O_2_ and 5% CO_2_. The confocal microscope was equipped with a 10x NA 0.3 and a 40x NA 0.8 water immersion objective, along with a 488 nm and a 561 nm laser. The sample rate of imaging is 2-3 frames per second. Imaging parameters were consistently maintained across all imaging sessions, including laser intensity, HV, gain, offset, and aperture diameter. Electro-stimulation was administered using a glass pipette containing a tungsten filament. DF/F, z score, and half-width were analyzed and obtained by MATLAB.

### 6-Hz seizure threshold testing

The 6 Hz model of partial seizures was employed to determine the seizure-inducing threshold in mice, following established protocols^101,102^. Corneal stimulation consisted of monopolar rectangular pulses (0.2-millisecond duration) delivered at 6 Hz for 3 sec using a constant-current device (World Precision Instruments, Sarasota, FL). To establish the EC50 value, the stimulation current required to induce seizures in 50% of animals, various current intensities ranging from 10 to 70 mA were administered to distinct groups of mice. As an anti-pruritic, 0.5% tetracaine ocular anesthetic was applied to the corneas 10 min before stimulation. Just prior to stimulation, the corneal electrodes were moistened with 0.9% saline solution. Mice were manually restrained during the 6 Hz stimulation and released immediately afterward into an observation chamber. Behavioral seizure severity was assessed using the Racine scale^103^: stage 0: normal behavior; stage 1: chewing and facial twitches; stage 2: head nodding or shaking; stage 3: unilateral forelimb clonus without rearing, straub tail, and extended body posture; stage 4: bilateral forelimb clonus with rearing; stage 5: wild jumping and tonic-clonic activity. A minimum interval of 15 min was allowed before increasing the current intensity for subsequent stimulation. Seizures induced by the 6-Hz model typically lasted between 8 and 90 sec, with mice resuming normal exploration within seconds of seizure cessation.

### EEG electrode implantation

On the day of surgery, animals were weighed, anesthetized with a ketamine (100 mg/kg) and xylazine (10 mg/kg) mixture, and mounted on a stereotaxic frame. A vertical incision was made between the ears to expose the skull. Two-wire anchor screws were implanted: one served as a surface electrode in the cortex and the other as a cerebellar reference electrode. The screws were secured to the skull with dental acrylic (P1 Technologies, Roanoke, VA). After a 10-day recovery period, the mice were single-housed in the vivarium prior to the pilocarpine challenge. Cortical recordings were made from the animals.

### Pilocarpine seizure test

Animals were pretreated with scopolamine methyl bromide (1 mg/kg, subcutaneous, Sigma-Aldrich, St. Louis, MO) 30 min prior to pilocarpine injection to enhance survival rates without affecting seizure severity. Pilocarpine (416 mg/kg, intraperitoneal, Sigma-Aldrich, St. Louis, MO) was then administered to induce persistent seizures and status epilepticus in the mice^104^.

### Video EEG and behavior recording

Behavior and EEG activity were continuously monitored for 2 h after pilocarpine injection to assess seizure progression. A 30-min baseline EEG recording was conducted prior to the experiment using a digital EEG system. EEG signals were recorded with ClampEx-pCLAMP software connected to Grass Technologies P511 AC preamplifiers, sampled at 2000 Hz, and filtered through a Digidata 1440 A to reduce noise. Video recordings of the EEG analysis were made with 1080p HD digital security cameras equipped with infrared LEDs for night vision. Both cortical and hippocampal EEG channels were recorded. Mice were awake and freely moving in cages fitted with swivels. Pilocarpine-induced EEG seizure activity was characterized by high-amplitude discharges (at least twice the baseline amplitude) and repetitive discharges ( > 0.5 Hz). The severity of behavioral seizures was rated using the Racine scale described earlier.

### Statistical analysis

Data were analyzed using two-sided t-tests or two-way RM ANOVA followed by Sidak post-hoc tests, as appropriate. Normality and equal variance assumptions were assessed. Mann–Whitney U tests, Wilcoxon signed-rank tests, unpaired t-tests with Welch’s correction, mixed-effects models, or generalized linear mixed models (GLMMs) were used when data did not meet parametric assumptions or when required by the experimental design. Greenhouse–Geisser correction was applied when the sphericity assumption was not met for two-way repeated-measures ANOVA. Statistical significance was set at *p* < 0.05. Statistical analyses were performed using SigmaPlot 12.5, GraphPad Prism 10, Origin 2026, or IBM SPSS Statistics GradPack 29. Graphs were generated using Origin 2026. Data are presented as mean ± SEM. Behavioral and histological data were analyzed at the level of individual animals. Live confocal imaging data were analyzed at the slice level, and electrophysiological data were analyzed at the neuron level; corresponding animal numbers are provided in the figure legends.

### Reporting summary

Further information on research design is available in the Nature Portfolio Reporting Summary linked to this article.

## Supplementary information

Supplementary Information

Reporting Summary

Transparent Peer Review file

## Source data

Source Data

## Data Availability

The source data generated in this study are provided in the Source Data file. This Excel file contains multiple worksheets, including the numerical data used to generate Figs. 1–7 and Supplementary Fig. 1–15, together with associated statistical information. No restricted-access datasets were used in this study. Source data are provided with this paper.

## Code availability

Custom MATLAB analysis code used to analyze live confocal imaging data is available at GitHub: (https://github.com/chen-ruifeng/ex-vivo-confocal).
