# Supplementary material for: Early-Stage Corticostriatal Hyperactivity Impairs Cognitive Flexibility Alongside Striatal Cholinergic Dysfunction in an Alzheimer’s Disease Model
Source: Nat Commun. 2026 Jun 26;17:8226. doi: 10.1038/s41467-026-74817-z (PMC13462761; doi:10.1038/s41467-026-74817-z)
Supplement: Supplementary file 1 [file 41467_2026_74817_MOESM1_ESM.pdf]

Supplementary Figures

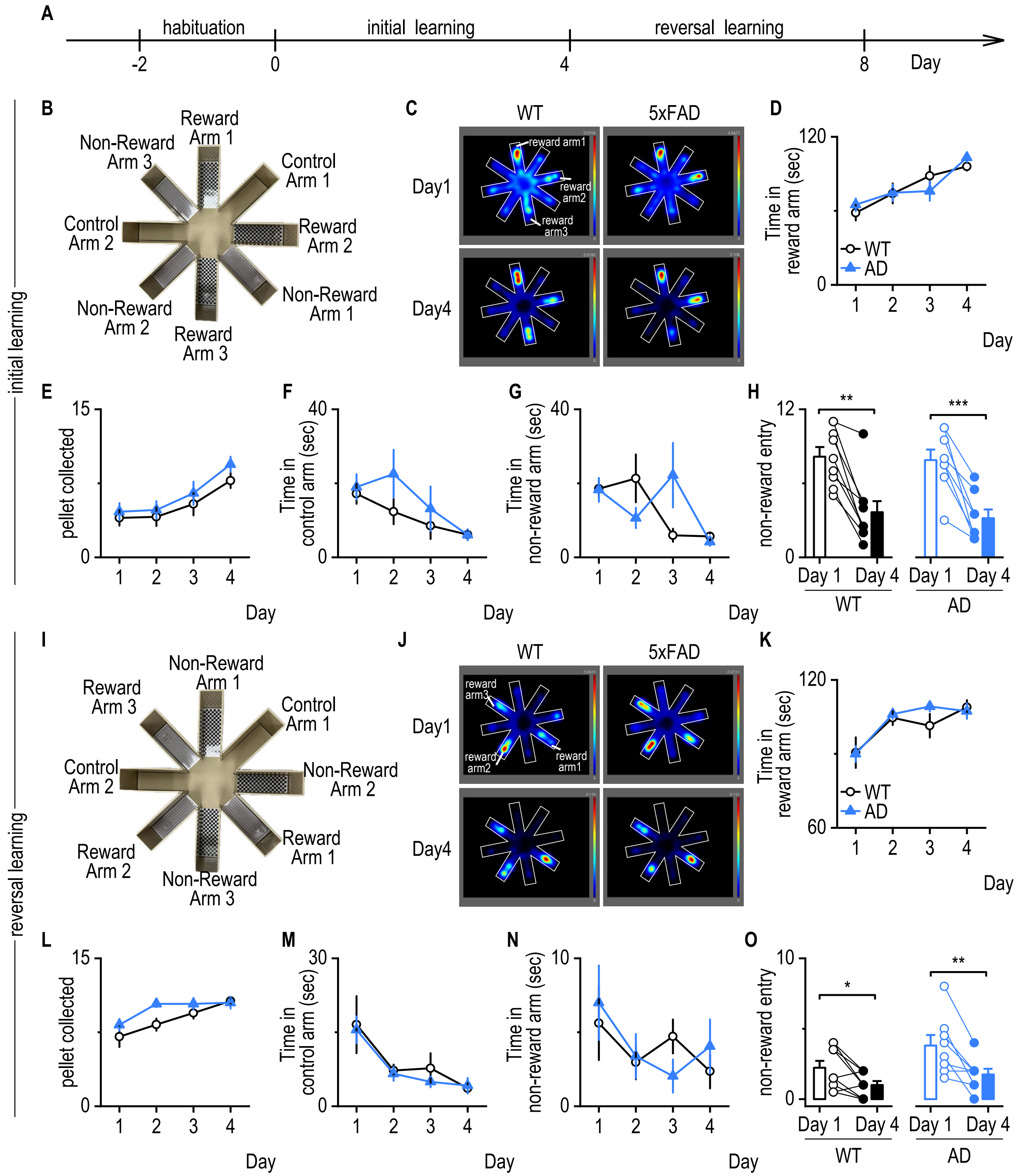

Supplementary Figure 1. 5xFAD mice did not exhibit deficits in reversal learning in a rewarded eight-arm radial maze at 3 months of age.

A, Experimental timeline.

B, Schematic of initial learning maze configuration, with three reward arms, three non-reward arms and two control arms.

C, Representative heat maps on days 1 and 4 of initial learning.

D–G, Initial learning performance during the initial phase, including time spent in reward arms (D), pellets collected (E), time spent in control arms (F), and time spent in non-reward arms (G). Two-way RM ANOVA, genotype: $F_{(1,15)} = 0.0061$, $p = 0.9390$ (D); $F_{(1,15)} = 0.7323$, $p = 0.4056$ (E); $F_{(1,15)} = 1.010$, $p = 0.3310$ (F); $F_{(1,15)} = 0.1114$, $p = 0.7432$ (G).

H, Time in non-reward arms decreased from day 1 to day 4. Wilcoxon signed-rank test for WT, W = −45.00, $p = 0.0039$; paired t-test for 5xFAD, $t_7 = 5.693$, $p = 0.0007$. $^{**}p < 0.01$, $^{***}p < 0.001$.

I, Schematic of reversal learning maze configuration, with reward and non-reward arm identities reassigned.

J, Representative heat maps on days 1 and 4 of reversal learning.

K–N, Reversal learning performance during the reversal learning phase, including time spent in reward arms (K), pellets collected (L), time in control arms (M), and time in non-reward arms (N). Two-way RM ANOVA, genotype: $F_{(1,15)} = 0.2265$, $p = 0.6410$ (K); $F_{(1,15)} = 2.142$, $p = 0.1639$ (L); $F_{(1,15)} = 0.0113$, $p = 0.9166$ (M); $F_{(1,15)} = 0.1445$, $p = 0.7092$ (N).

O, Time in non-reward arms decreased from day 1 to day 4 during reversal learning during the reversal learning phase. Wilcoxon signed-rank test for WT, W = −37.00, $p = 0.0312$; paired t-test for 5xFAD, $t_7 = 3.667$, $p = 0.0080$. $^{*}p < 0.05$, $^{**}p < 0.01$.

n = 9 (WT) and 8 (5xFAD) for all panels. Data are mean ± SEM; tests were two-sided, with adjusted P values where applicable. Source data are provided as a Source Data file.

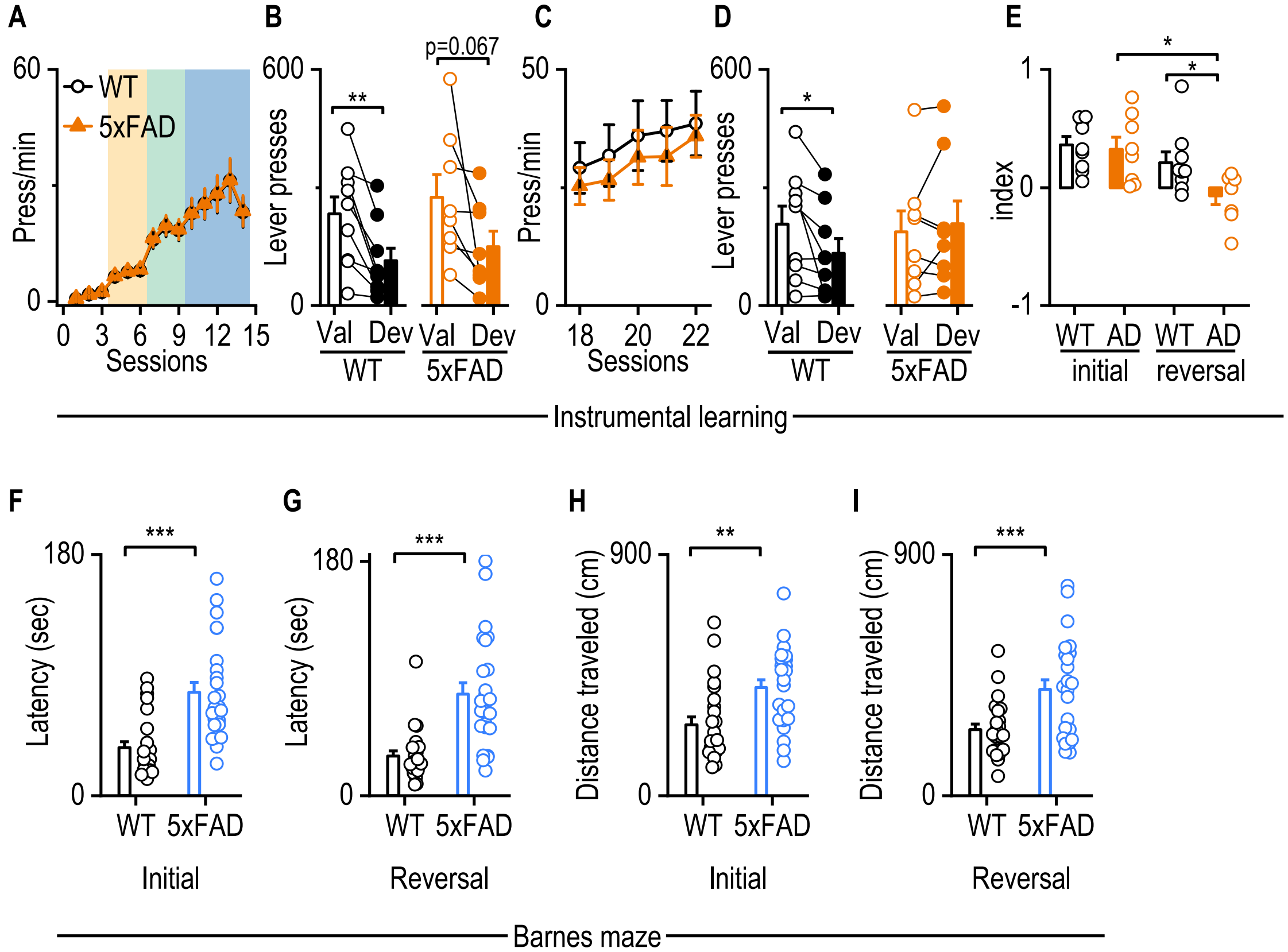

Supplementary Figure 2. 5xFAD mice exhibited reversal learning deficits in instrumental learning and Barnes maze test at 6 months of age.

A, 6-month-old WT and 5xFAD mice did not differ in lever pressing rates during the initial learning. Two-way RM ANOVA. $F_{(1,15)} = 0.266$, $p = 0.614$.

B, WT mice displayed fewer lever presses for devalued than valued outcomes during the initial devaluation test, and 5xFAD mice exhibited a similar trend. Paired t-test, $t_8 = 4.059$, $p = 0.0036$ for WT; $t_8 = 2.169$, p = 0.067 for AD. $^{**}p < 0.01$.

C, WT and 5xFAD animals had similar lever pressing rates during reversed learning. Two-way RM ANOVA, $F_{(1,15)} = 0.305$, $p = 0.589$.

D, WT, but not 5xFAD, mice displayed fewer lever presses for devalued than valued outcomes during the reversal devaluation test. Paired t-test, $t_8 = 2.569$, $p = 0.0332$ for WT; $t_7 = 0.843$, $p = 0.427$ for AD. $^{*}p < 0.05$.

E, 5xFAD mice displayed a significantly lower reversal index than their initial devaluation index and the reversal devaluation index of WT mice. Two-way RM ANOVA, $F_{(1,15)} = 4.504$, $p = 0.051$; post-hoc analysis, $^{*}p < 0.05$ for sessions within AD. $^{*}p < 0.05$.

F and G, 6-month-old 5xFAD mice spent more time finding the escape box than WT mice did in both initial (F, U = 90, $p < 0.0001$) and reversal sessions (G, U = 78, $p < 0.0001$). Mann-Whitney test, $^{***}p < 0.001$.

H and I, The total traveled distance from the starting point to the escape box by 6-month-old 5xFAD mice was higher than that of WT mice in both initial (H, $U = 144$, $p = 0.0008$) and reversal sessions (I, $t_{48} = -3.538$, $p = 0.0009$). Mann-Whitney test (H) or unpaired t-test (I), $^{**}p < 0.01$, $^{***}p < 0.001$.

n = 9 WT and 8 5xFAD for panels A-E; n = 25 WT and 5xFAD for panels F-I. Data are mean ± SEM; tests were two-sided, with adjusted P values where applicable. Source data are provided as a Source Data file.

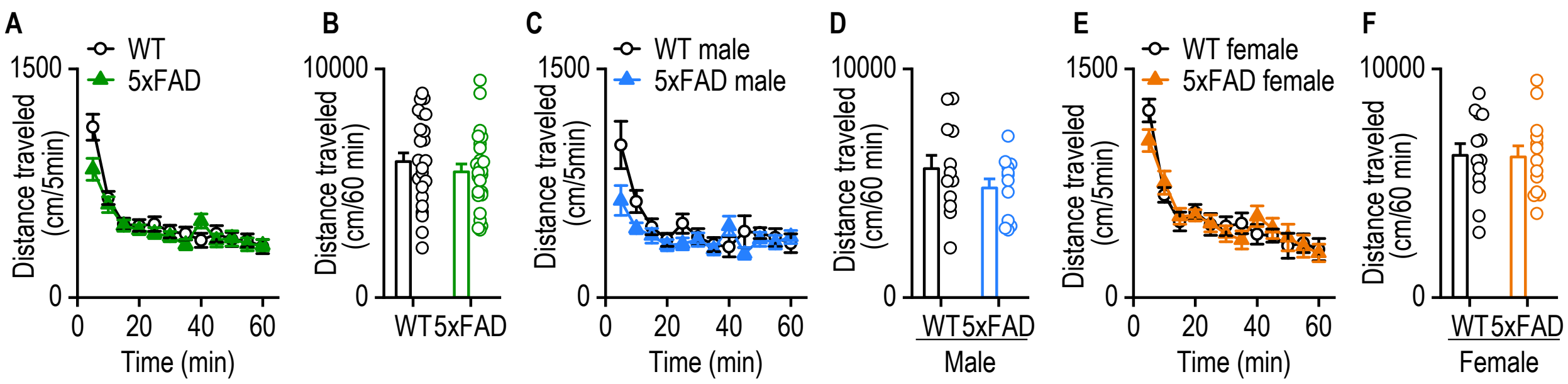

Supplementary Figure 3. 5xFAD mice did not exhibit abnormal locomotor activity.

A, **B**, Similar spontaneous locomotor activities were shown in WT and 5xFAD mice in time-course (A, $F_{(1, 48)}$ = 0.7615, p = 0.3872) or the total traveled distance (B, t_{48} = 0.873, p = 0.3872). Two-way RM ANOVA (A) and unpaired t-test (B). n = 25 WT and 5xFAD.

C-F, There were no differences in spontaneous locomotor activities between males (C, $F_{(1, 22)}$ = 1.466, p = 0.2388; D, t_{22} = 1.211, p = 0.2388) or females (E, $F_{(1, 24)}$ = 0.009951, p = 0.9214; F, t_{24} = 0.0988, p = 0.9214) 5xFAD mice and their WT control. Two-way RM ANOVA (C and E) and unpaired t-test (D and F). n = 12 male WT and AD; 13 female WT and 5xFAD.

Data are mean ± SEM; tests were two-sided, with adjusted P values where applicable. Source data are provided as a Source Data file.

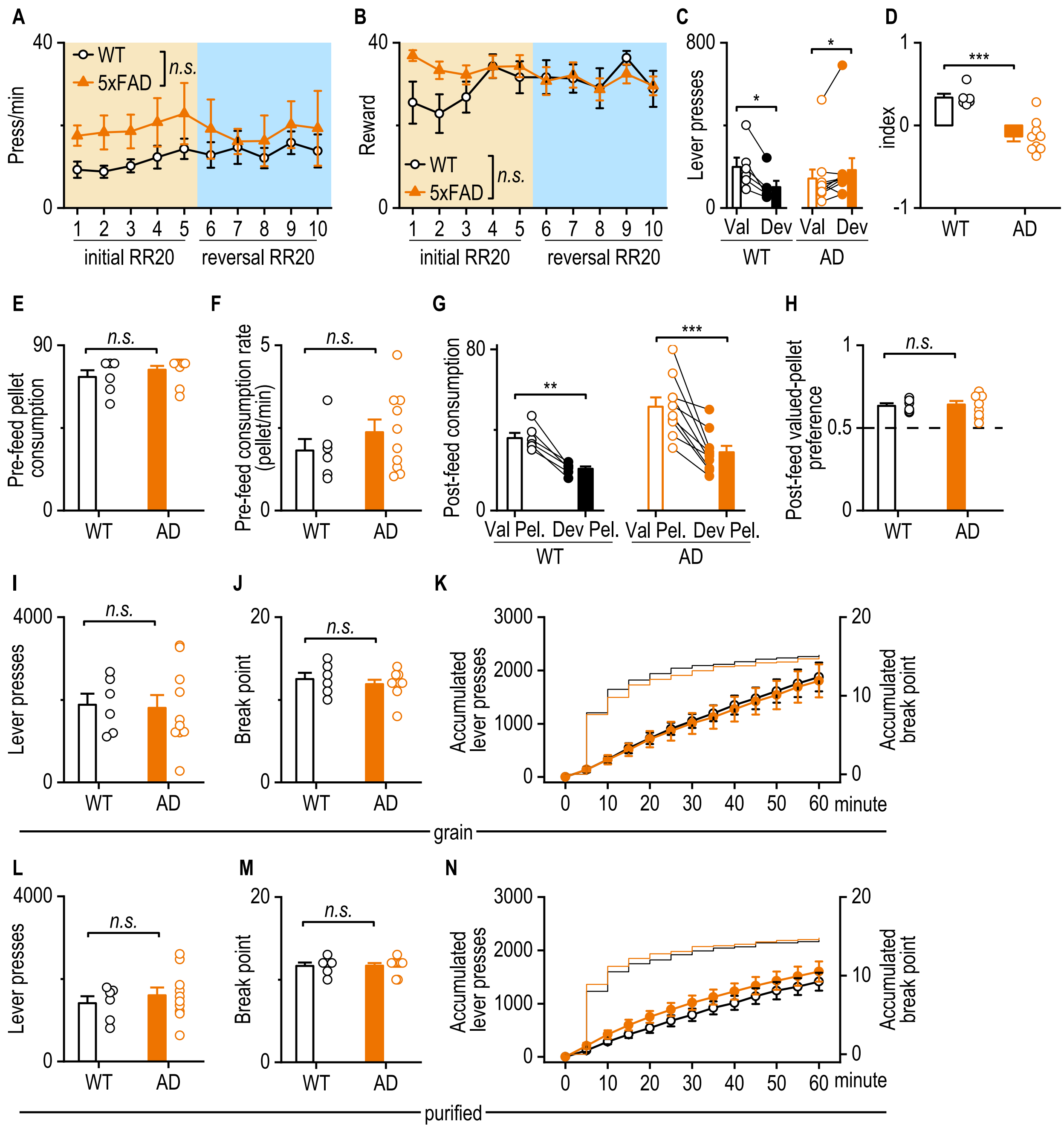

Supplementary Figure 4. Reversal learning and satiety/motivation control analyses in WT and 5xFAD mice.

A separate WT/5xFAD cohort was used to assess reversal learning in the absence of prior outcome devaluation and to evaluate satiety and motivational controls.

A, Lever-press rates during initial and reversal RR20 training. Two-way RM ANOVA, genotype: $F_{(1,14)} = 0.8776$, $p = 0.3647$.

B, Reward collection during initial and reversal RR20 training. Two-way RM ANOVA, genotype: $F_{(1,14)} = 0.6548$, $p = 0.4319$.

C, Lever presses during the reversal devaluation test. Wilcoxon signed-rank test, $W = -21.00$, $p = 0.0312$ (WT); $W = -40.00$, $p = 0.0391$ (5xFAD). $^{*}p < 0.05$.

D, Devaluation index from lever presses. Mann–Whitney test, $U = 2$, $p = 0.0010$. $^{***}p < 0.001$.

E, Number of pellets consumed during the 1-h pre-feed period (80 pellets total, two pellet types). Mann–Whitney test, $U = 23$, $p = 0.4268$.

F, Pre-feed consumption rate (pellets/min) during the pre-feed period. Unpaired t-test, $t_{14} = 0.9802$, $p = 0.3436$.

G, Post-pre-feed free-choice consumption of non-prefeed (valued pel.) and prefeed (devalued pel.) pellets. Paired t-test, $t_5 = 6.696$, $p = 0.0011$ (WT); $t_9 = 5.119$, $p = 0.0006$ (5xFAD). $^{**}p < 0.01$, $^{***}p < 0.001$.

H, Post-pre-feed valued-pellet preference index (valued pellet consumption/ total pellet consumption). Unpaired t-test, $t_{14} = 0.2601$, $p = 0.7986$.

I, Total lever presses in progressive ratio (PR) test for grain pellets. Unpaired t-test, $t_{14} = 0.1544$, $p = 0.8795$.

J, Breakpoint achieved in PR test for grain pellets. Mann–Whitney test, $U = 25.5$, $p = 0.6430$.

K, Cumulative lever pressing across the PR session for grain pellets.

L, Total lever presses in PR test for purified pellets. Unpaired t-test, $t_{14}= 0.6828$, $p = 0.5059$.

M, Breakpoint achieved in PR test for purified pellets. Mann–Whitney test, $U = 29$, $p = 0.9685$.

N, Cumulative lever pressing across the PR session for purified pellets.

n = 6 (WT) and 10 (5xFAD) mice for all panels. Data are mean ± SEM; tests were two-sided, with adjusted P values where applicable. Source data are provided as a Source Data file.

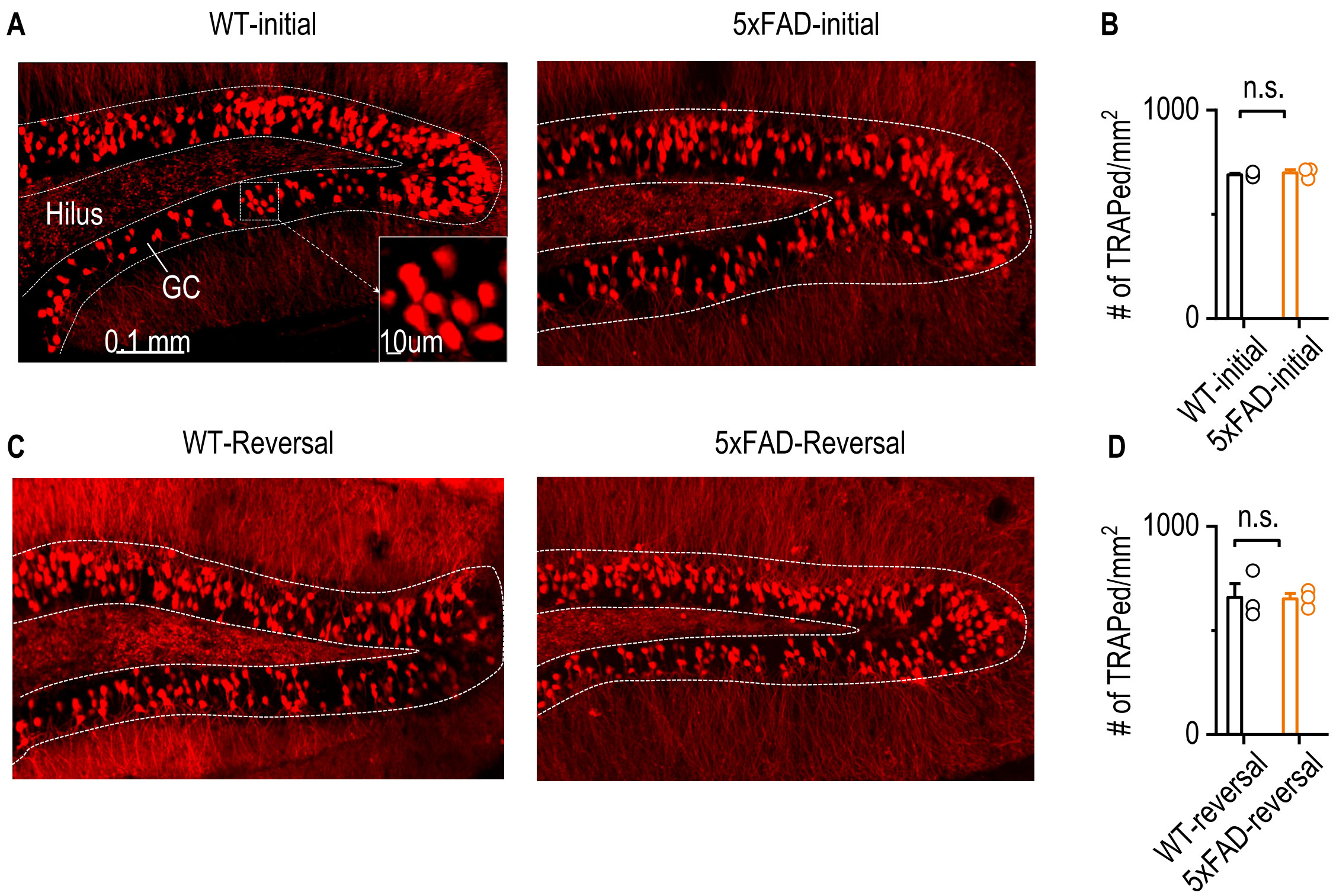

Supplementary Figure 5. Trapped hippocampal neurons do not differ between WT and 5xFAD in either initial or reversal learning.

A, Representative images showing trapped neurons in the dentate gyrus (DG) of the hippocampus following initial learning in WT and 5xFAD mice. GC, granule cell layer.

B, Quantification of tdTomato-labeled DG neurons during initial learning revealed no significant difference between WT and 5xFAD mice. $t_4 = 0.5449$, $p = 0.6148$, unpaired t-test. n = 3 mice for both WT and 5xFAD.

C, Representative images showing trapped DG neurons during reversal learning in WT and 5xFAD mice.

D, Quantification of labeled DG neurons during reversal learning showed no significant difference between WT and 5xFAD mice. $t_4 = 0.1146$, $p = 0.9143$, unpaired t-test. n = 3 mice for both WT and 5xFAD.

Data are mean ± SEM; tests were two-sided, with adjusted P values where applicable. Source data are provided as a Source Data file.

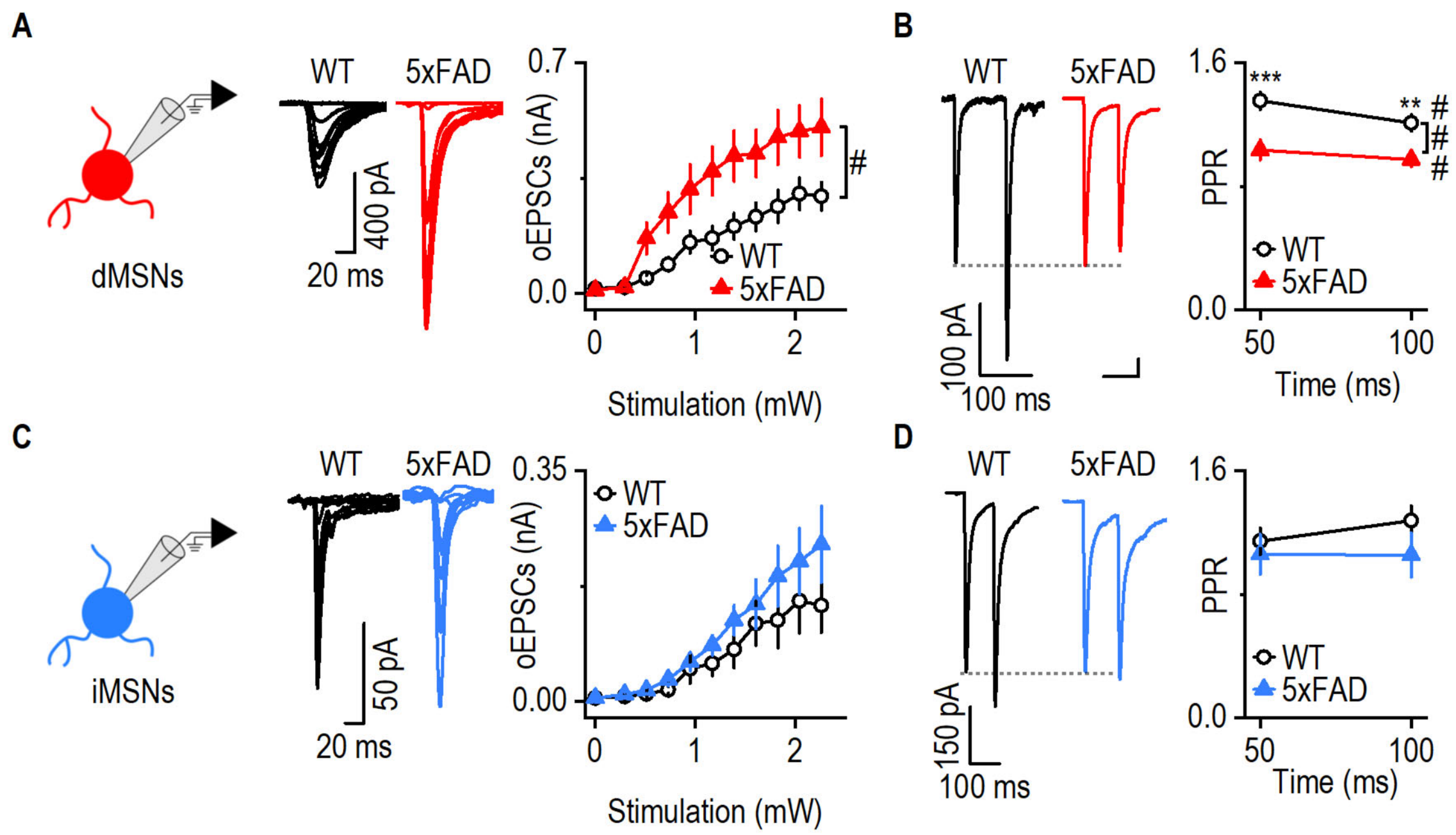

Supplementary Figure 6. The mPFC-to-dMSN circuit is hyperactive in 12-month-old 5xFAD mice.

A, oEPSC amplitude in DMS dMSNs was greater in 12-month-old 5xFAD mice than WT controls. Two-way RM ANOVA, $F_{(1,32)} = 5.422$, $p = 0.026$, $^{\#}p < 0.05$. n = 17 neurons from 3 mice (WT and 5xFAD).

B, PPRs of oEPSCs in DMS dMSNs were smaller in 12-month-old 5xFAD mice than age-matched WT controls. Two-way RM ANOVA, $F_{(1,27)} = 18.17$, $p = 0.0002$, $^{\#\#\#}p < 0.001$; Sidak's multiple comparisons post-hoc analysis, $^{**}p < 0.01$, $^{***}p < 0.001$, versus WT at the same time interval. n = 15 neurons from 3 mice (WT) and 14 neurons from 3 mice (5xFAD).

C, oEPSC amplitudes in DMS iMSNs were not significantly different between 12-month-old 5xFAD and WT mice. Two-way RM ANOVA, $F_{(1, 19)} = 1.167$, $p = 0.2935$. n = 9 neurons from 2 mice (WT) and 12 neurons from 3 mice (5xFAD).

D, PPRs of oEPSCs in iMSNs did not differ between 5xFAD and WT mice. Two-way RM ANOVA, $F_{(1, 14)} = 0.8648$, $p = 0.3682$. n = 6 neurons from 2 mice (WT) and 10 neurons from 3 mice (5xFAD).

Data are mean ± SEM; tests were two-sided, with adjusted P values where applicable. Source data are provided as a Source Data file.

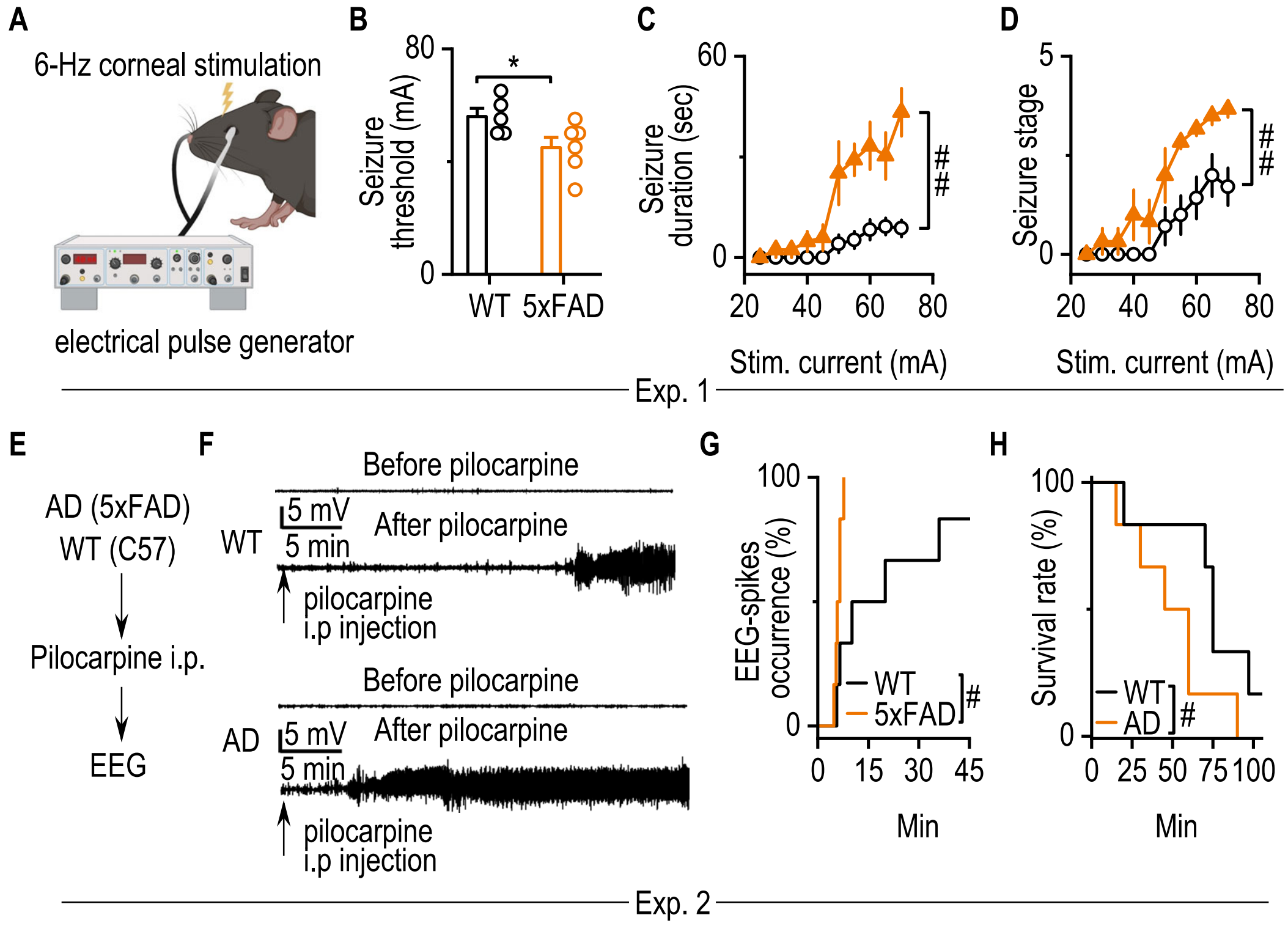

Supplementary Figure 7. 5xFAD mice exhibit heightened induced seizure activity at 6 months old.

A, Schematic illustrating 6-Hz corneal stimulation in mice. Corneal stimulation consisted of monopolar rectangular pulses (0.2-millisecond duration) delivered at 6 Hz for 3 sec using a constant-current device.

B, 5xFAD mice displayed a significantly lower threshold for seizure induction compared to WT controls. $t_9 = 2.283$, $p = 0.0484$, unpaired t-test. $^{*}p < 0.05$. n = 6 (5xFAD) and 5 (WT) mice.

C, 5xFAD mice had significantly longer seizure durations than WT controls. Two-way RM ANOVA, $F_{(1,11)} = 15.85$, $p = 0.0022$. $^{\#\#}p < 0.01$. n = 6 (5xFAD) and 7 (WT) mice.

D, 5xFAD mice exhibited increased seizure stages compared to WT controls. Two-way RM ANOVA, $F_{(1,11)} = 12.89$, $p = 0.0042$. $^{\#\#}p < 0.01$. n = 6 (5xFAD) and 7 (WT) mice.

E, Schematic of the experimental design for an additional cohort of AD and WT mice in Exp. 2. Pilocarpine was administered intraperitoneally to induce seizure behavior, and EEG spikes were recorded from the cortex.

F, Sample trace in EEG recordings showing spikes before and after pilocarpine i.p. injection.

G, 5xFAD mice displayed a higher percentage of seizure spike occurrences in EEG recordings within 45 min after pilocarpine injection compared to WT controls. Kaplan-Meier estimates followed by the Tarone-Ware test, Chi-Square = 4.44035, $p = 0.0351$, $^{\#}p < 0.05$. n = 6 mice per group.

H, Survival rate following seizure induction was significantly lower in 5xFAD mice compared to WT controls. Kaplan-Meier estimates followed by the Tarone-Ware test, Chi-Square = 4.46955, $p = 0.0345$, $^{\#}p < 0.05$. n = 6 mice/group.

Data are mean ± SEM; tests were two-sided, with adjusted P values where applicable. Source data are provided as a Source Data file. Panel A was created in BioRender. Huang, Y. (2026) https://BioRender.com/kh5jchl.

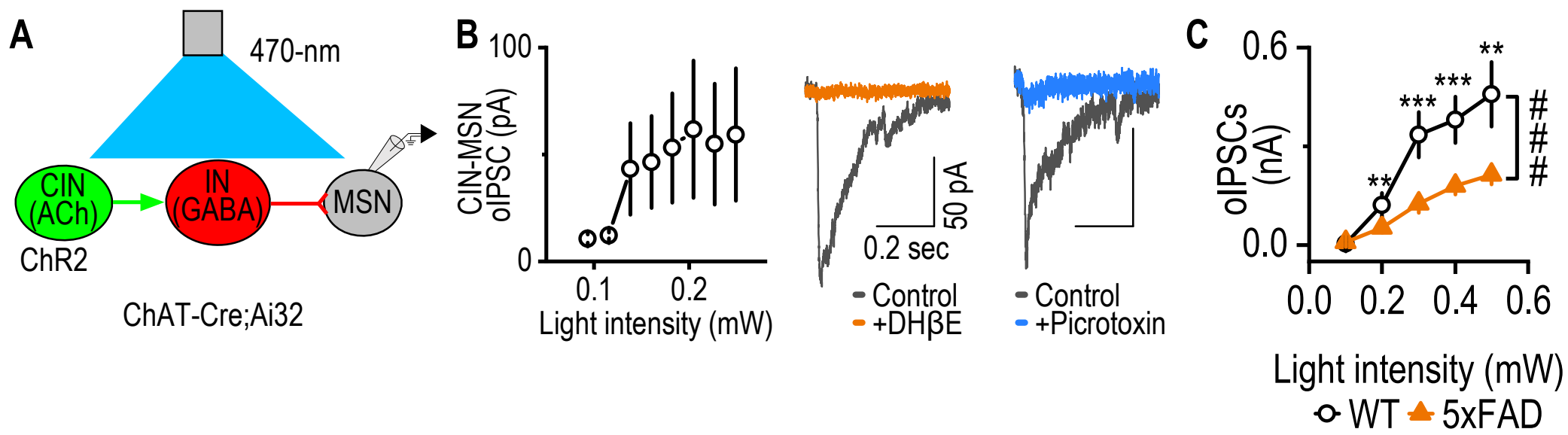

Supplementary Figure 8. Reduced CIN-to-MSN inhibition in 5xFAD mice.

A, Schematic illustrating the stimulation and recording of CIN-to-MSN inhibitory transmission in DMS slices from 4-month-old ChAT-Cre;Ai32 mice. IN, interneuron.

B, Representative CIN-to-MSN inhibitory postsynaptic currents (IPSCs, left) were blocked by the nicotinic receptor antagonist DhβE (1 μM) and the $GABA_A$ receptor antagonist picrotoxin (0.1 mM), confirming both cholinergic and GABAergic components. n = 7 neurons from 2 mice.

C, CIN-to-MSN IPSC amplitudes were significantly reduced in 5xFAD mice compared to controls. $F_{(1, 39)} = 24.72$, $p < 0.0001$, $^{###}p < 0.001$, Two-way RM ANOVA; $^{**}p < 0.01$, $^{***}p < 0.001$ WT versus 5xFAD at the same stimulating intensities, Sidak's multiple comparisons post-hoc analysis. n = 20 neurons from 3 mice (Ctrl; ChAT-Cre;Ai32) and 21 neurons from 3 mice (5xFAD;ChAT-Cre;Ai32).

Data are mean ± SEM; tests were two-sided, with adjusted P values where applicable. Source data are provided as a Source Data file.

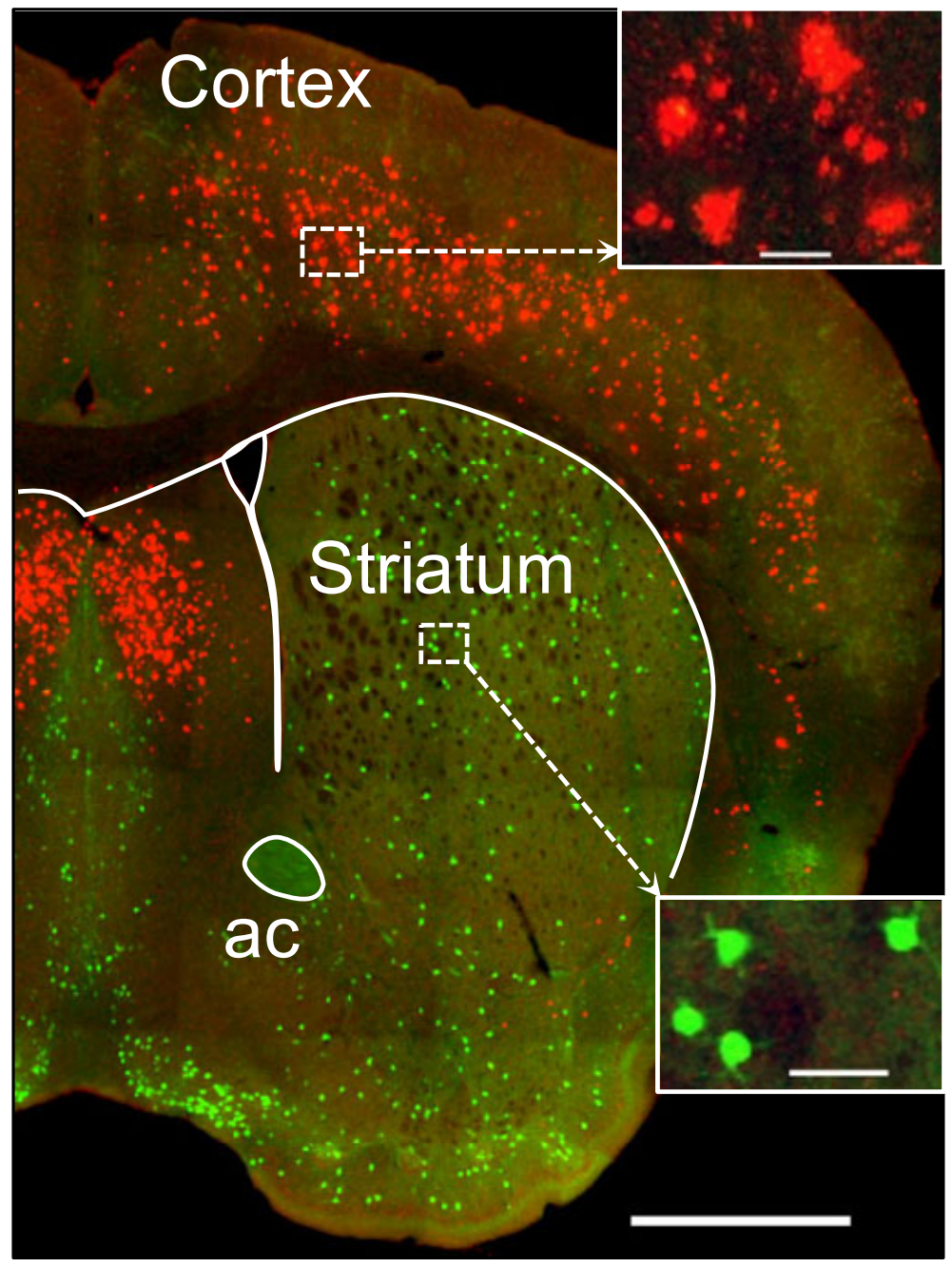

Supplementary Figure 9. Aβ deposition in the cortex but not the striatum of 3-month-old 5xFAD;ChAT-eGFP mice.

Representative images showing Aβ (red) accumulation in the cortex, with no detectable Aβ signal in the dorsal striatum. Cholinergic interneurons (CINs) are labeled with eGFP (green). ac, anterior commissure. Scale bars: 1 mm (main panel), 50 μm (inset).

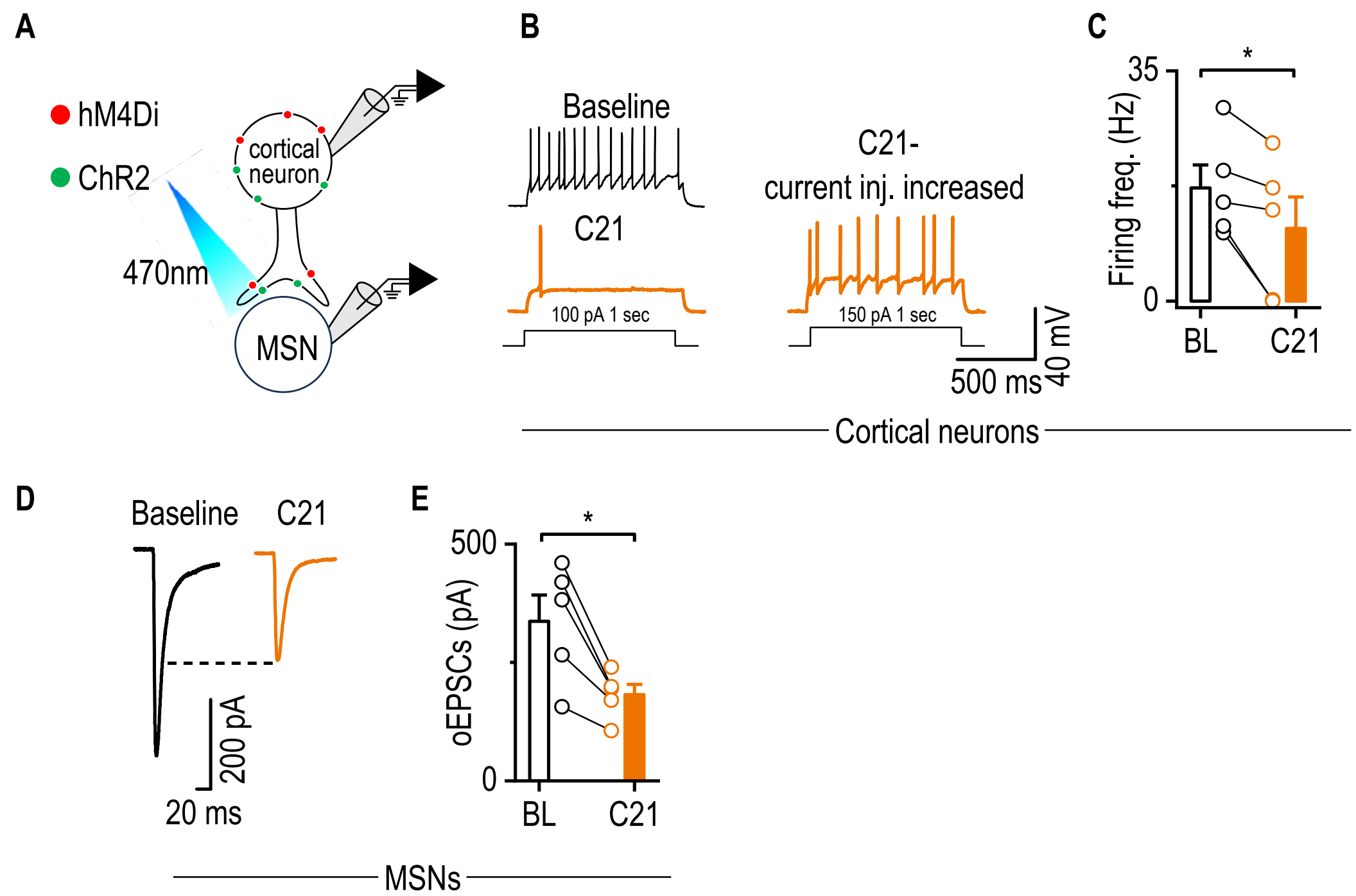

Supplementary Figure 10. hM4Di activation reduces neuronal excitability and synaptic transmission.

A, Schematic illustrating AAV infusion and patch-clamp recording strategy. AAV-hSyn-ChR2-GFP and AAV-hM4Di-mCherry were co-infused into the mPFC. Recordings were obtained from mPFC neurons co-expressing ChR2 and hM4Di, and from postsynaptic MSNs in the DMS that did not express either construct.

B, Sample trace showing decreased excitability of hM4Di-expressing mPFC neuron after hM4Di activation.

C, hM4Di activation significantly reduced excitability. Paired t-test, $t_4 = 3.049$, $p = 0.0381$. $^*p < 0.05$. n = 5 neurons from 1 animal.

D, Sample trace showing decreased oEPSC amplitude after hM4Di activation.

E, hM4Di activation significantly reduced oEPSC amplitude. Paired t-test, $t_4 = 4.463$, $p = 0.0111$. $^*p < 0.05$. n = 5 neurons from 1 animal.

Data are mean ± SEM; tests were two-sided, with adjusted P values where applicable. Source data are provided as a Source Data file.

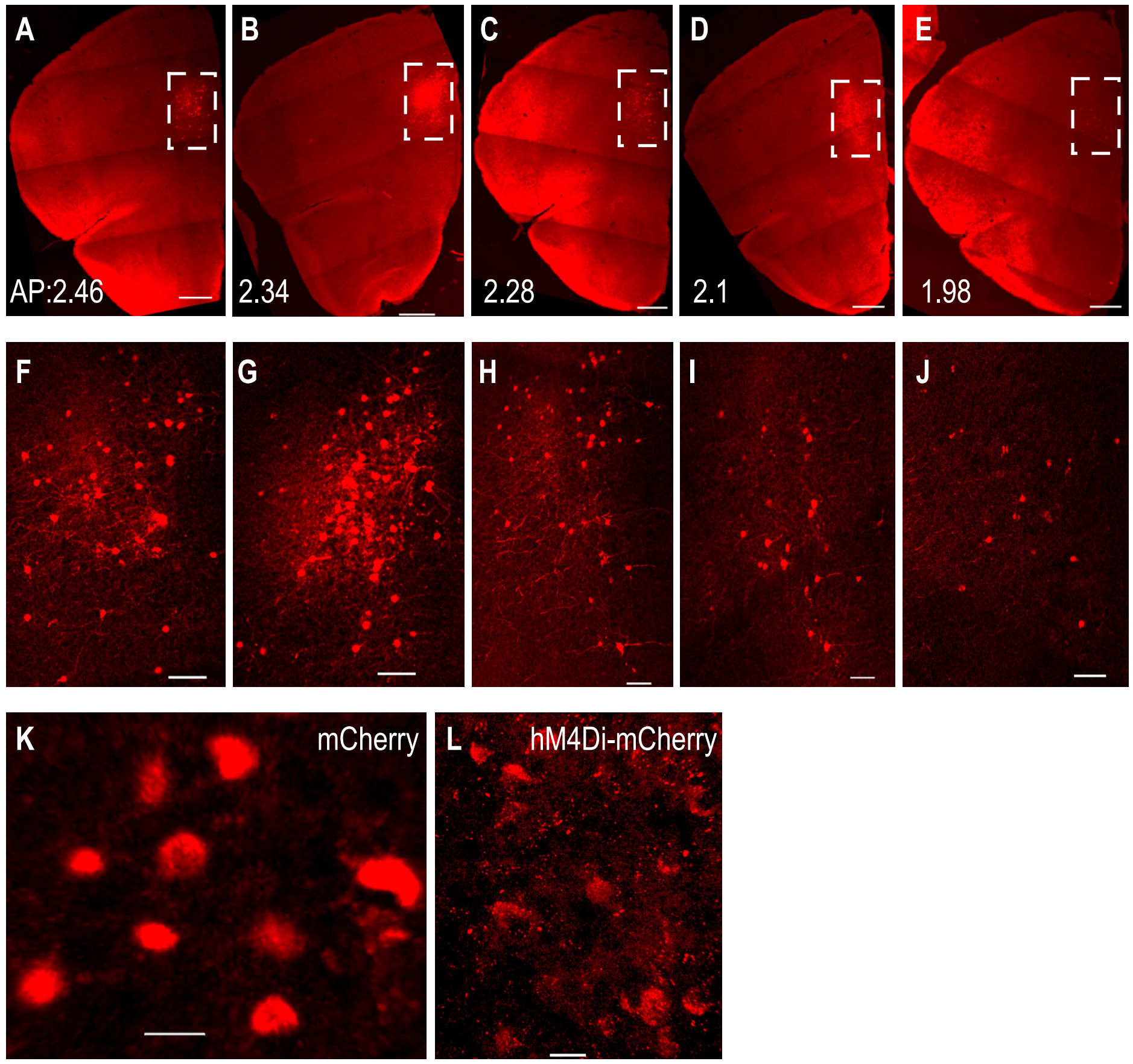

Supplementary Figure 11. Confocal images showing the location and spread of mCherry-positive neurons.

A-E, Coronal sections of the mPFC displaying mCherry expression from bregma AP 2.46 to 1.98 mm. Scale bar: 500 μm.

F-J, Enlarged views of the dashed boxes in A–E, showing mCherry-positive neurons. Scale bar: 100 μm.

K and L, High-magnification images comparing mCherry expression in animals infused with AAV-DIO-mCherry (K) and AAV-DIO-hM4Di-mCherry (L). Scale bar: 20 μm.

Panels A–J were obtained from a mouse infused with AAV-DIO-mCherry.

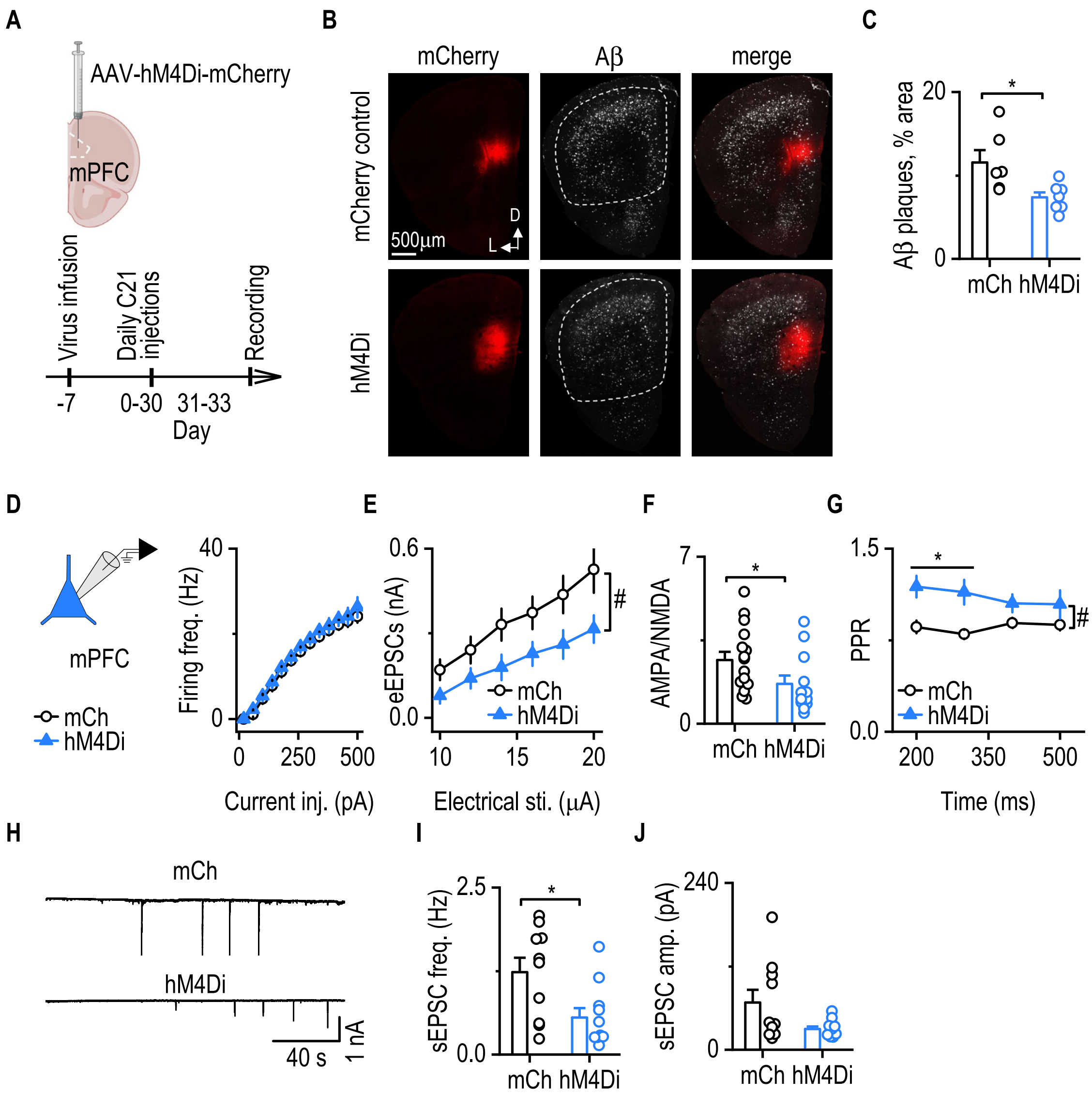

Supplementary Figure 12. Sustained chemogenetic inhibition of cortical neurons normalizes glutamatergic transmission and results in lower Aβ accumulation in the cortex.

A, Experimental timeline. At 3 months of age, 5xFAD mice received mPFC infusion of AAV-hM4Di-mCherry or AAV-mCherry, followed by daily C21 injections (1 mg/kg) for 4 weeks before behavioral testing, electrophysiological recordings and Aβ staining.

B, Representative images showing Aβ staining and quantification regions (dashed area) in hM4Di- and mCherry-injected 5xFAD mice.

C, Aβ plaque coverage was lower in hM4Di-injected mice than mCherry controls. Unpaired t-test, t_{11} = 2.751, p = 0.0188. $^{*}p < 0.05$. n = 6 mice (mCh); 7 mice (hM4Di).

D, Excitability of mCherry-positive mPFC neurons did not differ between groups. Two-way RM ANOVA, $F_{(1,24)}$ = 0.1278, p = 0.7239. n = 13 neurons from 4 mice (mCh and hM4Di).

E, eEPSC amplitudes in mCherry-positive mPFC neurons were lower in hM4Di-injected mice than mCherry controls. Two-way RM ANOVA, $F_{(1,32)}$ = 4.835, p = 0.035, $^{\#}p < 0.05$. n = 15 neurons from 4 mice (mCh) and 19 neurons from 4 mice (hM4Di).

F, AMPA/NMDA ratios were lower in hM4Di-injected mice than in mCherry controls. Mann-Whitney test, U = 44, p = 0.015, $^{*}p < 0.05$. n = 16 neurons from 4 mice (mCh) and 12 neurons from 4 mice (hM4Di).

G, PPRs of mCherry-positive mPFC neurons were higher in hM4Di-injected mice than in mCherry controls. Two-way RM ANOVA, $F_{(1,33)}$ = 7.153, p = 0.0114; $^{\#}p < 0.05$; post-hoc, $^{*}p < 0.05$ at matched intervals. n = 16 neurons from 4 mice (mCh) and 19 neurons from 4 mice (hM4Di).

H, Representative sEPSC traces from mCherry-positive mPFC neuron.

I and J, sEPSC frequency was lower in hM4Di- than mCherry-injected mice. Unpaired t-test, t_{19} = 2.691, p = 0.0145 (I); Welch's unpaired t-test, $t_{9.709}$ = 2.072, p = 0.0659 (J); n = 10 neurons from 4 mice (mCh) and 11 neurons from 4 mice (hM4Di).

Data are mean ± SEM; tests were two-sided, with adjusted P values where applicable. Source data are provided as a Source Data file. Panel A was created in BioRender. Huang, Y. (2026) https://BioRender.com/kh5jchl.

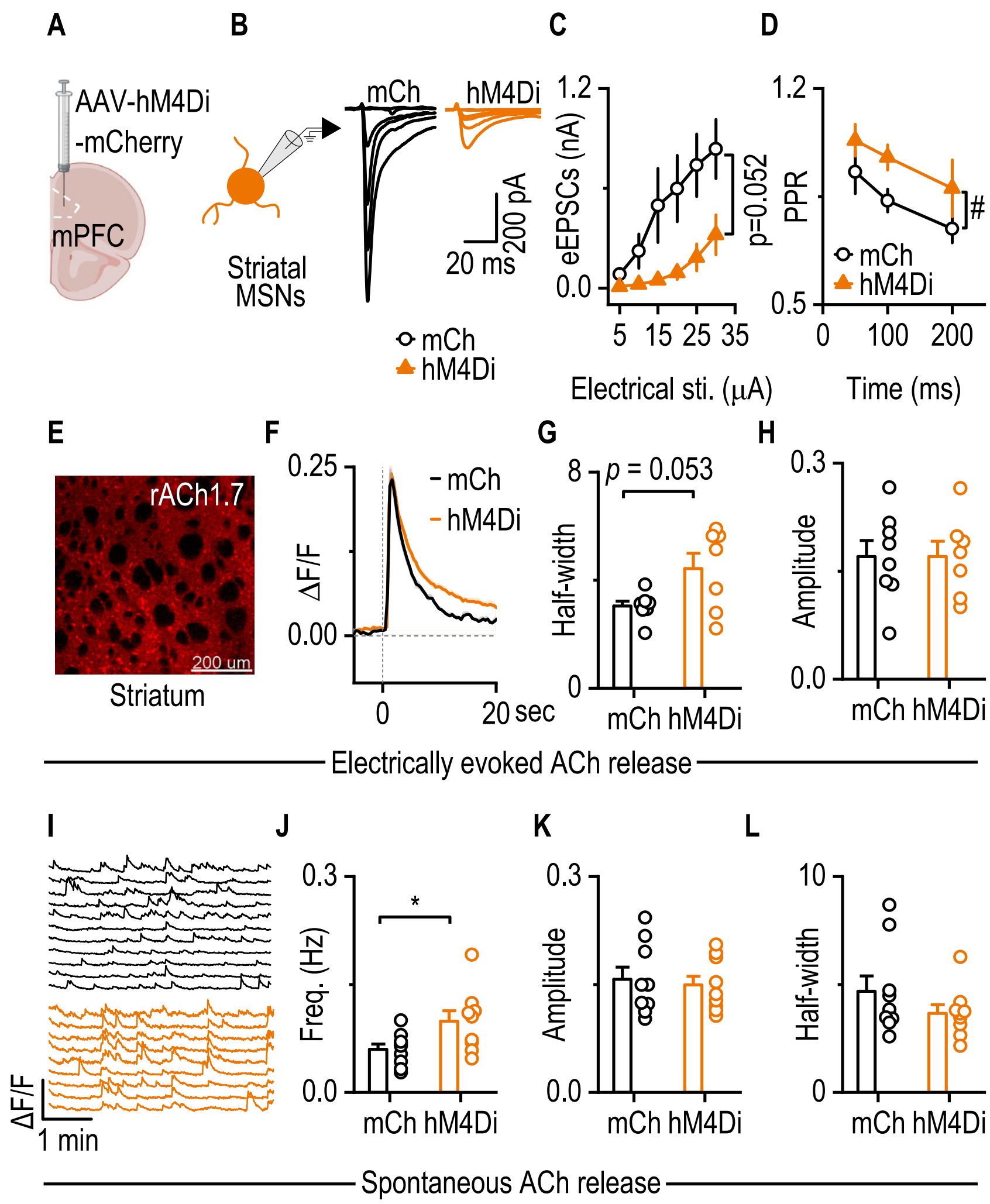

Supplementary Figure 13. Sustained chemogenetic inhibition of cortical neurons leads to decreased glutamatergic transmission and increased striatal ACh levels.

A, Schematic showing AAV-hM4Di-mCherry or AAV-mCherry infusion into the mPFC.

B, Representative trace of eEPSCs recorded from DMS MSNs in mCherry- and hM4Di-injected 5xFAD mice.

C, eEPSC amplitudes in DMS MSNs were smaller in hM4Di-injected 5xFAD mice than in mCherry-injected 5xFAD mice. Two-way RM ANOVA, $F_{(1,15)} = 4.435$, $p = 0.052$; n = 10 neurons from 4 mice (mCh); 7 neurons from 3 mice (hM4Di).

D, PPR in DMS MSNs was higher in hM4Di- than in mCherry-injected 5xFAD mice. Two-way RM ANOVA, $F_{(1,17)} = 5.213$, $p = 0.036$. $^{\#}p < 0.05$. n = 11 neurons from 4 mice (mCh); 8 neurons from 3 mice (hM4Di).

E, Representative image showing red ACh sensor (rACh1.7) expression in striatal slices from 5xFAD mice.

F, Sample traces of evoked striatal ACh release recorded from rACh1.7-expressing slices.

G and H, The half-width (G), but not the amplitude (H), of evoked ACh release was greater in hM4Di-injected mice than in mCherry-injected 5xFAD controls. Unpaired t-test with Welch's correction, $t_{7.130} = 2.309$, $p = 0.053$ for half-width; $t_{13} = 0.00147$, $p = 0.9989$ for amplitude. $^{*}p < 0.05$. n = 8 slices from 4 animals (G and H, mCh), 7 slices from 4 animals (G and H, hM4Di).

I, Sample traces of spontaneous striatal ACh release.

J-L, The frequency (J), but not the amplitude (K) or half-width (L), of spontaneous ACh release was significantly higher in hM4Di-injected 5xFAD mice compared to mCherry-injected 5xFAD mice. Unpaired t-test, (J), $t_{16} = 2.377$, $p = 0.0303$ for frequency; (K) $t_{16} = 0.3087$, $p = 0.7035$ for amplitude; (L) $U = 29$, $p = 0.3401$ for half-width. $^{*}p < 0.05$. n = 9 slices from 4 animals (mCh and hM4Di).

Data are mean ± SEM; tests were two-sided, with adjusted P values where applicable. Source data are provided as a Source Data file. Panel A was created in BioRender. Huang, Y. (2026) https://BioRender.com/kh5jchl.

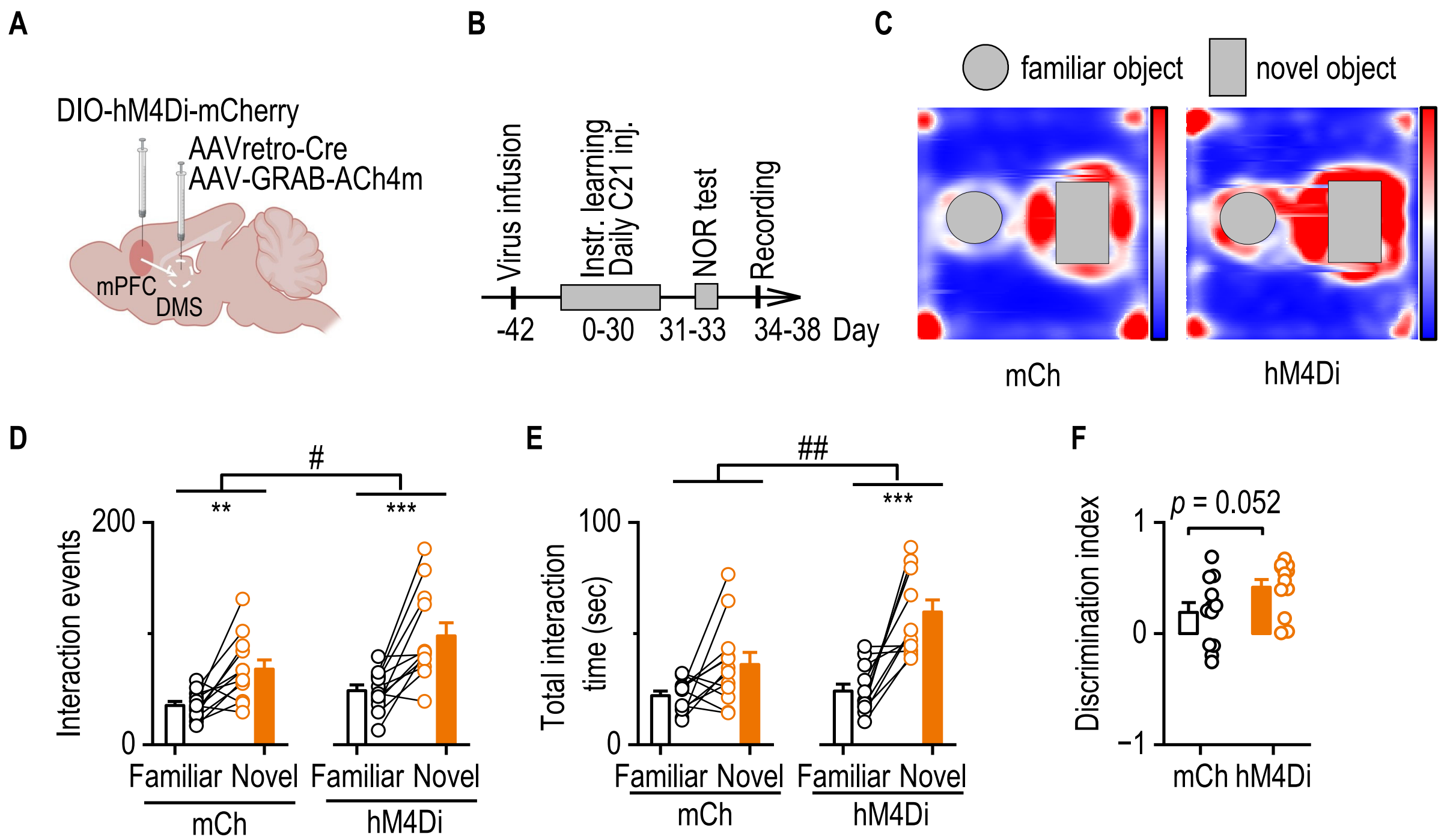

Supplementary Figure 14. Sustained inhibition of the mPFC to DMS circuit improved NOR performance in 5xFAD mice.

A, Schematic illustrating viral infusion strategy in 5xFAD mice. AAVretro-Cre and AAV-GRAB-ACh4m were infused into the DMS, and AAV-DIO-hM4Di-mCherry or AAV-DIO-mCherry into the mPFC.

B, Experimental timeline. The novel object recognition (NOR) test was performed after one month of sustained chemogenetic inhibition.

C, Heatmaps showing time spent interacting with novel and familiar objects during the 5-minute testing session.

D, Both hM4Di- and mCherry-injected 5xFAD mice showed more interaction events with the novel object than the familiar object. However, hM4Di-injected mice exhibited significantly more novel object interactions than mCherry controls. Two-way RM ANOVA, $F_{(1,22)} = 6.246$, $p = 0.0204$, $^{\#}p < 0.05$; Sidak's multiple comparisons post-hoc analysis, $^{*}p < 0.05$. n = 12 mice (mCh and hM4Di).

E, hM4Di-injected mice exhibited significantly longer interaction times. Two-way RM ANOVA, $F_{(1,22)} = 10.753$, $p = 0.0034$, $^{\#\#}p < 0.01$; Sidak's multiple comparisons post-hoc analysis, $^{*}p < 0.05$. n = 12 mice (mCh and hM4Di).

F, hM4Di-injected 5xFAD mice showed a higher discrimination index than mCherry-injected 5xFAD mice. Unpaired t-test, $t_{22} = -2.055$, $p = 0.0519$. n = 12 mice (mCh and hM4Di).

Data are mean ± SEM; tests were two-sided, with adjusted P values where applicable. Source data are provided as a Source Data file. Panel A was created in BioRender. Huang, Y. (2026) https://BioRender.com/kh5jchl.

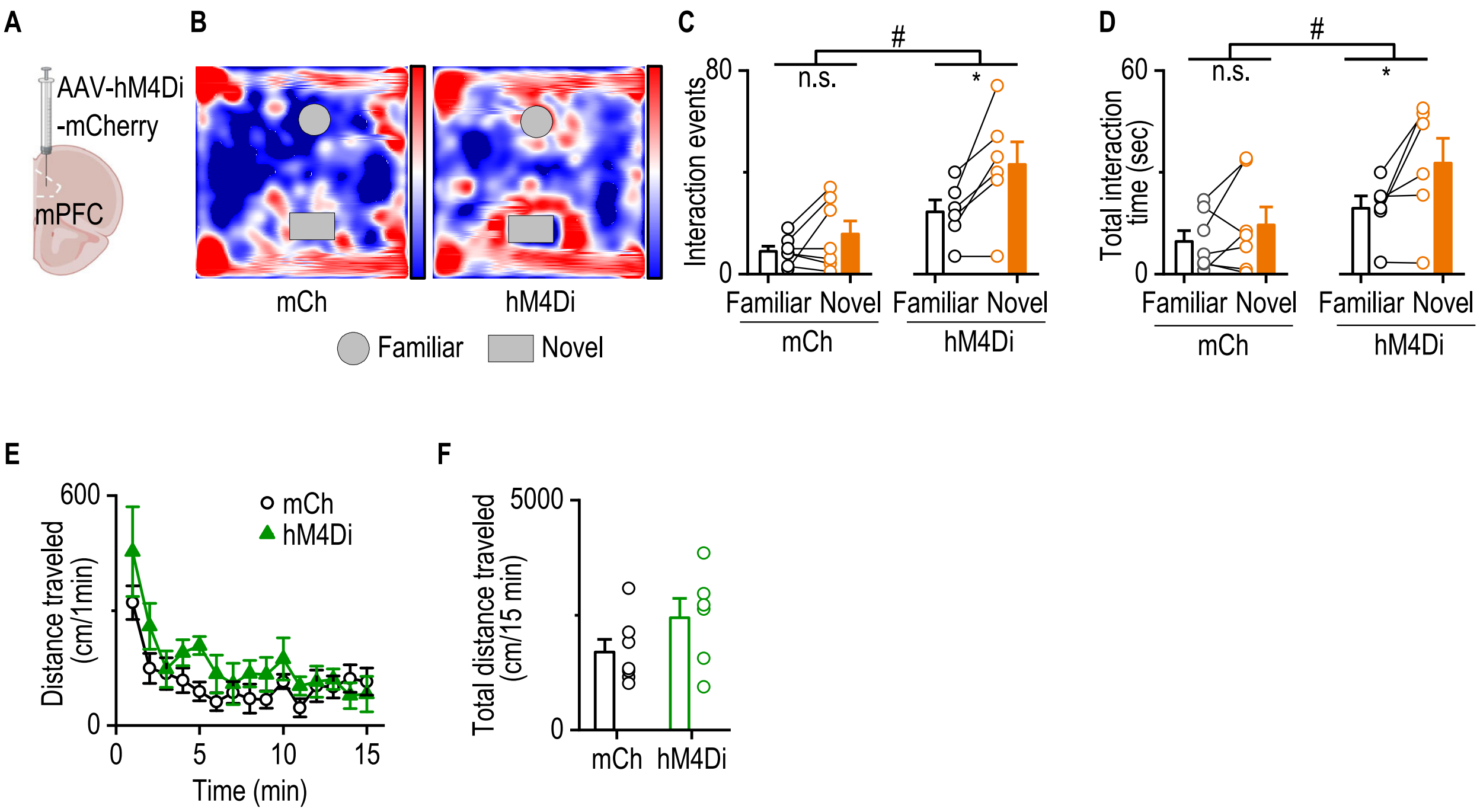

Supplementary Figure 15. Sustained inhibition of mPFC neurons improves cognitive function in 5xFAD mice without impairing locomotor activity.

A, Schematic showing AAV-hM4Di-mCherry or AAV-mCherry infusion in mPFC.

B, Heatmaps of animals that interacted with novel and familiar objects.

C, hM4Di-injected 5xFAD mice exhibited more interaction events with the novel object than controls in a 5-min session. Two-way RM ANOVA, $F_{(1,11)} = 9.460$, $p = 0.011$, $^{\#}p < 0.05$; Sidak's post-hoc, $^{*}p < 0.05$. n = 7 mice (mCh); 6 mice (hM4Di).

D, hM4Di-injected 5xFAD mice exhibited longer total interaction time with the novel object than controls in a 5-min session. Two-way RM ANOVA, $F_{(1,11)} = 4.906$, $p = 0.049$, $^{\#}p < 0.05$; post-hoc, $^{*}p < 0.05$. n = 7 mice (mCh); 6 mice (hM4Di).

E, F, hM4Di-injected and mCherry-injected 5xFAD mice exhibited similar spontaneous locomotor activity as measured by time-course (E, Two-way RM ANOVA, $F_{(1,11)} = 2.267$, $p = 0.1604$) and total distance traveled (F, unpaired t-test, $t_{11} = -1.506$, $p = 0.1604$). n = 7 (E and F, mCh), 6 (E and F, hM4Di).

Data are mean ± SEM; tests were two-sided, with adjusted P values where applicable. Source data are provided as a Source Data file. Panel A was created in BioRender. Huang, Y. (2026) https://BioRender.com/kh5jchl.
