# Supplementary material for: Early-Stage Corticostriatal Hyperactivity Impairs Cognitive Flexibility Alongside Striatal Cholinergic Dysfunction in an Alzheimer’s Disease Model
Source: Nat Commun. 2026 Jun 26;17:8226. doi: 10.1038/s41467-026-74817-z (PMC13462761; doi:10.1038/s41467-026-74817-z)
Supplement: Supplementary file 2 [file 41467_2026_74817_MOESM2_ESM.pdf]

nature portfolio

Corresponding author(s): Jun Wang

Last updated by author(s): May 18, 2026

Reporting Summary

Nature Portfolio wishes to improve the reproducibility of the work that we publish. This form provides structure for consistency and transparency in reporting. For further information on Nature Portfolio policies, see our Editorial Policies and the Editorial Policy Checklist.

Statistics

For all statistical analyses, confirm that the following items are present in the figure legend, table legend, main text, or Methods section.

n/a	Confirmed	
☐	☒	The exact sample size (*n*) for each experimental group/condition, given as a discrete number and unit of measurement
☐	☒	A statement on whether measurements were taken from distinct samples or whether the same sample was measured repeatedly
☐	☒	The statistical test(s) used AND whether they are one- or two-sided<br>*Only common tests should be described solely by name; describe more complex techniques in the Methods section.*
☒	☐	A description of all covariates tested
☐	☒	A description of any assumptions or corrections, such as tests of normality and adjustment for multiple comparisons
☐	☒	A full description of the statistical parameters including central tendency (e.g. means) or other basic estimates (e.g. regression coefficient) AND variation (e.g. standard deviation) or associated estimates of uncertainty (e.g. confidence intervals)
☐	☒	For null hypothesis testing, the test statistic (e.g. *F*, *t*, *r*) with confidence intervals, effect sizes, degrees of freedom and *P* value noted<br>*Give P values as exact values whenever suitable.*
☒	☐	For Bayesian analysis, information on the choice of priors and Markov chain Monte Carlo settings
☒	☐	For hierarchical and complex designs, identification of the appropriate level for tests and full reporting of outcomes
☒	☐	Estimates of effect sizes (e.g. Cohen's *d*, Pearson's *r*), indicating how they were calculated

Our web collection on statistics for biologists contains articles on many of the points above.

Software and code

Policy information about availability of computer code

Data collection	Olympus FluoView FV3000 software was used for confocal imaging.<br>Med Associates software was used for operant behavioral task control and data acquisition.<br>Ethovision XT 15 (Noldus) was used for video tracking and behavioral data collection in the Barnes maze and Novel Object Recognition test.<br>Activity Monitor (MED Associates) was used for open-field locomotor activity tracking.<br>Clampex 10.6 (Molecular Devices) was used for electrophysiological recording acquisition
Data analysis	GraphPad Prism 10, SigmaPlot 12.5, and IBM SPSS Statistics GradPack 29 – used for statistical analysis<br>MATLAB 2025a – used for imaging signal analysis (e.g., ΔF/F, z-score, half-width); custom MATLAB analysis code is available.<br>ImageJ – used for quantification of immunostaining and fluorescent signal intensity<br>Imaris 8.3.1 (Bitplane) – used for 3D reconstruction and cell quantification in confocal images<br><br>Custom MATLAB analysis code used for live confocal imaging data analysis were written by the authors based on standard analysis procedures and have been deposited in a public GitHub repository. The Code Availability section provides the GitHub link and access information. No newly developed software package or algorithm was generated for this study.

For manuscripts utilizing custom algorithms or software that are central to the research but not yet described in published literature, software must be made available to editors and reviewers. We strongly encourage code deposition in a community repository (e.g. GitHub). See the Nature Portfolio guidelines for submitting code & software for further information.

Data

Policy information about availability of data

All manuscripts must include a data availability statement. This statement should provide the following information, where applicable:

- Accession codes, unique identifiers, or web links for publicly available datasets
- A description of any restrictions on data availability
- For clinical datasets or third party data, please ensure that the statement adheres to our policy

Source data underlying all main and supplementary figures, including numerical values used to generate graphs and statistical analyses, are provided with this paper as a Source Data file.

Research involving human participants, their data, or biological material

Policy information about studies with human participants or human data. See also policy information about sex, gender (identity/presentation), and sexual orientation and race, ethnicity and racism.

Reporting on sex and gender	Not applicable. This study did not involve human participants.
Reporting on race, ethnicity, or other socially relevant groupings	Not applicable. This study did not involve human participants.
Population characteristics	Not applicable. This study involved animal models, not human participants.
Recruitment	Not applicable. This study used laboratory animals and did not involve participant recruitment.
Ethics oversight	Not applicable. This study did not involve human participants.

Note that full information on the approval of the study protocol must also be provided in the manuscript.

Field-specific reporting

Please select the one below that is the best fit for your research. If you are not sure, read the appropriate sections before making your selection.

- [x] Life sciences
- [ ] Behavioural & social sciences
- [ ] Ecological, evolutionary & environmental sciences

For a reference copy of the document with all sections, see nature.com/documents/nr-reporting-summary-flat.pdf

Life sciences study design

All studies must disclose on these points even when the disclosure is negative.

Sample size	No formal statistical method was used to predetermine sample size. Sample sizes were chosen based on prior published studies and standard practice in the field to ensure sufficient power to detect biologically meaningful effects. Sample size per group is specified in figure legends.
Data exclusions	Data were excluded only when pre-established technical criteria were not met. Exclusions included: (1) poor tissue quality or damage during slicing that impaired structural integrity; (2) unstable electrophysiological recordings (e.g., high series resistance or rundown); (3) imaging sessions with motion artifacts or poor signal-to-noise ratio; and (4) behavioral sessions in which animals did not engage with the task (e.g., no movement or failure to complete trials). All exclusion criteria were defined in advance and applied consistently across experiments.
Replication	All key findings were replicated across at least two independent cohorts of animals and/or experimental sessions. Specific replication details, including biological and technical replicates, are reported in the figure legends and Methods.
Randomization	Animals were randomly assigned to experimental groups based on genotype and treatment conditions. When possible, littermates were distributed across groups to minimize genetic and environmental bias.
Blinding	Blinding was performed during data collection and analysis. Investigators were blinded to genotype and treatment conditions during behavioral testing, imaging analysis, and electrophysiological recordings.

Reporting for specific materials, systems and methods

We require information from authors about some types of materials, experimental systems and methods used in many studies. Here, indicate whether each material, system or method listed is relevant to your study. If you are not sure if a list item applies to your research, read the appropriate section before selecting a response.

Materials & experimental systems

n/a	Involved in the study	
☐	☒	Antibodies
☒	☐	Eukaryotic cell lines
☒	☐	Palaeontology and archaeology
☐	☒	Animals and other organisms
☒	☐	Clinical data
☒	☐	Dual use research of concern
☒	☐	Plants

Methods

n/a	Involved in the study	
☒	☐	ChIP-seq
☒	☐	Flow cytometry
☒	☐	MRI-based neuroimaging

Antibodies

Antibodies used

Rabbit anti-Aβ (Invitrogen, catalog #71-5800, LOT YB369857, 1:300 dilution)
Rabbit anti-cFos (EMD-Millipore, catalog #ABE457, LOT 3988907, 1:2000 dilution)
Alexa Fluor 647-conjugated goat anti-rabbit IgG (H+L) (Invitrogen, catalog #A21245, LOT 2291604, 1:500 dilution)
Biotinylated donkey anti-rabbit IgG (Jackson ImmunoResearch, catalog #711-065-152, LOT 165233, 1:500 dilution)
Streptavidin-conjugated Alexa Fluor 647 (Thermo Fisher, catalog #21374, LOT 2291604, 1:1000 dilution)

Validation

All antibodies used are commercially available and have been validated by the manufacturers for immunofluorescence in mouse tissue. The Aβ and c-Fos antibodies have been widely cited in peer-reviewed literature and are known to yield specific labeling consistent with expected expression patterns. Secondary antibodies were used in combination with controls to confirm signal specificity. Detailed antibody information and experimental conditions are provided in the Methods section of the manuscript.

Animals and other research organisms

Policy information about studies involving animals; ARRIVE guidelines recommended for reporting animal research, and Sex and Gender in Research

Laboratory animals

ChAT-eGFP (stock # 007902), 5xFAD (stock # 034848), ArcTRAP (#021881), Ai14- tdTomato (#007914), and Drd1a-tdTomato (stock # 016204) mice were purchased from the Jackson Laboratory. D1-Cre mice were obtained from Mutant Mouse Regional Resource Centers. All mice were backcrossed onto a C57BL/6J background. ChAT-eGFP mice were crossed with 5xFAD to generate a 5xFAD;ChAT-eGFP line. 5xFAD;ChAT-eGFP mice were crossed with D1-Cre to generate D1-Cre;ChAT-eGFP;5xFAD line. D1- tdTomato mice were crossed with 5xFAD to generate a 5xFAD;D1tdT mouse line. ArcTRAP mice were crossed with Ai14 to generate Arc;Ai14 mice. Arc;Ai14 mice were crossed with 5xFAD mice to generate triple transgenic Arc;Ai14;5xFAD mice. Mice were group-housed at 23°C with a 12-h light:dark cycle (lights on at 11:00 pm). Relative humidity limits are set between 30% and 70%. Food and water were provided ad libitum.

Wild animals

This study did not involve wild animals.

Reporting on sex

Both male and female mice were used, and sexes were balanced across experimental groups. Sex was not a primary experimental variable, and no sex-specific analyses were performed.

Field-collected samples

This study did not involve field-collected samples.

Ethics oversight

All animal care procedures and experimental protocols were approved by the Texas A&M University Institutional Animal Care and Use Committee and were performed in accordance with the National Research Council Guide for the Care and Use of Laboratory Animals.

Note that full information on the approval of the study protocol must also be provided in the manuscript.

Plants

Seed stocks

Not applicable. This study did not involve any plant material.

Novel plant genotypes

Not applicable. This study did not generate or use any plant genotypes.

Authentication

Not applicable. This study did not involve plant genotypes requiring authentication.
