# Supplementary material for: Early-Stage Corticostriatal Hyperactivity Impairs Cognitive Flexibility Alongside Striatal Cholinergic Dysfunction in an Alzheimer’s Disease Model
Source: Nat Commun. 2026 Jun 26;17:8226. doi: 10.1038/s41467-026-74817-z (PMC13462761; doi:10.1038/s41467-026-74817-z)
Supplement: Supplementary file 3 [file 41467_2026_74817_MOESM3_ESM.pdf]

nature portfolio

Peer Review File

Early-Stage Corticostriatal Hyperactivity Impairs Cognitive Flexibility Alongside Striatal Cholinergic Dysfunction in an Alzheimer's Disease Model

Corresponding Author: Dr Jun Wang

This file contains all reviewer reports in order by version, followed by all author rebuttals in order by version.

Version 0:

Reviewer comments:

Reviewer #1

(Remarks to the Author)
In this manuscript, the Authors study the 5xFAD mouse, a mouse model of Alzheimer's disease, using electrophysiology and behavioural techniques, combined with chemogenetic and optogenetic stimulation, to investigate the circuits between the medial prefrontal cortex (mPFC) and dorsomedial striatum (DMS) and their relation with deficits in reversal learning. In particular, they demonstrate with ex-vivo recordings that mPFC neurons are hyperactive in 4-month-old 5XFAD mice, causing increased activity of striatal dMSN neurons, which are directly innervated by mPFC neurons. The hyperactivity of dMSNs, in turn, excessively inhibits cholinergic neurons in the striatum (CINs), which then release less acetylcholine (ACh). Associated with this alteration in the neural circuit, 5XFAD mice show deficits in cognitive flexibility during behavioural tests, failing reversal learning tasks. Using the chemogenetic approach, the authors selectively inhibit the mPFC-Striatum circuit, demonstrating that ACh release in the striatum is restored, cognitive deficits are recovered, and the accumulation of Aβ plaques in the mPFC decreases. The methodology applied to this work is pretty solid and most results are convincing and well explained. Following are some comments and suggestions to be applied to improve the quality and readability of the manuscript.

Major comments:
1. Generic comment: The different stats tests are mentioned in the Figure Legends but the results of the tests (p and F values) are currently in the text; to facilitate the reading I would recommend moving everything in the legends, to be able to more easily follow the figures.
2. Line 98: are the stats mentioned based on paired tests? This is not clear from the figure legends.
3. Fig.2:
a. IF panels (B,C and D,E): the background fluorescence appears higher in 5xFAD mice in the initial condition. Are these IFs taken with same laser settings for WT and AD mice? I would overall suggest changing the representative images as these do not allow to appreciate differences (of lack there of) in Arc expression. This stands for both panels 2B,C and for 2D,E. Perhaps DAPI or higher magnification images (as in 2B) would also help, as performed for the dentate gyrus
b. Statistical analysis: according to the authors's hypothesis, mPFC or DMS neurons become hyperactive after the reversal phase of the test but are normally active during the initial phase. The analysis shown in Figs 2B,C and 2D,E confronts control vs 5xFAD neurons but the confrontation of 5xFAD initial vs reversal is missing. A 2-way ANOVA might be a more appropriate test to confront all 4 groups (WT and 5xFAD, initial and reversal phase) for both the mPFC and DMS.
4. Fig. 3: rappresentative traces in Fig.3C should be changed, as the increase in frequency is not evident in this scaling. Please add also higher-scaling traces (scaled for both amplitude and time) to be able to appreciate differences in amplitude and frequency. Also, the 'burst-like' activity of sEPSCs in AD mice is highly atypical, and should be described further in the text. Was this a frequent event in all patches? Was the analysis of these huge (nA) events included in the overall analysis shown in panels D and E? I would expect the mean amplitude of sEPSCs in AD mice to be much higher that what shown in D, given that many events, even outside the 'bursts', reach the 500pA-1nA scale.
5. Fig. 4C,D: please add representative traces. Overall, the results in Fig. 4D are puzzling: if mPFC glutamatergic neurons are hyperexcitable, an increase in the frequency of sEPSCs in MSNs should be seen, which is not the case from what is shown in the plot. It is probable that these MSN neurons recorded in the striatum are not directly innervated by the mPFC, resulting in misleading information (see lines 206-208, that are contradicted further on). This issue is important since the authors claim that the hyperactivity of the dMSNs is directly driven by the mPFC (line 210), and they clearly demonstrate with

optogenetic stimulation in panel Fig.4F-H. Thus, I recommend removing the sEPSC results from the paper, to be substituted by experiments in mPFC-retrogradely labelled MSN neurons (or in experiments of pre-post optogenetic mPFC stimulation)
6. In Fig. 6B, the claim that Aβ levels are decreased is not very convincing, at least based on the representative figure. Either fluorescence intensity or size of individual plaques should also be analyzed. At this age, does this model also show an accumulation of Aβ plaques in the striatum? If so, I suggest analyzing the reduction of Aβ plaques in this area as well.
7. In addition, do you see a different rate of decline in Aβ between male and female mice? Female 5xFAD mice show a significantly greater Aβ plaque burden compared to males, particularly in the cortex and striatum. The authors might want to discriminate between the two sexes.
8. Line 463: Males and females were pooled in the experiments. Before doing so, did the authors ensure that there were no significant differences in the experimental results due to sex?
Minor comments:
1. Fig. 3G and 5G: example traces are missing
2. Fig 4E and 4I: example traces are missing
3. Given the increase excitability of mPFC neurons, authors are requested to provide a more detailed analysis of the current-clamp recordings. It would also be advisable to analyse their membrane properties, such as resting membrane potential, the input resistance, the rheobase, the firing threshold ecc. This might provide more indications as to the hyperexcitability of these cells.
4. Fig. 2H-I. From the merge, in 5XFAD group the co-labelling between co-expression of tdTomato and c-Fos in mPFC and DMS neurons is not clearly visible. Please change the representative image or add higher magnification images.
5. in Fig. 3C, change AD with 5xFAD for consistency, and correct uA to µA in panel 3F
6. in Fig 5K: what does the Y scale indicate? fluorescence intensity ΔF/F?
7. Figure Suppl 5F. Sample trace in EEG recordings showing spikes before and after pilocarpine i.p. injection is form WT or 5XFAD mice? Anyway, it is advisable to show the trace in both groups for comparison.
8. Authors are requested to provide more details on the regions they recorded. For example, specify in which subregions of the medial PFC the electrophysiology recordings were performed, or even more specifically, in which layer?
9. Electrophysiology methods: Please specify which antagonists were used for the ex-vivo electrophysiology recordings and at what concentrations. Add duration of optical or electrical stimulation
10. Please specify more clearly in the text at what age the experiments are performed. From this point of view, the text is a bit confusing.

Reviewer #2

(Remarks to the Author)
This is a conceptually solid and potentially high-impact study addressing how corticostriatal hyperactivity contributes to early cognitive inflexibility in the 5xFAD model of Alzheimer's disease. The authors propose that amyloid-β–induced hyperactivity within the medial prefrontal cortex–to–dorsomedial striatum circuit enhances direct pathway medium spiny neuron inhibition of cholinergic interneurons, thereby reducing striatal acetylcholine release and impairing reversal learning. Sustained chemogenetic inhibition of this projection reportedly decreases cortical amyloid-β accumulation, normalizes glutamatergic transmission, restores cholinergic signaling, and rescues cognitive flexibility.
However, the following points merit clarification or strengthening before publication.

Although the manuscript describes a prefeeding procedure before devaluation testing ("one-hour free access to 40 randomly assigned purified or grain pellets"), this manipulation by itself does not establish whether satiety and reinforcer valuation are equivalent across genotypes. No data are reported on the amount consumed, the rate of consumption, or the efficacy of satiety induction. To exclude motivational or palatability confounds, the authors should quantify prefeeding consumption and/or perform a brief post-prefeed preference or progressive-ratio test to confirm outcome-specific value shifts in both groups. Because motivation and reinforcer value directly influence devaluation performance, the authors should measure satiety effectiveness to rule out behavioral confounds.

ArcTRAP recombination intergrates Arc expression over several hours, meaning that labled neurons likely reflect activity occurring around the behavioral session rather than exclusively within it. Acknowledge that labeled neurons may partially reflect home-cage or post-session activation. Authors can refer to temporally precise methods (CaMPARI, Cal-Light) to validate epoch specificity.

Targeted morphological analyses, such as quantification of dendritic spine density and classification of spine subtypes in behaviorally engaged ensembles, would complement the glutamatergic synaptic physiology data, providing complementary structural evidence for altered connectivity in the activated neuronal population.

The authors should clarify how statistical independence was defined for all slice and cellular datasets. If multiple cells or slices were collected from the same animal, the effective biological sample size (n) must be defined as the number of animals, not cells, to avoid pseudoreplication. Mixed-effects or nested models (cells nested within animals) should be used to account for within-animal variance. Alternatively, results should be re-expressed as animal-level means and reanalyzed accordingly. This is particularly important for this review's eyes, especially in experiments with transgene(s). E.g., to ensure that genotype-dependent differences in IPSC amplitude or input–output slope reflect unitary synaptic properties rather than recruitment differences, the authors should quantify Chrimson-tdTomato expression, infection fraction, and light-evoked spike probability versus light power in dMSNs. This control is essential to confirm that an equivalent optical drive was achieved.

Reviewer #3

(Remarks to the Author)
This study reports a deficit in instrumental, but not spatial, reversal learning in 4-month old male and female 5xFAD mice This deficit associates with elevated neuronal reactivation in two regions, the medial prefrontal cortex (mPFC) and the dorsomedial striatum (DSM), involved in goal-directed behaviors. In-depth dissection of neural and circuits alterations underpinning behavioral abnormalities then reveals that the mPFC-to-DMS circuit is hyperactive in 5xFAD mice, and that this hyperactivity triggers alterations in corticostriatal and cholinergic signaling at the origin of behavioral inflexibility.
This is an ambitious study based on behavioral assays and cutting-edge neurobiological methods whose conclusions need, however, to be confirmed considering the potential biases of behavioral protocols and the insufficient details provided in description of the experimental design.

Main points

Behavior
1. The reversal protocol is questionable because the mice undergo a devaluation test that alters the outcome value just before their reversal training, potentially decreasing their motivation to obtain food. Furthermore, reversal training is performed immediately in the most demanding RR20 condition, as the mice have a 0.05 probability of receiving a reward. Therefore, it is difficult to determine whether the reduction in lever presses during reversal training and the subsequent absence of post-reversal devaluation which are both selectively altered in the 5xFAD mice can be ascribed to a decrease in motivation or a deficit in behavioral flexibility. It is thus imperative to test the mice using a procedure where contingency training is followed by contingency reversal, without an intermediate post-training devaluation test.

2. The Barnes test, used to demonstrate that spatial learning and reversal are intact in 4-month-old 5xFAD mice, does not involve food rewards, is less effortful, and much simpler, in terms of amount of information to process, than reversal training performed under RR20 conditions. It has been suggested that the medial prefrontal cortex (mPFC) is involved in reversal learning only when the task becomes more complex and demands a higher level of attention (Izquierdo et al., Neuroscience, 2017,45:12–2). The use of an alternative task (e.g., a radial maze with different subsets of baited arms during training and reversal) is needed to determine whether the reversal deficit observed in 5xFAD mice is specific to goal-directed (and not spatial) tasks, or specific to complex tasks, independent of goal-directed or spatial contingencies.

mPFC subregions regions
Numerous data in humans and rodents indicate a functional specificity of subregions of the medial prefrontal cortex, with differential involvement of the prelimbic, infralimbic, and orbitofrontal cortices in various functions, including reward and punishment, extinction, working memory, attention, value-based decision-making, goal-oriented behavior, and reversal learning. This aspect is not considered in the present study, despite its importance for refining the interpretation of the data. According to the mouse brain atlas by Paxinos and Franklin, the reported stereotaxic coordinates of the virus injection site (AP: 1.94 mm, ML: ± 0.35 mm, DV: -2.20 mm) should target the PL and IL regions of the anterior part of the medial prefrontal cortex, suggesting that neurobiological measurements were performed in these regions. The authors should specify the location of their observations and manipulations and adapt the interpretation of their data accordingly.

Supplementary data
Paradoxically, this section includes more experiments than the main part, with some experiments being as much crucial as those of the main part. In particular, findinggs reported in Suppl. Figures 6,7,8,9,10,11) provide evidence that correcting hyperexcitability in abnormally activated regions and circuits of 5xFAD mice is sufficient to rescue behavioral and neuronal alterations, including the reduction of Abeta levels and the normalization of acetylcholine levels. This paradox definitely raises the question of the reorganization of the manuscript. One option would be to present in the main part the "dysfunctions" and their "rescue" in the same figures, and to remove in the supplementary data the experiments (pilocarpine, object novelty, investigations performed at 12 months) which are indirectly related to the central topic of the study (identification of an early deficit in 4-month old 5xFAD mice

Methods
1. Experiments design
Experiments expected to link behavioral impairments to neurobiological alterations (e.g. electrophysiology) were carried out at age points which do not exactly coincide. Reversal learning: 3, 6, 12 months; Electrophysiology: 4 and 5 months; Pathway studies: 4 and 12 months; Seizures and EEG: not indicated. Maybe a diagram or a timeline specifying the ages of mice at each experimental phase might help to distinguish between neurobiological causal alterations and their long term consequences.

2. Administration of 4-OHT with isoflurane
It is reported that "The 4-OHT was administered immediately following either the completion of the middle two initial RR20 sessions or after the reversal RR20 sessions. Mice were first introduced to an isoflurane chamber immediately after the operant session, and 4-OHT was injected i.p. The use of isoflurane is to reduce the pain and discomfort due to the 4-OHT/oil mix injection."
This sentence should be rewritten. It is unclear whether mice were anesthetized immediately after, or during, or before behavioral assessment thereby preventing to estimate anesthesia interference with performance or memory. Moreover, the justification for administering isofluorane before 4-OHT injections sounds a bit inconsistent with the fact that the 30-day i.p.

injections of Compound 21 were administered in awake mice.

3. Off-target potential effects of Compound 21 injections
There is evidence that, at high doses, Compound-21 also binds to endogenous ACh receptors with consequent increase in available and detectable acetylcholine (Jendrila M et al, Sci Rep 201, 9:4522). The range of the injected dose should be verified to discard this off-target effect.

4. Hippocampal data
Images provided in Suppl Fig3 show that trapped dentate gyrus neurons do not differ between WT and 5xFAD in either initial or reversal learning This observation is in contrast with the report that hippocampal neurogenesis contribute to cognitive flexibility (Anacker & Hen, 2017 Nat Rev Neurosci 18, 335–346. This study should be cited and commented.

5.Statistical analyses
Two-way ANOVA with genotype and sessions comparing RR20 performance between contingency training and contingency reversal, or comparing Val vs Dev scores between post-training and post-reversal devaluation tests the different ages mice were tested would have assessed more accurately inter-strain differences in behavior.

Theoretical framework
The authors alternately interpret the behavioral deficit in 5xFAD mice as an alteration in goal-oriented behavior or an alteration in cognitive flexibility. While both of these executive functions require the modification of previously learned responses, goal-oriented systems involve a specific sensitivity to the value of the outcome, which generates the action necessary to reach it. It is worth noting that outcome devaluation is considered one of the best methods for identifying goal-oriented behavior in rodents (Pierce-Messick et al., Nat Protoc. 2025, 20:518-538). Thus, the fact that 4-month old WT and 5xFAD mice exhibit significantly less lever presses for devalued outcomes (Dev) than for valued outcomes (Val) in the post-training devaluation test reveals that the mutant mice do exhibit goal-oriented behavior. This point should be clearly stated in the discussion. Indeed, the fact that they do not show devaluation after reversal training suggests the existence of experimental factors that overall abolish their prior ability to devaluate.

Reviewer #4

(Remarks to the Author)
Cognitive flexibility deficits emerge early in Alzheimer’s disease (AD), yet their circuit basis is unclear. In 5xFAD mice, the authors found early reversal learning impairments linked to hyperactivity in the medial prefrontal cortex (mPFC) and dorsomedial striatum (DMS). mPFC neurons and DMS direct pathway neurons were hyperexcitable, increasing inhibition of cholinergic interneurons and reducing striatal acetylcholine release. Chemogenetic suppression of the mPFC–DMS circuit normalized neurotransmission, lowered cortical Aβ, and rescued learning deficits, revealing a hyperactive corticostriatal pathway as a driver of cognitive inflexibility in early AD. Experiments were well executed. The manuscript is well written and highlights interesting features in excitability underlying changes in cognitive flexibility in the 5xFAD mouse at early stages of disease progression. However, although the phenomenon is very interesting, the manuscript lacks an investigation of the molecular bases underlying changes in excitability that one would have expected for a thorough investigation of the phenomenon.

Reviewer #5

(Remarks to the Author)
I co-reviewed this manuscript with one of the reviewers who provided the listed reports. This is part of the Nature Communications initiative to facilitate training in peer review and to provide appropriate recognition for Early Career Researchers who co-review manuscripts.

Version 1:

Reviewer comments:

Reviewer #1

(Remarks to the Author)
This is an ambitious and potentially important research paper. The authors combine behavior, activity-dependent tagging, ex vivo electrophysiology, acetylcholine sensor imaging, and chemogenetic circuit inhibition to argue that early mPFC→DMS hyperactivity in 5xFAD mice suppresses striatal cholinergic function and drives early cognitive inflexibility. The study is conceptually strong and the overall model is interesting, especially the idea that executive dysfunction precedes overt spatial memory deficits and is linked to corticostriatal rather than hippocampal dysfunction at this stage. However, several central claims are currently stated more strongly than the data justify, and the manuscript would benefit from tighter statistical/reporting standards and a more careful treatment of causality.

The abstract is clear and impactful, but the causal language is too strong in its current form. The data support a model in which pathological corticostriatal hyperactivity is associated with, and likely contributes to, cholinergic dysfunction and reversal-learning deficits. However, the manuscript does not yet directly demonstrate that the dMSN→CIN→ACh node is

itself necessary or sufficient for the behavioral phenotype. I would recommend softening phrases such as “disrupts cholinergic signaling and drives early cognitive inflexibility,” unless the authors add a more direct mechanistic rescue at the CIN/ACh level.

Figure 1
My main concern is interpretive precision. Figure 1 supports an early deficit in updating action-outcome contingencies, but the authors should be careful not to overstate this as a general executive dysfunction phenotype on the basis of one reversal paradigm alone. In addition, the main figure and legend/reporting should make it absolutely transparent how sample sizes were defined for each behavioral panel and whether sex was balanced across cohorts. Finally, because the entire paper builds on this behavioral dissociation, effect sizes and confidence intervals would be especially useful here.

Figure 2

The ArcTRAP/c-Fos experiments are interesting and add an ensemble-level dimension to the story. The finding that reversal learning in 5xFAD mice is associated with increased activation/reactivation in mPFC and DMS is consistent with the circuit model proposed by the authors.
That said, this figure should be interpreted more cautiously. As the authors themselves later acknowledge, ArcTRAP integrates activity over relatively broad time windows and cannot resolve specific task epochs. Therefore, the data support abnormal recruitment/reactivation associated with reversal learning, but they do not prove that the same maladaptive ensemble dynamics are the mechanism of behavioral inflexibility. I would recommend softening the wording around “excessive reactivation” unless the authors can provide temporally sharper evidence. Clearer reporting of biological n at the animal level would also help.

Figure 3

This figure is convincing in showing that mPFC pyramidal neurons in young 5xFAD mice are hyperexcitable and receive increased excitatory input. The combination of intrinsic excitability, spontaneous EPSCs, evoked EPSCs, and AMPA/NMDA ratio gives a coherent picture of cortical hyperexcitability at an early stage.
The main issue here is statistical and inferential. For all electrophysiological analyses, the authors should clearly state whether statistics were performed at the neuron level or animal level, and ideally reanalyze key datasets using mixed-effects models that account for cells nested within animals. Without that, the strength of the conclusion is harder to judge. I also think the discussion should more clearly distinguish between evidence for increased excitatory transmission and the more speculative suggestion of impaired glutamate clearance/spillover.

Figure 4

This is one of the most interesting figures in the paper. The selective enhancement of mPFC input onto dMSNs, but not iMSNs, provides real specificity and strengthens the mechanistic logic of the model. The persistence of this abnormality at 12 months is also notable.
Still, several points need clarification. First, the figure would benefit from more explicit reporting of viral targeting and exclusion criteria in the main text or figure legend. Second, the authors should be cautious with the language “selectively hyperactive,” because what is shown directly is enhanced excitability/synaptic drive under the recording conditions, not necessarily in vivo firing selectivity during behavior. Third, the seizure and EEG data are useful as supportive evidence of broader network hyperexcitability, but they are relatively indirect with respect to the specific mPFC→dMSN pathway and should not be treated as pathway-specific confirmation.

Figure 5

This is the key mechanistic bridge in the manuscript. The evidence that dMSN-->CIN inhibition is enhanced, CIN spontaneous firing is reduced, and spontaneous ACh events are decreased is internally coherent and fits the proposed model well. This is an important strength of the paper.
However, this figure still stops short of demonstrating that reduced CIN/ACh signaling is the necessary mediator of the behavioral phenotype. The manuscript would be substantially strengthened by a direct rescue experiment at this node, for example restoring CIN activity or cholinergic tone and testing whether reversal learning improves. Without such an experiment, the safest conclusion is that altered dMSN→CIN signaling is a plausible and well-supported intermediate mechanism, not yet a definitively established causal bottleneck.

Figure 6

The long-term chemogenetic inhibition experiment is important because it adds an intervention dimension and links circuit activity to amyloid burden, synaptic transmission, and striatal ACh output. The result that projection-specific mPFC→DMS inhibition reduces cortical plaque burden and normalizes downstream readouts is potentially very significant.
At the same time, this figure also illustrates the manuscript’s main interpretive problem: the intervention changes many variables at once. Because chronic hM4Di/C21 manipulation affects Aβ, cortical transmission, striatal transmission, and ACh, it is difficult to assign behavioral rescue specifically to cholinergic normalization. The authors should therefore reduce the specificity of the mechanistic claims, unless they add a more targeted experiment downstream. This section also needs especially careful reporting of viral spread, projection specificity, treatment controls, and how long after the last C21 injection the measurements were made.

Figure 7

The behavioral rescue is compelling and gives the paper translational relevance. The fact that sustained mPFC→DMS inhibition restores reversal-learning performance in 5xFAD mice is an important result and fits well with the overall circuit model. The additional object-recognition improvement is also interesting.
My concern is again one of mechanistic specificity. Because the same chronic manipulation also reduced cortical Aβ accumulation and altered excitatory transmission, Figure 7 cannot by itself show that reversal-learning rescue occurred specifically because cholinergic signaling was restored. The figure therefore supports a strong causal role for the upstream circuit, but not yet the full downstream pathway claimed in the manuscript. This distinction should be reflected in the wording of the Results and Discussion.

This is a strong and potentially impactful manuscript with a compelling central idea and a rich dataset. The behavioral dissociation is interesting, the pathway specificity in Figure 4 is a major strength, and the CIN/ACh data are mechanistically suggestive and important. However, the current version overstates the level of causal proof, and the statistical/reporting framework needs to be tightened substantially. With major revision, and ideally with a direct test of the CIN/ACh node, this study could become suitable for publication.

Reviewer #2

(Remarks to the Author)
The authors have adequately addressed the points raised in my previous revision. The manuscript has improved accordingly and I have no further comments or concerns.

Reviewer #3

(Remarks to the Author)
The authors have satisfactorily addressed the 10 points raised by my co-reviewer and myself.
We particularly appreciated the additional behavioral experiments in which (i) the initial RR20 procedure was immediately followed by a reversed RR20 task without a preceding devaluation session, definitively ruling out motivational confounds (Rev3Maj1) and (ii) showing that 3xFAD mice perform well in a reversal test run in a radial maze, demonstrating that their deficit in cognitive flexibility is selective to the goal-directed/instrumental domain (Rve3Maj2).

The following issues were also satisfactorily addressed:
Rev3Maj3 (limiting the behavioral deficit to the PL/IL circuit rather than the mPFC as a whole), Rev3Maj6 (detailing the timing of isoflurane exposure relative to behavioral testing), Rev3Maj7 (discussing extensively why off-target (AchR) effects of compound 21 injections are unlikely), Rev3Maj8 (considering the potential involvement of hippocampal neurogenesis in cognitive flexibility), RevMaj9 (using two-way RM ANOVAs with genotype and session as main factors to validate inter-strain behavioral differences through significant genotype x session interactions), Rev4Maj10 (specifying the cognitive flexibility deficit of 5XFAD mice at the light of the Pierce-Messick et al., 2025 distinction between a deficit in updating previously learned action-outcome associations and a primary impairment in goal-directed control.

The "Supplementary Data" section, which includes more experiments than the "Main Data" section, has been left unchanged but the authors give justifications for that (Rev3Maj4). Also, the fact that behavioral and neurobiological investigations conducted at different ages partially support causal relationships has been taken into account (Rev3Maj5).

With regard to our area of expertise, we consider the manuscript suitable for publication.

Reviewer #4

(Remarks to the Author)
I appreciate the authors' acknowledgment of the molecular dimension in their revised discussion. However, the added sentence remains largely generic and does not substantively address the concern raised. For a study of this scope published in Nature Communications, I would expect the authors to leverage their own circuit-level findings as a foundation for specific molecular hypotheses, even in the absence of direct experimental evidence.
For instance, the documented mPFC hyperexcitability could invite speculation regarding reduced voltage-gated potassium channel expression or functon(e.g., Kv4.2 or Kv1 subfamily members), or impaired afterhyperpolarization conductances — all of which have precedent in amyloid-driven neuronal dysfunction. Similarly, the enhanced glutamatergic corticostriatal transmission they report could be linked to known amyloid-associated mechanisms such as AMPA receptor subunit upregulation, reduced astrocytic glutamate clearance via GLT-1/EAAT2, or increased presynaptic release probability. Finally, the suppression of striatal cholinergic interneuron firing could be discussed in the context of M2 receptor dysregulation or nicotinic receptor desensitization, both of which are plausible downstream consequences of the circuit pathology described.

Reviewer #5

(Remarks to the Author)

I co-reviewed this manuscript with one of the reviewers who provided the listed reports. This is part of the Nature Communications initiative to facilitate training in peer review and to provide appropriate recognition for Early Career Researchers who co-review manuscripts.

Version 2:

Reviewer comments:

Reviewer #1

(Remarks to the Author)
The authors have substantially improved the manuscript. The revised version addresses the main conceptual concern by framing the strongest causal conclusion at the level of upstream mPFC→DMS corticostriatal hyperactivity, while treating dMSN-->CIN-->ACh changes as associated downstream mechanisms rather than as a proven necessary/sufficient behavioral bottleneck. This is reflected in the Abstract, where cholinergic changes are described as coincident with enhanced corticostriatal transmission and the behavioral rescue is attributed to sustained corticostriatal inhibition rather than to direct CIN/ACh rescue alone.
The behavioral framing is also improved. The authors now focus the claim on impaired instrumental reversal learning/action–outcome updating, and the merged manuscript appropriately distinguishes this from broader spatial learning deficits, which are absent at 3 months and emerge later. The added clarification that the data do not establish a generalized executive dysfunction phenotype is appropriate and important.
The statistical response is mostly satisfactory. The authors performed the requested mixed-effects reanalysis for key electrophysiological datasets, and the reported results support the robustness of the main conclusions after accounting for cells nested within animals. The manuscript now states that behavioral and histology data are analyzed at the animal level, live imaging at the slice level, and electrophysiology at the neuron level with animal numbers provided in legends/source data. This substantially improves transparency.
The revisions to Figure 4 and the seizure/EEG interpretation are also acceptable. The revised manuscript now states viral targeting/exclusion criteria for mPFC terminal stimulation in DMS and clearly separates ex vivo pathway-specific evidence from seizure/EEG evidence of broader network hyperexcitability.
I do not think additional experiments are required at this stage. In particular, I would no longer insist on a CIN/ACh rescue experiment, provided the authors preserve the current more cautious interpretation. However, I recommend minor textual revision before acceptance to remove a few residual overstatements:
In the Abstract, “pathological corticostriatal hyperactivity acts as a circuit mechanism underlying early cognitive inflexibility in Alzheimer’s disease” should be softened to “contributes to early cognitive inflexibility in a mouse model of Alzheimer’s disease” or similar. The upstream circuit manipulation supports causality in 5xFAD mice, but the current wording remains slightly broad for human AD.
In the Discussion, the sentence stating that CIN hypoactivity “results from excessive GABAergic inhibition” and that this cascade “compromises striatal output and contributes to behavioral rigidity” should be softened to “is consistent with” and “may contribute,” because the relative contribution of striatal cholinergic restoration to behavioral rescue is explicitly unresolved.
The phrase “excessive reactivation” still appears in the Discussion, despite the authors’ response that this wording was softened. I recommend changing it to “increased reactivation” or “greater reactivation,” consistent with the revised Figure 2 title.
The mixed-effects reanalysis should be included in the manuscript or supplement/source data, not only in the rebuttal, because it directly addresses the independence concern for the electrophysiology datasets.

Open Access This Peer Review File is licensed under a Creative Commons Attribution 4.0 International License, which permits use, sharing, adaptation, distribution and reproduction in any medium or format, as long as you give appropriate credit to the original author(s) and the source, provide a link to the Creative Commons license, and indicate if changes were made.
In cases where reviewers are anonymous, credit should be given to 'Anonymous Referee' and the source.
The images or other third party material in this Peer Review File are included in the article's Creative Commons license, unless indicated otherwise in a credit line to the material. If material is not included in the article's Creative Commons license and your intended use is not permitted by statutory regulation or exceeds the permitted use, you will need to obtain permission directly from the copyright holder.
To view a copy of this license, visit https://creativecommons.org/licenses/by/4.0/

Point-by-point response to reviewers' concerns

To directly address the reviewers' comments, we performed additional experiments and conducted new data analyses, and we have incorporated the resulting data as 11 figures in the revised manuscript and/or this rebuttal letter. Below, we provide a detailed, point-by-point response to each comment. The table below summarizes the major experiments and analyses completed in response to the reviewers' requests. For clarity, the reviewers' original comments are presented in italicized text. We believe these additions substantially strengthen the manuscript and improve its overall impact.

Summary of New Experiments and Results

#	New experiment & main finding (new figures if applicable)	Key conclusion	Questions addressed
1. Exp	Motivation test during reversal instrumental learning (Reb_Figure 6/Supplementary Fig. 4)	5xFAD mice exhibited no significant motivational deficits compared to WT mice.	Rev2_Maj1* Rev3_Maj1
2. Exp	Radial maze assessment of spatial learning during the early stage of AD (Reb_Figure 10/Supplementary Fig. 1).	5xFAD mice showed no significant impairments relative to WT mice during either initial acquisition or reversal learning in the radial maze.	Rev3_Maj2
3. Result	Plaque size analysis following hM4Di activation. (Reb_Figure 1)	Plaque size was not significantly reduced in the hM4Di group compared to the mCherry control group.	Rev1_Maj6
4. Result	Aβ staining in striatal sections. (Reb_Figure 2/ Supplementary Fig. 9)	No Aβ accumulation was detected in the striatum of 3–4-month-old 5xFAD mice.	Rev1_Maj6
5. Result	Sex-based comparison of Aβ-covered area. (Reb_Figure 3)	No significant sex differences were observed.	Rev1_Maj7
6. Result	Sex-based comparison of behavior in Figure 1. (Reb_Figure 4)	No significant sex differences were observed	Rev1_Maj8
7. Result	Passive and intrinsic membrane properties of mPFC pyramidal neurons in WT and 5xFAD mice. (Reb_Figure 5)	No significant differences were detected between genotypes.	Rev1_Min3**
8. Result	60x confocal image of an ArcTRAP-labeled neuron (Reb_Figure 7)	Dendritic spines are not reliably resolved at ArcTRAP labeling density and signal-to-noise level.	Rev2_Maj3

9. Result	Neuron-to-animal ratios in control versus experimental groups comparison. (Reb_Figure 8)	Comparable numbers of recorded neurons and neuron-to-animal ratios in control versus experimental groups.	Rev2_Maj4
10. Result	Functional assessment of optical recruitment analysis. (Reb_Figure 9)	Optical recruitment was similar between groups.	Rev2_Maj4
11. Result	Off-target binding and CSF exposure of Compound 21 analysis (Reb_Figure 11)		Rev3_Maj7
12. Result	Instrumental performance and outcome devaluation index. (Reb Figure 12)	Instrumental performance did not differ between groups, whereas the devaluation index showed a significant genotype effect.	Rev3_Maj9

*Rev2_Maj1: Reviewer 2, major concern 1
**Rev1_Min3: Reviewer 1, minor concern 3
Exp: Experiment that is required by the reviewer
Result: New data analysis

Reviewers' Comments:

Reviewer #1 (Remarks to the Author):
In this manuscript, the Authors study the 5xFAD mouse, a mouse model of Alzheimer's disease, using electrophysiology and behavioural techniques, combined with chemogenetic and optogenetic stimulation, to investigate the circuits between the medial prefrontal cortex (mPFC) and dorsomedial striatum (DMS) and their relation with deficits in reversal learning. In particular, they demonstrate with ex-vivo recordings that mPFC neurons are hyperactive in 4-month-old 5XFAD mice, causing increased activity of striatal dMSN neurons, which are directly innervated by mPFC neurons. The hyperactivity of dMSNs, in turn, excessively inhibits cholinergic neurons in the striatum (CINs), which then release less acetylcholine (ACh). Associated with this alteration in the neural circuit, 5XFAD mice show deficits in cognitive flexibility during behavioural tests, failing reversal learning tasks. Using the chemogenetic approach, the authors selectively inhibit the mPFC-Striatum circuit, demonstrating that ACh release in the striatum is restored, cognitive deficits are recovered, and the accumulation of Aβ plaques in the mPFC decreases. The methodology applied to this work is pretty solid and most results are convincing and well explained. Following are some comments and suggestions to be applied to improve the quality and readability of the manuscript.

Major comments:
Rev1_Maj1

1. *Generic comment: The different stats tests are mentioned in the Figure Legends but the results of the tests (p and F values) are currently in the text; to facilitate the reading I would recommend moving everything in the legends, to be able to more easily follow the figures.*

Response: We thank the reviewer for this helpful comment. We have now moved the statistical information (including p and F values) from the main text to the figure legends, which improves the readability of the manuscript and allows the figures to be more easily followed.

Rev1_Maj2

2. Line 98: are the stats mentioned based on paired tests? This is not clear from the figure legends.

Response: We thank the reviewer for pointing this out. The statistics reported in Fig. 1C were derived from a two-way repeated-measures ANOVA, not from paired t-tests. Specifically:

- Between-subject factor**:** Genotype (WT vs. 5xFAD)
- Within-subject factor (repeated measure): Training session

For the initial training phase (Fig. 1C):

- The statistic reported on line 97 ($F_{1,20}$ = 0.4, p = 0.534) corresponds to the main effect of genotype (between-subject factor), indicating no significant difference between WT and 5xFAD mice in overall lever-pressing rates during early training.
- The statistic reported on line 98 ($F_{1,13}$ = 46.649, p < 0.001) corresponds to the main effect of session (training), which was treated as a repeated measure. This significant effect reflects increased responding across sessions, indicating intact acquisition of the task.

Thus, neither of these values was derived from a paired test. Instead, both were obtained from the same two-way repeated-measures ANOVA model, with genotype as a between-subject factor and session as a within-subject (repeated) factor. We revised the figure legend to explicitly state that a two-way repeated-measures ANOVA was used to avoid confusion.

Rev1_Maj3a

3. Fig.2:
a. IF panels (B,C and D,E): the background fluorescence appears higher in 5xFAD mice in the initial condition. Are these IFs taken with same laser settings for WT and AD mice? I would overall suggest changing the representative images as these do not allow to appreciate differences (of lack there of) in Arc expression. This stands for both panels 2B,C and for 2D,E. Perhaps DAPI or higher magnification images(as in 2B) would also help, as performed for the dentate gyrus

Response: We thank the reviewer for this helpful comment and for pointing out the concern regarding background fluorescence and image representation.

Regarding the panels B, C, D, and E, they do not represent immunofluorescence staining. Instead, these experiments were performed using Arc-CreER;Ai14 mice (Figure 2A), in which

neuronal activity–dependent Arc expression drives Cre recombination and results in intrinsic tdTomato expression following 4-hydroxytamoxifen (4-OHT) administration. Therefore, the tdTomato signal shown in these panels is not derived from antibody-based immunofluorescence, but from genetically encoded reporter expression.

Following the reviewer's suggestion, we have replaced the representative images shown in previous panels B, C, D, and E (now Figure 2B and D) with new examples that better reflect the quantified data. In addition, we have added enlarged, higher-magnification images to improve visualization of Arc-expressing neurons and to facilitate comparison between genotypes, similar to what was done for the dentate gyrus. We thank the reviewer for raising this point, which helps us improve the quality of the images.

Rev1_Maj3b

b. Statistical analysis: according to the authors's hypothesis, mPFC or DMS neurons become hyperactive after the reversal phase of the test but are normally active during the initial phase. The analysis shown in Figs 2B,C and 2D,E confronts control vs 5xFAD neurons but the confrontation of 5xFAD initial vs reversal is missing. A 2-way ANOVA might be a more appropriate test to confront all 4 groups (WT and 5xFAD, initial and reversal phase) for both the mPFC and DMS.

Response: We thank the reviewer for this thoughtful and constructive comment. We apologize that our original description did not clearly articulate our hypothesis, which may have caused confusion. Our intention was not to suggest that mPFC or DMS neurons become intrinsically hyperactive specifically after the reversal phase. Rather, the purpose of Figure 2 was to demonstrate that a larger population of neurons is recruited during reversal learning in 5xFAD mice, and that a substantial proportion of these neurons are reactivated from the ensemble engaged during the initial learning phase. Accordingly, our conclusions focus on patterns of neuronal recruitment and reactivation, rather than intrinsic changes in neuronal excitability.

Following the reviewer's suggestion, we have now performed a two-way ANOVA including genotype (WT vs. 5xFAD) and task phase (initial vs. reversal) as factors for both the mPFC and DMS datasets. This analysis revealed a significant genotype × task phase interaction in both regions (mPFC: $F_{(1, 18)} = 12.40$, $p = 0.0024$; DMS: $F_{(1, 18)} = 21.63$, p = 0. 0002), indicating that the pattern of neuronal recruitment across task phases differs between genotypes. Specifically, 5xFAD mice showed a significant increase in the number of recruited neurons from the initial to the reversal phase, whereas WT mice did not exhibit such an increase.

Post hoc comparisons further confirmed significant differences between WT and 5xFAD mice during the reversal phase (mPFC: adjusted $p = 0.0378$; DMS: adjusted $p = 0.0004$), but not during the initial phase, consistent with our original pairwise analyses. Importantly, the two-way ANOVA supports and strengthens our previous conclusions while providing a more comprehensive statistical framework, as suggested by the reviewer. The revised two-way ANOVA results have now replaced the original statistical analyses in Figures 2B–E. We sincerely appreciate this suggestion, which has improved the statistical rigor and clarity of our analysis.

Rev1_Maj4

4. Fig. 3: rappresentative traces in Fig.3C should be changed, as the increase in frequency is not evident in this scaling. Please add also higher-scaling traces (scaled for both amplitude and time) to be able to appreciate differences in amplitude and frequency. Also, the 'burst-like' activity of sEPSCs in AD mice is highly atypical, and should be described further in the text. Was this a frequent event in all patches? Was the analysis of these huge (nA) events included in the overall analysis shown in panels D and E? I would expect the mean amplitude of sEPSCs in AD mice to be much higher that what shown in D, given that many events, even outside the 'bursts', reach the 500pA-1nA scale.

Response: We thank the reviewer for this important and insightful comment. We have revised both the sample traces and the corresponding text to clarify the nature and contribution of large-amplitude sEPSCs observed in 5xFAD mice.

1. **Representative traces and scaling.** We have replaced the original representative traces in Fig. 3C with new traces that better illustrate the increase in sEPSC frequency in 5xFAD neurons. In addition, we now include enlarged traces with expanded amplitude and time scales to allow clearer visualization of both frequency and amplitude differences. A representative enlarged trace highlights the large-amplitude events observed selectively in 5xFAD recordings.
2. **Inclusion and quantification of large-amplitude events.** All detected sEPSCs, including large-amplitude events, were counted as independent events and included in the analysis shown in panels D and E. To better address the reviewer's concern regarding event amplitude, we have added a new panel (Fig. 3D) showing the distribution and cumulated of sEPSC amplitudes in WT and 5xFAD mice. This analysis reveals that although the majority of events in both groups are small (<50 pA), 5xFAD neurons exhibit a higher proportion of large-amplitude events (>100 pA), with some recordings displaying rare events exceeding 500 pA. Because small-amplitude events constitute the majority of sEPSCs, the overall mean amplitude is not dramatically increased, consistent with the values shown in panel E. We now explicitly describe this point in the Results section (Lines 181–184).
3. **Burst-like sEPSC activity.** We agree with the reviewer that the burst-like synaptic activity observed in some 5xFAD neurons is atypical. In the revised manuscript, we clarify that these large-amplitude, burst-like sEPSC events were not observed in all recordings but occurred recurrently in a subset of patches from 5xFAD mice. This observation is now described explicitly in the Results, and its interpretation is discussed cautiously to avoid overstatement. Specifically, we note that such activity may be related to the previously proposed pathological feedback loop between Aβ and neuronal hyperactivity, in which activity-dependent Aβ release promotes cortical hyperexcitability and impairs glutamate reuptake, thereby enhancing glutamatergic transmission. We add this in the Discussion (Lines 418-421).

Rev1_Maj5

5. *Fig. 4C,D: please add representative traces. Overall, the results in Fig. 4D are puzzling: if mPFC glutamatergic neurons are hyperexcitable, an increase in the frequency of sEPSCs in*

MSNs should be seen, which is not the case from what is shown in the plot. It is probable that these MSN neurons recorded in the striatum are not directly innervated by the mPFC, resulting in misleading information (see lines 206-208, that are contradicted further on). This issue is important since the authors claim that the hyperactivity of the dMSNs is directly driven by the mPFC (line 210), and they clearly demonstrate with optogenetic stimulation in panel Fig.4F-H. Thus, I recommend removing the sEPSC results from the paper, to be substituted by experiments in mPFC-retrogradely labelled MSN neurons (or in experiments of pre-post optogenetic mPFC stimulation)

Response: We thank the reviewer for this insightful comment. We agree that spontaneous EPSC recordings do not allow identification of the presynaptic source of input and therefore cannot directly demonstrate mPFC-specific innervation of MSNs. To avoid potential misinterpretation and improve clarity, we have removed the former Fig. 4C–D from the revised manuscript.

Importantly, our central conclusion is supported by the optogenetic experiments (Fig. 4E–H and 4I–L; Fig.4E, F, H ,I in the revised manuscript), which selectively assess mPFC inputs onto identified dMSNs and iMSNs. These pathway-specific recordings demonstrate enhanced mPFC-to-dMSN transmission in 5xFAD mice, while mPFC-to-iMSN transmission remains unchanged. Thus, removal of the spontaneous EPSC data does not alter our primary findings. We appreciate the reviewer's suggestion, which has strengthened the focus and clarity of this section.

Rev1_Maj6

6. In Fig. 6B, the claim that Aβ levels are decreased is not very convincing, at least based on there presentative figure. Either fluorescence intensity or size of individual plaques should also be analyzed. At this age, does this model also show an accumulation of Aβ plaques in the striatum? If so, I suggest analyzing the reduction of Aβ plaques in this area as well.

Response: We thank the reviewer for this helpful comment. Following the reviewer's suggestion, we performed an **additional analysis** of individual plaque size in the cortical region and have included these results in the rebuttal (Reb_Fig 1). However, with the current sample size, we did not detect a statistically significant difference between groups. In the revised manuscript, we primarily present Aβ plaque burden as percent area covered, a widely accepted metric in the field that reflects the overall area affected by amyloid deposition (Bero et al., 2011; Huynh et al., 2017; Wasen et al., 2024; Golden et al., 2025). To improve the clarity of the representative images, we have updated main Figure 6B (also attached below) by adding a blue dashed outline to indicate the approximate overall Aβ-positive area, in addition to the white dashed outline marking the predefined region of interest used for quantification. We thank the reviewer for raising this concern, which helps improve the quality of our revised manuscript.

Regarding the reviewer's question about striatal Aβ pathology at this age, we include additional histology from 3-month-old 5xFAD;ChAT-eGFP mice (Reb_Fig 2, now as supplementary figure 9), which shows robust cortical Aβ staining but no detectable Aβ-positive plaques in the dorsal striatum. Given the absence of baseline striatal plaque deposition at this time point, we did not further analyze treatment-associated changes in the striatum.

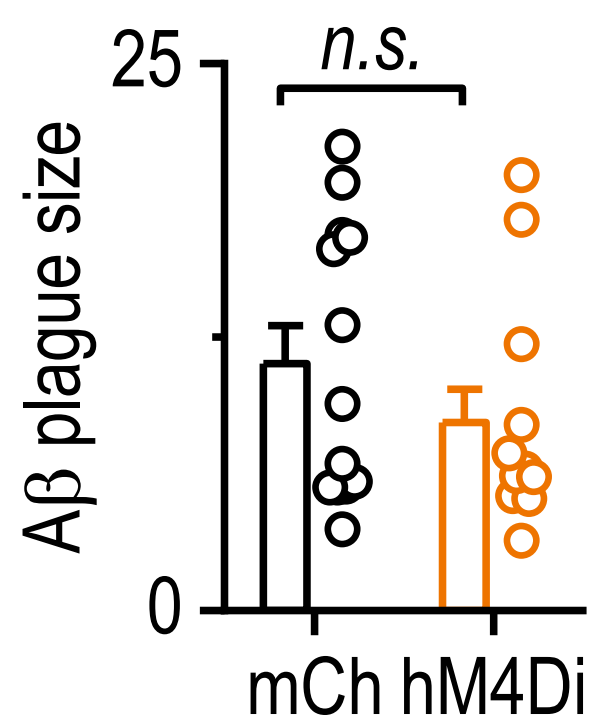

Reb_Figure 1. Plaque size analysis
No significant difference in Aβ plaque size was detected between the mCh and hM4Di groups in the mPFC. Mann–Whitney rank-sum test, U = 60, p = 0.341. n = 13 (mCh); 12 (hM4Di).

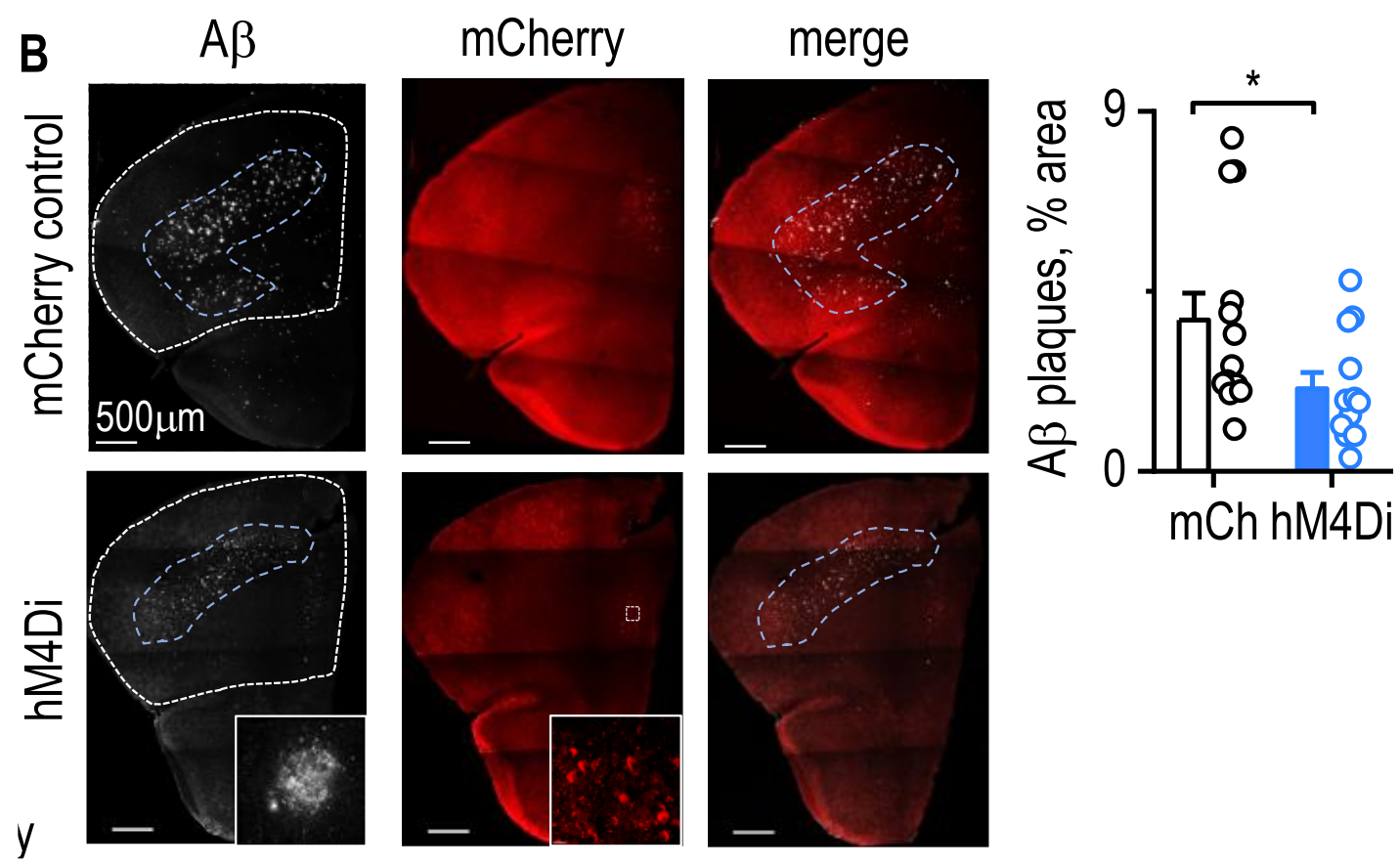

Figure 6. B, Representative images of Aβ immunostaining in the mPFC. The blue dashed outline indicates the approximate overall Aβ-positive area; the white dashed outline marks the predefined region of interest used for quantitative analysis. hM4Di-injected 5xFAD mice exhibited significantly reduced Aβ plaque coverage compared to mCherry-injected controls. Mann-Whitney Rank Sum Test, U = 37, p = 0.028. *p < 0.05. n = 13 mice (mCh) and 12 mice (hM4Di).

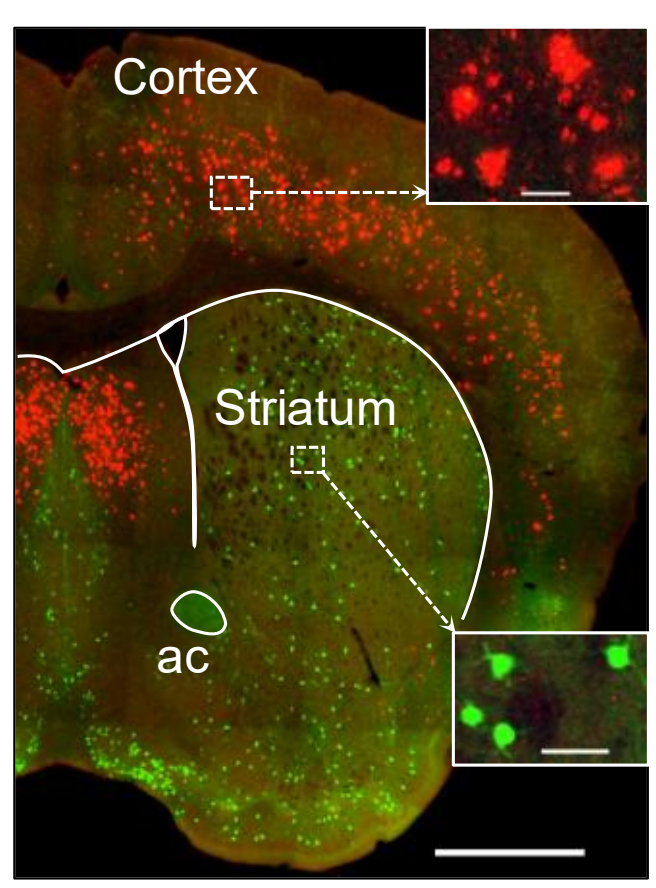

Reb_Figure 2 (same as Supplementary Figure 9) Aβ deposition in the cortex but not the striatum of 3-month-old 5xFAD;ChAT-eGFP mice.

Representative images showing Aβ (red) accumulation in the cortex, with no detectable Aβ signal in the dorsal striatum. Cholinergic interneurons (CINs) are labeled with eGFP (green). ac, anterior commissure. Scale bars: 1 mm (main panel), 50 µm (inset).

Rev1_Maj7

7. In addition, do you see a different rate of decline in Aβ between male and female mice? Female 5xFAD mice show a significantly greater Aβ plaque burden compared to males, particularly in the cortex and striatum. The authors might want to discriminate between the two sexes.

Response: We thank the reviewer for this important and insightful comment regarding potential sex differences in Aβ pathology. To directly address this question, we performed a sex-stratified analysis of Aβ plaque burden in 5xFAD mice using a two-way ANOVA with Sex and Treatment as factors (Reb_Fig 3).

This analysis revealed no significant Sex × Treatment interaction ($F_{(1,21)} = 0.209$, $p = 0.653$), indicating that the effect of hM4Di manipulation on Aβ plaque burden does not differ between male and female mice. We observed a trend toward a main effect of Sex ($F_{(1,21)} = 3.823$, $p = 0.064$), consistent with previous reports suggesting higher amyloid burden in female 5xFAD mice; however, this effect did not reach statistical significance in our cohort.

Because sex did not significantly modify the treatment effect or alter the interpretation of our primary findings, we have retained the combined-sex analysis in the main manuscript for clarity and consistency. Nevertheless, we agree that sex-dependent differences in amyloid pathology are biologically important and warrant further investigation in larger, adequately powered cohorts. We appreciate the reviewer's suggestion, which has strengthened the rigor of our analysis.

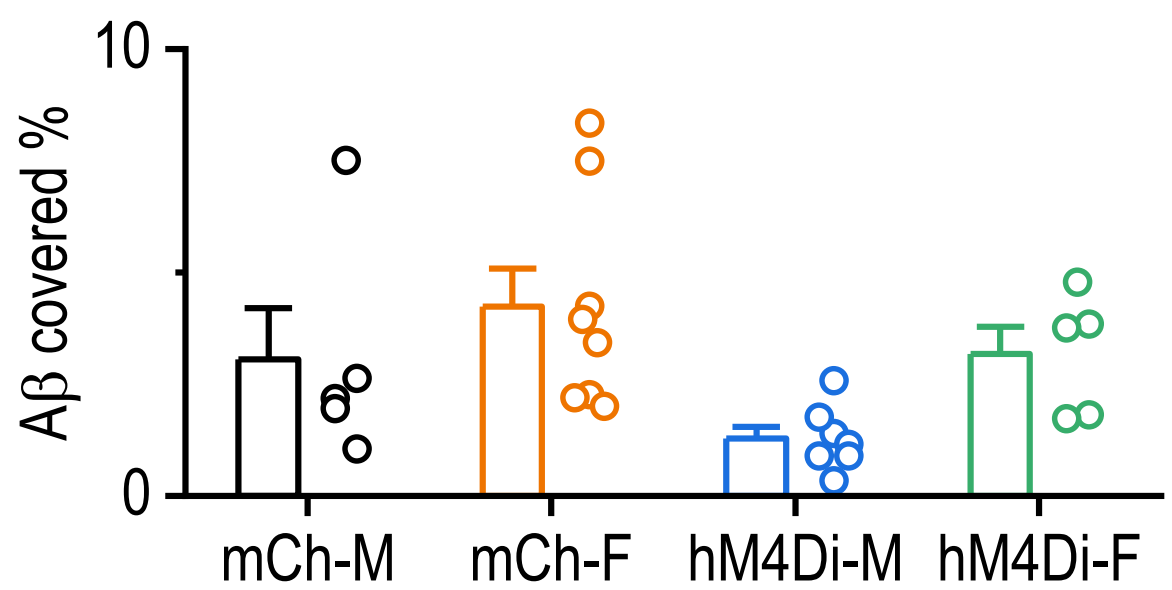

Reb_Figure 3. Aβ-covered area analyzed by sex.

Quantification of the percentage of cortical area covered by Aβ plaques in mCh and hM4Di groups, separated by sex. Individual data points represent single animals. Sample sizes: mCh male, n = 5; mCh female, n = 8; hM4Di male, n = 7; hM4Di female, n = 5.

Rev1_Maj8

8. Line 463: Males and females were pooled in the experiments. Before doing so, did the authors ensure that there were no significant differences in the experimental results due to sex?

Response: We thank the reviewer for raising this important point. Prior to pooling male and female mice, we evaluated potential sex effects. In our pilot experiments, sex was included as a factor, and we did not detect any significant sex-dependent differences in the measured behavioral outcomes.

To further address this concern, we performed an additional analysis of potential sex differences in the behavioral datasets presented in Figure 1 of the main manuscript. Across all behavioral measures, including lever-press rate, outcome devaluation performance, and learning index (Reb_Figure 4), we found no significant differences between male and female 5xFAD mice. These results indicate that cognitive function and behavioral performance were comparable between males and females.

This finding is consistent with previous large-scale and systematic characterization of the 5xFAD model, demonstrating that, at early disease stages, core AD-related phenotypes are largely comparable between males and females (Oblak et al., 2021). We agree that sex differences represent an important consideration in Alzheimer's disease research. Investigating sex-dependent mechanisms will be an important direction for future studies specifically designed for that purpose, and we appreciate the reviewer's suggestion.

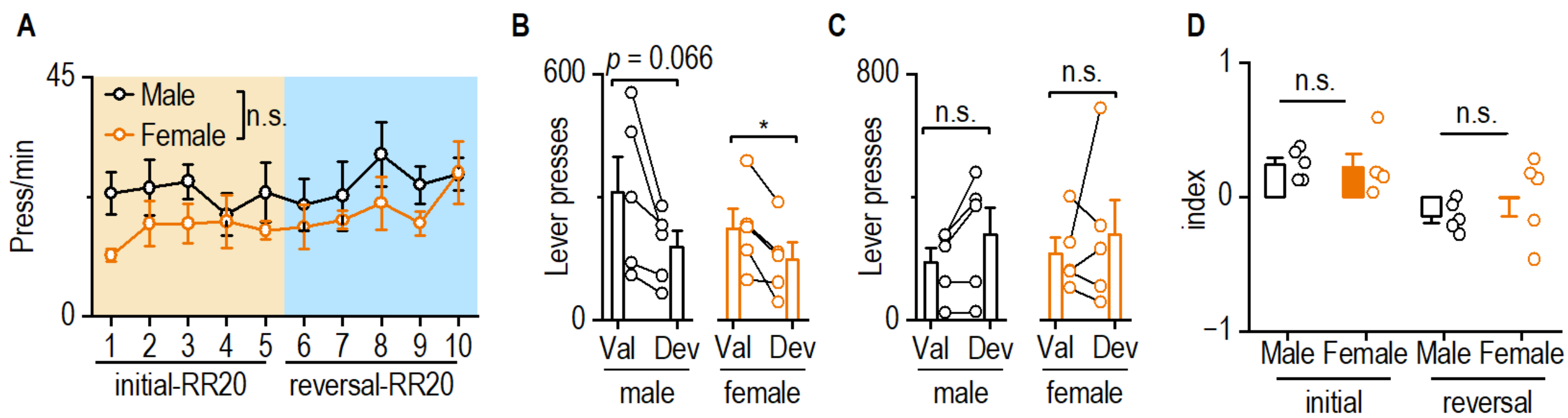

Reb_Figure 4. Sex does not influence instrumental learning or reversal performance in 5xFAD mice.

(A) Lever-press rate across training. Male and female 5xFAD mice showed comparable response rates during both initial random-ratio 20 (RR20) training and reversal RR20 sessions (no significant sex effect). Two-way RM ANOVA, $F_{(1, 8)} = 2.026$, $p = 0.1925$. n = 5 male and 5 female 5xFAD mice.

(B) Outcome devaluation test following initial training. Both male and female mice made more responses on the valued lever than on the devalued lever, indicating acquisition of the initial action–outcome contingency. Pair t-test, $t_4 = 2.508$, $p = 0.066$ for male; $t_4 = 3.659$ $p = 0.022$ for female. n = 5 male and 5 female 5xFAD mice.

(C) Outcome devaluation test following reversal training. Neither male nor female mice showed a preference for the valued over the devalued lever, indicating failure to update the action–outcome contingency after reversal. Pair t-test, $t_4 = -2.273$, $p = 0.085$ for male; Wilcoxon Signed Rank Test $Z = 0.135$ $p = 1$ for female. n = 5 male and 5 female 5xFAD mice.

(D) Learning index during initial and reversal phases did not differ between sexes. Two-way RM ANOVA, $F_{(1, 8)} = 0.293$, $p = 0.603$; Post-hoc analysis: comparisons for factor: sex within initial $p = 0.888$; Comparisons for factor: sex within reversal: $p = 0.314$. n = 5 male and 5 female 5xFAD mice.

Minor comments:

Rev1_Min1

1. Fig. 3G and 5G: example traces are missing

Response: We thank the reviewer for pointing this out. Representative traces have now been added to Figures 3G and 5G.

Rev1_Min2

2. Fig 4E and 4I: example traces are missing

Response: We thank the reviewer for pointing this out. Representative traces have now been added to Figures 4E and 4I (now Figure 4D and 4G).

Rev1_Min3

3. Given the increase excitability of mPFC neurons, authors are requested to provide a more detailed analysis of the current-clamp recordings. It would also be advisable to analyse their membrane properties, such as resting membrane potential, the input resistance, the rheobase, the firing threshold ecc. This might provide more indications as to the hyperexcitability of these cells.

Response: We thank the reviewer for this helpful suggestion. We performed additional analyses of intrinsic membrane properties of mPFC pyramidal neurons from WT and 5xFAD mice, including resting membrane potential (RMP) and rheobase (minimum current required to evoke the first action potential). As shown in Reb_Figure 5A, rheobase did not differ significantly between groups (rheobase: $p = 0.809$). Although 5xFAD neurons displayed increased firing frequency, the difference emerged at higher current injections, while responses near the firing threshold were comparable (Reb_Figure 5B, same as main Figure 3B). The membrane potential (RMP) (Reb_Figure 5C, same as main Figure 3C) is also similar between genotypes (RMP: $p = 0.749$). We included the RMP data in the main figure as a control property. These results indicate that the enhanced excitability is not due to altered passive membrane properties but rather reflects increased spike output gain. We appreciate the reviewer's suggestion, which helped clarify the mechanism underlying the observed hyperexcitability.

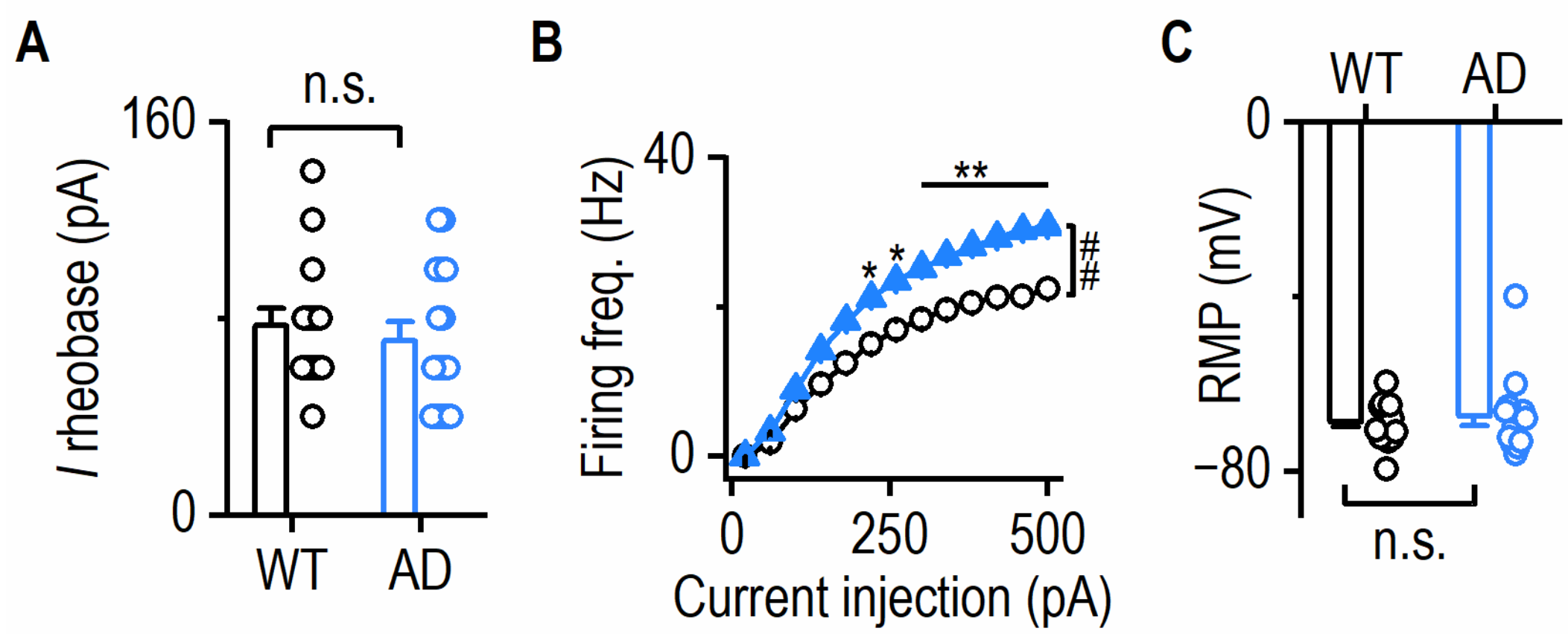

Reb_Figure 5. Passive and intrinsic membrane properties of mPFC pyramidal neurons in WT and 5xFAD mice.

(A) Rheobase, defined as the minimal current injection required to elicit the first action potential. No significant difference was observed between groups (unpaired t test, t_{30} = 0.243, p = 0.809).

(B) mPFC neurons had a significantly higher frequency of evoked firing in 5xFAD animals than WT controls. Two-way RM ANOVA with Greenhouse–Geisser correction, genotype effect: $F_{(1,29)}$ = 13.10, p = 0.0011. $^{##}p < 0.01$; Sidak's multiple comparisons post-hoc analysis, $^{*}p < 0.05$, $^{**}p < 0.01$ versus WT at the same stimulating intensities. Current injection: 300 pA, 1 sec. n = 15 neurons from 3 mice (WT) and 16 neurons from 5 mice (5xFAD).

(C) Resting membrane potential (RMP). No significant difference was detected between WT and 5xFAD mice (Mann–Whitney. U = 119.0, p = 0.749). n= 15 neurons from 3 WT mice and 16 neurons from 5 5xFAD mice.

Rev1_Min4

4. Fig. 2H-I. From the merge, in 5XFAD group the co-labelling between co-expression of tdTomato and c-Fos in mPFC and DMS neurons is not clearly visible. Please change the representative image or add higher magnification images.

Response: We thank the reviewer for raising this concern. We have added higher-magnification representative images in Figures 2H–I to more clearly illustrate signal overlap.

Rev1_Min5

5. in Fig. 3C, change AD with 5xFAD for consistency, and correct uA to μA in panel 3F

Response: We thank the reviewer for pointing this out. We have updated the labeling in Figure 3C from “AD” to “5xFAD” for consistency and corrected “uA” to “μA” in Figure 3F.

Rev1_Min6

6. in Fig 5K: what does the Y scale indicate? fluorescence intensity ΔF/F?

Response: We thank the reviewer for this question. The Y-axis is ΔF/F. We have now updated the Y-axis label in Fig. 5K accordingly.

Rev1_Min7

7. Figure Suppl 5F. Sample trace in EEG recordings showing spikes before and after pilocarpine i.p. injection is form WT or 5XFAD mice? Anyway, it is advisable to show the trace in both groups for comparison.

Response: We thank the reviewers for this suggestion. We now add a sample trace for WT. Clearly, the onset of seizure spike is earlier in the AD animal than in WT animal after pilocarpine injection. The revised figure is now in Sup. Figure 7F.

Rev1_Min8

8. Authors are requested to provide more details on the regions they recorded. For example, specify in which subregions of the medial PFC the electrophysiology recordings were performed, or even more specifically, in which layer?

Response: We thank the reviewer for this comment. We have updated the Methods section to provide additional details on the regions targeted for electrophysiological recordings. Specifically, recordings were performed in the medial prefrontal cortex at anteroposterior coordinates ranging from AP+2.58 to AP +1.78 (Prelimbic cortex, PrL, based on the atlas), primarily in cortical layer 5. In experiments involving AAV injections, recordings were performed from mCherry-positive neurons in the cortical layer 5, as now clarified in the Figure legend section (Lines 1150-1151).

Rev1_Min9

9. Electrophysiology methods: Please specify which antagonists were used for the ex-vivo electrophysiology recordings and at what concentrations. Add duration of optical or electrical stimulation

Response: We thank the reviewer for this comment. We have now specified the antagonists (100-μm picrotoxin for EPSCs and 10-μM DNQX for IPSCs) used for the ex vivo electrophysiology recordings, including their concentrations, and added details regarding the duration of optical and electrical stimulation in the Methods section (Lines 697-699, 703-704, and 718).

Rev1_Min10

10. Please specify more clearly in the text at what age the experiments are performed. From this point of view, the text is a bit confusing.

Response: We thank the reviewer for this comment. We have now clarified the age at which each experiment was performed and added explicit age descriptions throughout the text and figure legend (e.g. Lines 83, 1150 and 1179) to improve the clarity of the manuscript.

Reviewer #2 (Remarks to the Author):
This is a conceptually solid and potentially high-impact study addressing how corticostriatal hyperactivity contributes to early cognitive inflexibility in the 5xFAD model of Alzheimer's disease. The authors propose that amyloid-β–induced hyperactivity within the medial prefrontal cortex–to–dorsomedial striatum circuit enhances direct pathway medium spiny neuron inhibition of cholinergic interneurons, thereby reducing striatal acetylcholine release and impairing reversal learning. Sustained chemogenetic inhibition of this projection reportedly decreases cortical amyloid-β accumulation, normalizes glutamatergic transmission, restores cholinergic signaling, and rescues cognitive flexibility.
However, the following points merit clarification or strengthening before publication.

Rev2_Maj1
Although the manuscript describes a prefeeding procedure before devaluation testing ("one-hour free access to 40 randomly assigned purified or grain pellets"), this manipulation by itself does not establish whether satiety and reinforcer valuation are equivalent across genotypes. No data are reported on the amount consumed, the rate of consumption, or the efficacy of satiety induction. To exclude motivational or palatability confounds, the authors should quantify prefeeding consumption and/or perform a brief post-prefeed preference or progressive-ratio test to confirm outcome-specific value shifts in both groups. Because motivation and reinforcer value directly influence devaluation performance, the authors should measure satiety effectiveness to rule out behavioral confounds.

Response: We thank the reviewer for emphasizing the importance of verifying satiety induction and reinforcer valuation. To directly address this concern, we performed additional experiments in an independent cohort of WT and 5xFAD mice.

During the one-hour prefeeding period, in which animals were given free access to 40 pellets, we quantified both total consumption and consumption rate. WT and 5xFAD mice consumed comparable amounts of pellets and at similar rates, indicating similar exposure to the satiety manipulation across genotypes (Reb_Figure 6, E and F).

To further assess outcome-specific value shifts, we conducted a post-prefeeding free-choice test between the pre-fed (devalued, dev pel.) outcome and the non-prefed (valued, val pel.) outcome. Both genotypes showed a comparable reduction (t_{14} = 0.2601, p = 0.7986) in consumption of the prefed outcome relative to the valued outcome (Reb_Figure 6, G and H), consistent with effective outcome-specific satiety cross WT and 5xFAD mice.

In addition, to assess baseline motivation to work for the food reinforcers independent of satiety manipulation, we examined performance in a progressive ratio (PR) task. WT and 5xFAD mice showed comparable total lever pressing, breakpoint, and cumulative responding for both grain and purified pellets (Reb_Figure 6, I–N). These results indicate similar baseline effort-based responding for both reinforcers across genotypes and provide additional control for motivational differences that could influence performance in the devaluation task.

These data have now been included in the manuscript as Supplementary Figure 4 and referenced in the Results section (Lines 125–132), and they argue against differences in satiety

induction or reinforcer valuation as major confounding factors in the devaluation task. We thank the reviewer for raising this important concern, which helps us improve the rigor of our manuscript.

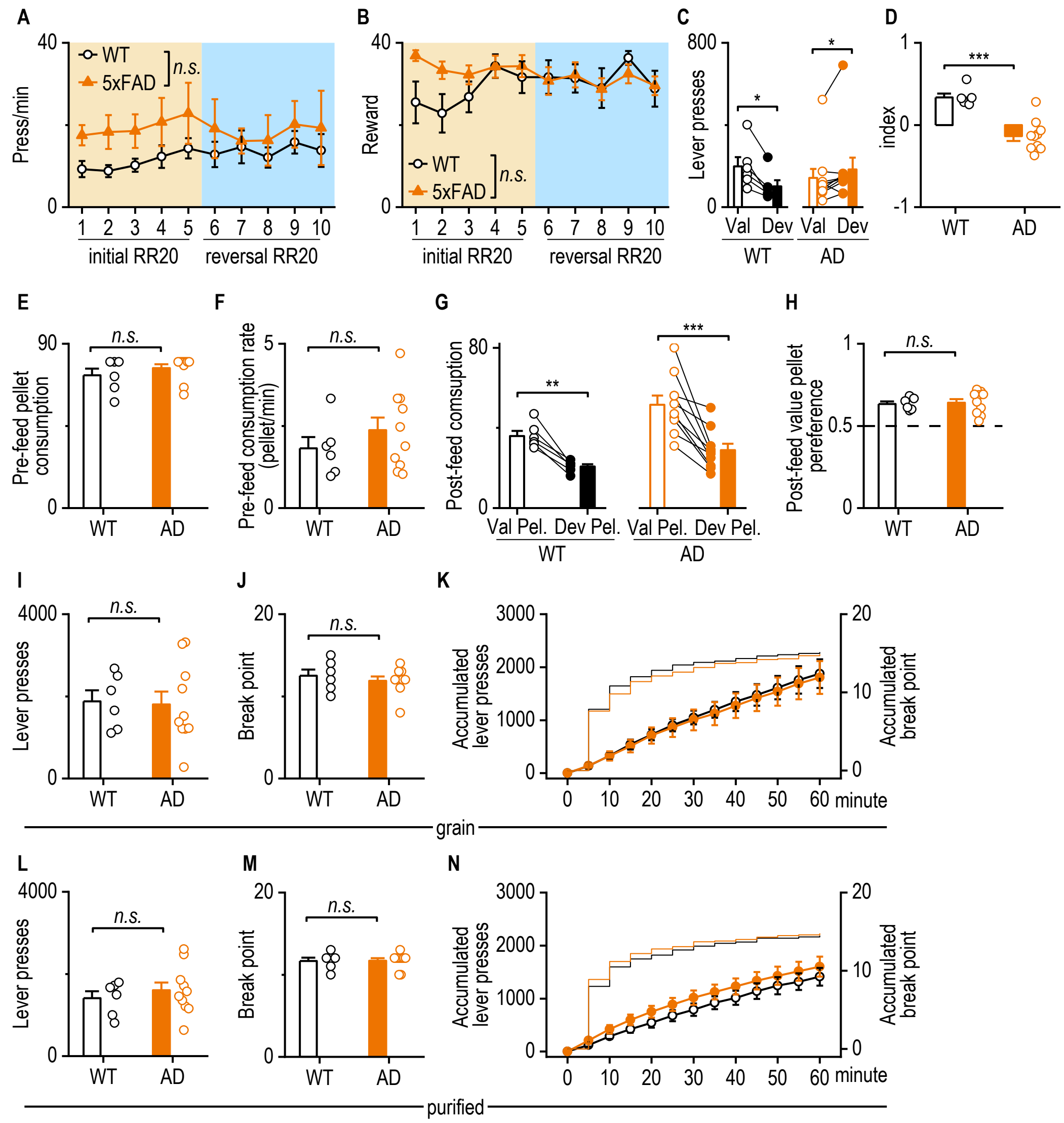

Reb_Figure 6 Reversal learning and satiety/motivation control analyses in WT and 5xFAD mice. (Same as Supplementary Figure 4)

A separate cohort of WT and 5xFAD mice was used to assess reversal learning in the absence of prior outcome devaluation and to evaluate satiety and motivational controls.

(A) Lever press rates across training during the initial RR20 and subsequent reversal RR20 sessions in WT and 5xFAD mice. No outcome devaluation or satiety manipulation was performed between the initial and reversal RR20 phases. Lever press rates did not differ between genotypes across session. Two-way RM ANOVA, genotype effect: $F_{(1,14)}$ = 0.8776, p = 0.3647. n = 6 (WT) and 10 (5xFAD).

(B) Reward collection during initial RR20 and reversal RR20 training. Two-way RM ANOVA, genotype effect: $F_{(1,14)}$, p = 0.4319. n = 6 (WT) and 10 (5xFAD).

(C) Lever presses during the reversal devaluation test. WT mice pressed the valued lever significantly more than the devalued lever whereas 5xFAD mice showed increased responding on the devalued lever relative to the valued lever. Wilcoxon matched-pairs signed-rank test, $W = -21.00$, $p = 0.0312$ for WT; $W = -40.00$, $p = 0.0391$ for 5xFAD. $^{*}p < 0.05$. n = 6 (WT) and 10 (5xFAD).

(D) Devaluation (learning) index calculated from lever presses during the reversal devaluation test. Mann–Whitney test, $U = 2$, $p = 0.0010$. $^{***}p < 0.001$. n = 6 (WT) and 10 (5xFAD).

(E) Number of pellets consumed during the 1-h pre-feed period (80 pellets total, two pellet types). Mann–Whitney test, $U = 23$, $p = 0.4268$. n = 6 (WT) and 10 (5xFAD).

(F) Pre-feed consumption rate (pellets/min) during the pre-feed period. No significant difference was observed between WT and 5xFAD mice. Unpaired t-test, $t_{14} = 0.9802$, $p = 0.3436$. n = 6 (WT) and 10 (5xFAD).

(G) Post-pre-feed free-choice consumption of non-prefed (valued pel.) and prefed (devalued pel.) pellets. Both WT and 5xFAD mice () consumed significantly more valued than devalued pellets. Paired t- test, $t_5 = 6.696$, $p = 0.0011$ for WT; $t_9 = 5.119$, $p = 0.0006$ for 5xFAD. $^{**}p < 0.01$, $^{***}p < 0.001$. n = 6 (WT) and 10 (5xFAD).

(H) Post-pre-feed valued-pellet preference index, calculated as valued pellet consumption divided by total pellet consumption. No difference was observed between genotypes. Unpaired t-test, $t_{14} = 0.2601$, $p = 0.7986$. n = 6 (WT) and 10 (5xFAD).

(I) Total lever presses during a progressive ratio (PR) test for grain pellets. Unpaired t-test, t_{14} = = 0.1544, $p = 0.8795$. n = 6 (WT) and 10 (5xFAD).

(J) Breakpoint achieved during the PR test for grain pellets. Mann–Whitney test, $U = 25.5$, $p = 0.6430$. n = 6 (WT) and 10 (5xFAD).

(K) Cumulative lever pressing across the PR session for grain pellets.

(L) Total lever presses during a PR test for purified pellets. Unpaired t-test, $t_{14} = 0.6828$, $p = 0.5059$. n = 6 (WT) and 10 (5xFAD).

(M) Breakpoint achieved during the PR test for purified pellets. Mann–Whitney test, $U = 29$, $p = 0.9685$. n = 6 (WT) and 10 (5xFAD).

(N) Cumulative lever pressing across the PR session for purified pellets.

Rev2_Maj2

ArcTRAP recombination intergrates Arc expression over several hours, meaning that labled neurons likely reflect activity occurring around the behavioral session rather than exclusively within it. Acknowledge that labeled neurons may partially reflect home-cage or post-session activation. Authors can refer to temporally precise methods (CaMPARI, Cal-Light) to validate epoch specificity.

Response: We thank the reviewer for raising this important point. We acknowledge that the ArcTRAP recombination integrates Arc expression over several hours following 4-OHT administration, and therefore the labeled neurons may partially reflect activity occurring outside the behavioral session, including home-cage or post-session activation. We have now explicitly discussed this limitation in the Discussion section (Lines 442-448). In addition, we refer to temporally precise activity-labeling approaches, such as CaMPARI and Cal-Light, which could be used in future studies to more precisely capture epoch-specific neuronal activity.

Rev2_Maj3

Targeted morphological analyses, such as quantification of dendritic spine density and classification of spine subtypes in behaviorally engaged ensembles, would complement the glutamatergic synaptic physiology data, providing complementary structural evidence for altered connectivity in the activated neuronal population.

Response: We thank the reviewer for this thoughtful and constructive suggestion. We agree that targeted morphological analyses, including dendritic spine density measurements and spine subtype classification in behaviorally engaged neuronal ensembles, could in principle provide valuable complementary structural insight into synaptic alterations.

In response to this comment, we revisited our ArcTRAP slices and acquired higher-magnification images of tdTomato-positive neurons (Reb_Figure 7, see below). However, we found that this labeling strategy does not consistently provide sufficient spatial resolution or signal-to-noise to enable reliable quantification of dendritic spines or accurate classification of spine subtypes in these neurons.

Given these technical limitations, we were unable to perform meaningful spine-level morphological analyses using the ArcTRAP mice in the current study. Nevertheless, we recognize the importance of this approach and have now explicitly discussed it as a future direction in the Discussion (Lines 449-454).

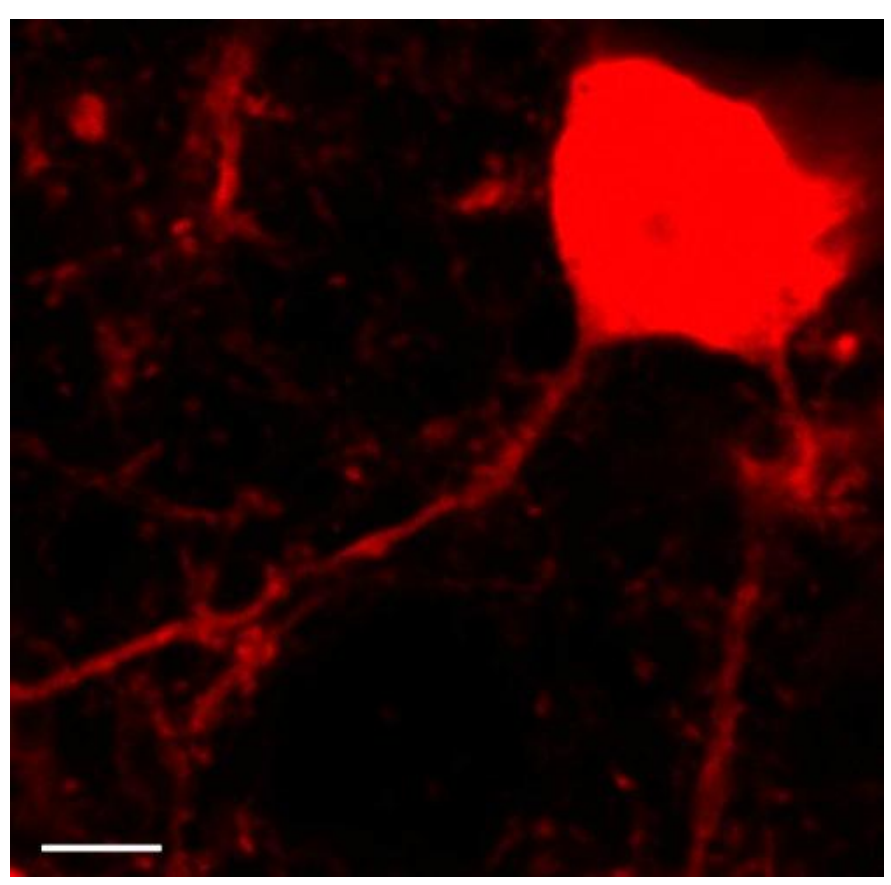

Reb_Figure 7 Representative 60x confocal image of an ArcTRAP-labeled neuron.

A tdTomato-positive neuron imaged from ArcTRAP transgenic mice under 60× confocal scanning. The soma and proximal processes are clearly visualized; however, dendritic spines are not reliably resolved at this labeling density and signal-to-noise level. Scale bar: 10 μm.

Rev2_Maj4

The authors should clarify how statistical independence was defined for all slice and cellular datasets. If multiple cells or slices were collected from the same animal, the effective biological sample size (n) must be defined as the number of animals, not cells, to avoid pseudoreplication. Mixed-effects or nested models(cells nested within animals) should be used to account for within-animal variance. Alternatively, results should be re-expressed as animal-level means and reanalyzed accordingly. This is particularly important for this review's eyes, especially in experiments with transgene(s). E.g., to ensure that genotype-dependent differences in IPSC

amplitude or input–output slope reflect unitary synaptic properties rather than recruitment differences, the authors should quantify Chrimson-tdTomato expression, infection fraction, and light-evoked spike probability versus light power in dMSNs. This control is essential to confirm that an equivalent optical drive was achieved.

Response: We thank the reviewer for raising these important points regarding statistical rigor and optogenetic controls.

Statistical independence and unit of analysis: We have now explicitly reported both the number of animals and the number of recorded cells or slices for each dataset in the corresponding figure legends. Animals were treated as the unit of analysis for behavioral and immunohistochemical experiments. For electrophysiological recordings, analyses were performed at the level of individual neurons, as the intrinsic and synaptic parameters examined (e.g., intrinsic excitability, synaptic input–output relationships, and evoked IPSC amplitudes) are defined at the single-cell level. Importantly, neurons were sampled across multiple animals in each group to minimize the influence of any single animal and to reduce animal-specific variance, thereby improving the robustness and generalizability of neuron-level measurements. This approach is consistent with recent studies and also allows comparison with similar work published recently in Nature Communications (Liu et al., 2025; Luo et al., 2025; Stagkourakis et al., 2025).

Moreover, as shown in Reb_Figure 8 below, the neuron-to-animal ratios were closely matched across control and experimental groups, demonstrating that no individual animal disproportionately contributed to the electrophysiological datasets. This balanced sampling strategy substantially mitigates concerns regarding within-animal clustering and pseudoreplication and ensures that the reported effects are not driven by over-representation of a subset of subjects.

Optogenetic controls: We fully respect the reviewer's concern that variability in viral delivery or opsin expression could contribute to the larger oIPSC amplitudes observed in AD mice. Although it is not feasible to retrospectively quantify viral expression in the same cohort used for electrophysiology, we addressed this concern by functionally assessing optical recruitment using the existing IPSC dataset.

If differences in viral expression or infection fraction were responsible for enhanced oIPSC amplitudes, they would be expected to alter presynaptic recruitment properties, including recruitment threshold (P50), saturation behavior, or the shape of the input–output relationship. We therefore quantified three complementary recruitment parameters. First, recruitment threshold (P50), defined as the light power required to evoke 50% of each neuron's maximal response, was indistinguishable between WT and AD neurons (Reb_Figure 9A). Second, the light power at which maximal recruitment (peak response) was achieved did not differ between genotypes (Reb_Figure 9B). Third, after normalizing each neuron's input–output curve to its own maximum, WT and AD neurons exhibited overlapping recruitment dynamics across light intensities (Reb_Figure 9C). Together, these analyses demonstrate that optical recruitment threshold, maximal recruitment, and recruitment dynamics are comparable between WT and AD mice, indicating that variability in viral delivery did not translate into differences in functional optical drive. Under these conditions, the enhanced oIPSC amplitudes observed in AD mice most likely reflect altered inhibitory synaptic output rather than differences in opsin expression or optical stimulation.

In addition, all surgeries were performed by the same experimenter using the same virus batch and blind to genotype. Expression time, recording window, illumination geometry, and neuron selection relative to fluorescence enrichment were tightly controlled and matched across groups, further minimizing variability in optical stimulation conditions.

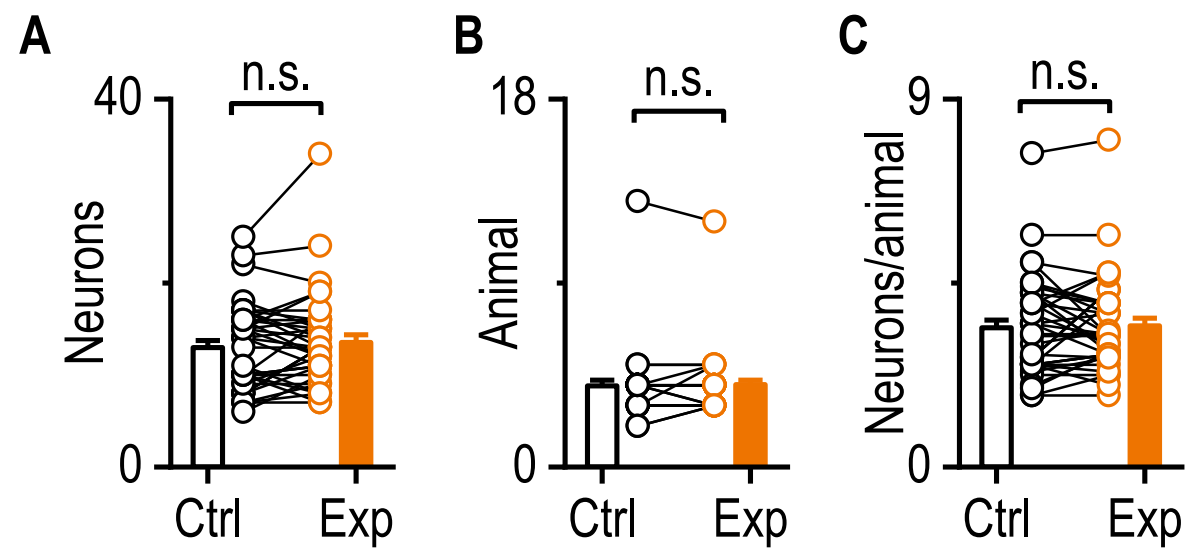

Reb_Figure 8. Comparable numbers of recorded neurons, animals, and neuron-to-animal ratios in control versus experimental groups.

Each data point represents: (A) the number of recorded neurons, (B) the number of animals used, and (C) the neuron-to-animal ratio calculated by dividing the number of recorded neurons by the number of animals. Each pair of Ctrl and Exp groups corresponds to the data for a single graph panel. n = 38 pairs of electrophysiological and live-tissue confocal imaging experiments. n.s., not significant, $p > 0.05$, paired *t*-test.

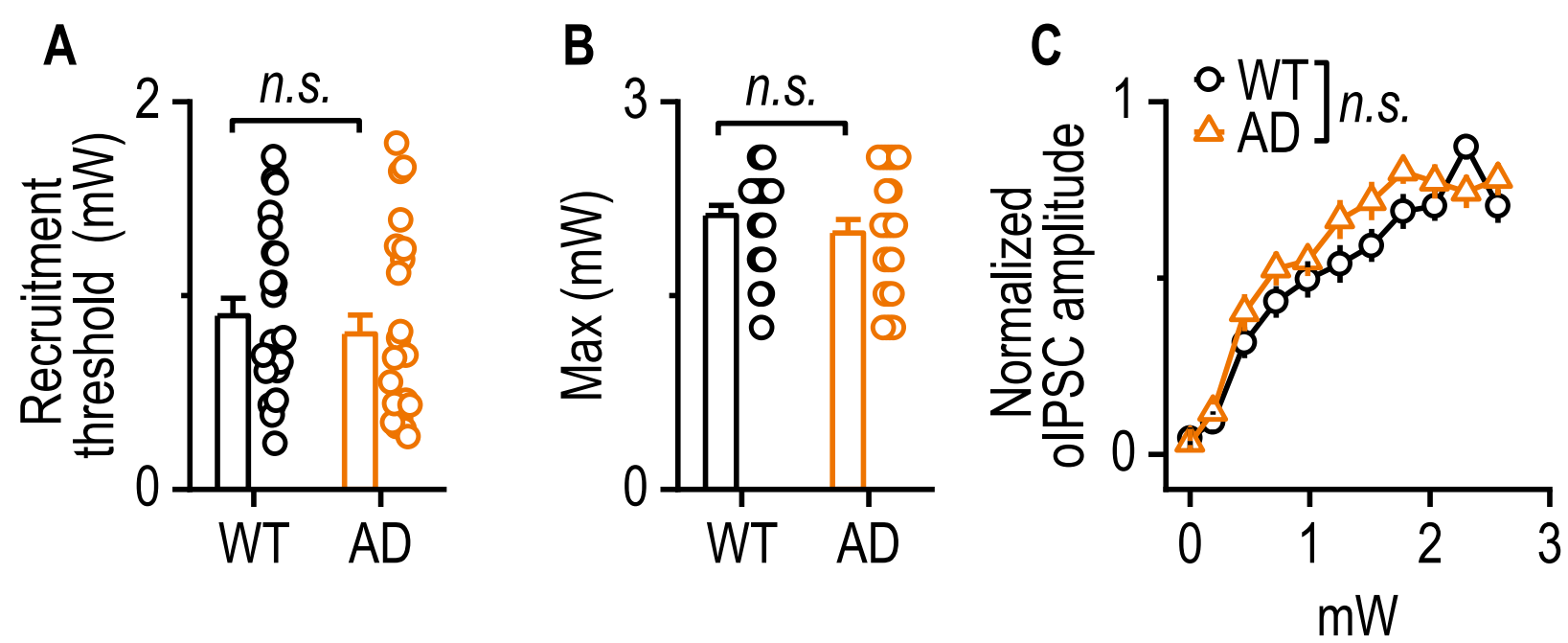

Reb_Figure 9. Functional assessment of optical recruitment equivalence between WT and AD mice.

(A) Recruitment threshold (P50), defined for each neuron as the light power at which the oIPSC amplitude first reached 50% of its own maximal (peak) response, estimated on the rising limb of the input–output curve. No significant difference was observed between WT and AD neurons.

(B) Light power at which the maximal oIPSC response (peak) was achieved for each neuron. Peak power did not differ between genotypes, indicating that full presynaptic recruitment occurred under comparable optical conditions.

(C) Input–output curves normalized to each neuron's own maximal response. After normalization, WT and AD neurons exhibited overlapping recruitment dynamics across light intensities.

Reviewer #3 (Remarks to the Author):
This study reports a deficit in instrumental, but not spatial, reversal learning in 4-month old male and female5xFAD mice This deficit associates with elevated neuronal reactivation in two regions, the medial prefrontal cortex (mPFC) and the dorsomedial striatum (DSM), involved in goal-directed behaviors. In-depth dissection of neural and circuits alterations underpinning behavioral abnormalities then reveals that the mPFC-to-DMS circuit is hyperactive in 5xFAD mice, and that this hyperactivity triggers alterations in corticostriatal and cholinergic signaling at the origin of behavioral inflexibility.
This is an ambitious study based on behavioral assays and cutting-edge neurobiological methods whose conclusions need, however, to be confirmed considering the potential biases of behavioral protocols and the in sufficient details provided in description of the experimental design.

Main points
Rev3_Maj1
Behavior
1. The reversal protocol is questionable because the mice undergo a devaluation test that alters the outcome value just before their reversal training, potentially decreasing their motivation to obtain food. Furthermore, reversal training is performed immediately in the most demanding RR20 condition, as the mice have a 0.05 probability of receiving a reward. Therefore, it is difficult to determine whether the reduction in lever presses during reversal training and the subsequent absence of post-reversal devaluation which are both selectively altered in the 5xFAD mice can be ascribed to a decrease in motivation or a deficit in behavioral flexibility. It is thus imperative to test the mice using a procedure where contingency training is followed by contingency reversal, without an intermediate post-training devaluation test.

Response: We thank the reviewer for raising this important concern regarding the potential influence of the initial devaluation test on subsequent reversal learning. We apologize for not stating our procedure clearly in the previous submission. In the original experimental design, reversal training did not occur immediately after the initial devaluation test. Instead, following the initial devaluation, mice underwent an additional initial RR20 instrumental training session before contingency reversal was introduced. This design is consistent with previously published instrumental reversal paradigms (Matamales et al., 2016; Matamales et al., 2017; Matamales et al., 2020). For clarity, these intermediate RR20 sessions were not displayed in the original figures; however, we agree that this procedural detail is important and have now explicitly described it in the revised Methods section (Lines 514-516).

Importantly, to directly address the reviewer's concern and to exclude potential motivational confounds, we conducted an additional experiment in an independent cohort in which the initial RR20 procedure was followed immediately by a reversed RR20 task, without any intervening devaluation or satiety manipulation. Under these conditions, both WT and 5xFAD mice maintained stable lever pressing and reward collection throughout reversal training (Supplementary Figure 4A–B). Despite intact performance during reversal training, 5xFAD mice

continued to show impaired post-reversal outcome devaluation (Supplementary Figure 4C–D), consistent with the deficits reported in the main manuscript. We thank the reviewer for this insightful question, which helped us improve the rigor of our manuscript.

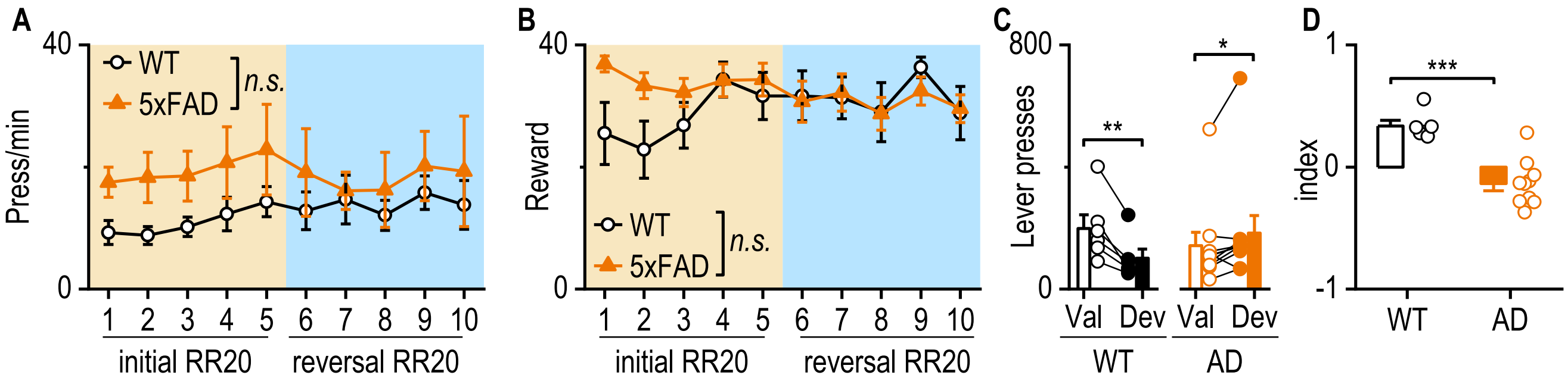

Supplementary Figure 4. Reversal learning and satiety/motivation control analyses in WT and 5xFAD mice.

A separate cohort of WT and 5xFAD mice was used to assess reversal learning in the absence of prior outcome devaluation and to evaluate satiety and motivational controls.

(A) Lever press rates across training during the initial RR20 and subsequent reversal RR20 sessions in WT and 5xFAD mice. No outcome devaluation or satiety manipulation was performed between the initial and reversal RR20 phases. Lever press rates did not differ between genotypes across sessions. Two-way RM ANOVA, genotype effect: $F_{(1,14)}$ = 0.8776, p = 0.3647. n = 6 (WT) and 10 (5xFAD).

(B) Reward collection during initial RR20 and reversal RR20 training. Two-way RM ANOVA, genotype effect: $F_{(1,14)}$, p = 0.4319. n = 6 (WT) and 10 (5xFAD).

(C) Lever presses during the reversal devaluation test. WT mice pressed the valued lever significantly more than the devalued lever, whereas 5xFAD mice showed increased responding on the devalued lever relative to the valued lever. Wilcoxon matched-pairs signed-rank test, $W = -21.00$, p = 0.0312 for WT; $W = -40.00$, p = 0.0391 for 5xFAD. $^{*}p < 0.05$. n = 6 (WT) and 10 (5xFAD).

(D) Devaluation (learning) index calculated from lever presses during the reversal devaluation test. Mann–Whitney test, U = 2, p = 0.0010. $^{***}p < 0.001$. n = 6 (WT) and 10 (5xFAD).

Rev3_Maj2

2. The Barnes test, used to demonstrate that spatial learning and reversal are intact in 4-month-old 5xFADmice, does not involve food rewards, is less effortful, and much simpler, in terms of amount of information to process, than reversal training performed under RR20 conditions. It has been suggested that the medial prefrontal cortex (mPFC) is involved in reversal learning only when the task becomes more complex and demands a higher level of attention (Izquierdo et al., Neuroscience, 2017,45:12–2). The use of an alternative task (e.g., a radial maze with different subsets of baited arms during training and reversal) is needed to determine whether the reversal deficit observed in 5xFAD mice is specific to goal-directed (and not spatial) tasks, or specific to complex tasks, independent of goal-directed or spatial contingencies.

Response: We thank the reviewer for this insightful comment regarding task complexity and the engagement of medial prefrontal cortex (mPFC)–dependent mechanisms in reversal learning. We fully agree that the Barnes maze is a relatively simple spatial task that does not involve food

reward, instrumental action–outcome contingencies, or high attentional demand. Accordingly, we do not equate intact performance in the Barnes maze with intact reversal learning in more complex or goal-directed tasks. In the original manuscript, the Barnes maze was included solely to assess basic spatial learning and spatial reversal, rather than higher-order cognitive flexibility.

Following the reviewer's suggestion, we performed an additional rewarded spatial reversal task using an eight-arm radial maze in an independent cohort of 3–4-month-old WT and 5xFAD mice. This paradigm incorporates food reward and requires learning and updating of multiple spatial contingencies, thereby increasing task complexity and attentional demand relative to the Barnes maze while remaining primarily spatial in nature. Under these conditions, WT and 5xFAD mice exhibited comparable performance during both initial learning and reversal learning, as assessed by time spent in rewarded arms, suppression of non-rewarded and control arm exploration, and reward collection across sessions (Reb_Figure 10). Thus, we did not detect impairments in spatial learning or spatial reversal in early-stage 5xFAD mice, even in a rewarded and more demanding spatial task. We have now added this to the Results section (Lines 102-108) and Supplementary Figure.

Importantly, these findings do not argue against a deficit in cognitive flexibility per se, but instead indicate that cognitive flexibility is differentially affected across behavioral domains. Cognitive flexibility is a multifaceted construct that can be expressed through distinct behavioral modalities, including spatial flexibility and goal-directed, action–outcome–based flexibility. While early-stage 5xFAD mice retain intact spatial flexibility in both non-rewarded (Barnes maze) and rewarded (radial arm maze) spatial reversal tasks, they exhibit robust impairments in a probabilistic, high-effort instrumental reversal task, which places strong demands on action–outcome monitoring, attentional control, and corticostriatal circuitry.

Accordingly, we have revised the manuscript to narrow and clarify our conclusions. We now describe the behavioral phenotype in 5xFAD mice as a selective impairment in cognitive flexibility expressed in the instrumental, goal-directed domain, rather than a generalized deficit across all forms of reversal learning. The Results section has been revised to focus specifically on instrumental reversal learning while avoiding broader claims regarding spatial flexibility. We thank the reviewer for raising this concern, which improved the accuracy of our interpretation of the results.

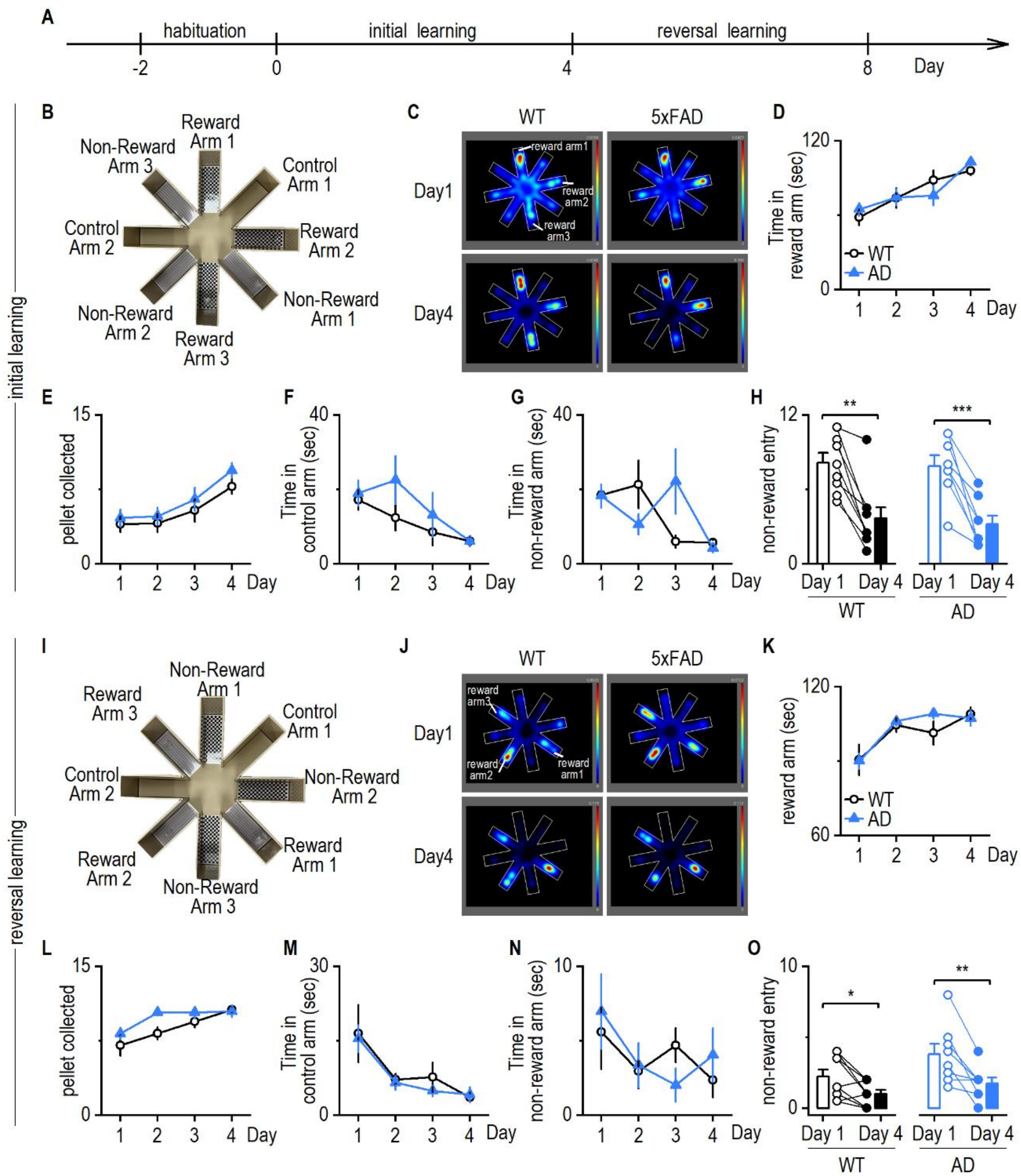

Reb_Figure 10. 5xFAD mice did not exhibit deficits in reversal learning in a rewarded eight-arm radial maze at 3 months of age. (Same as Supplementary Figure 1)

A, Experimental timeline of the rewarded eight-arm radial maze task, including habituation, initial learning, and reversal learning phases.

B, Schematic of the maze configuration during the initial learning phase. Three arms were baited with food reward (reward arms), three arms were designated as non-reward arms, and two arms served as control arms.

C, Representative heat maps illustrating arm exploration patterns of WT and 5xFAD mice on Day 1 and Day 4 of the initial learning phase.

D–G, Quantification of behavioral performance during the initial learning phase, including time spent in reward arms (D), number of pellets collected (E), time spent in control arms (F), and time spent in non-reward arms (G). Two-way RM ANOVA, genotype comparison: $F_{(1,15)}$ = 0.0061, p = 0.9390 for D; $F_{(1,15)}$ = 0.7323, p = 0.4056 for E; $F_{(1,15)}$ = 1.010, p = 0.3310 for F; $F_{(1,15)}$ = 0.1114, p = 0.7432 for G. n = 9 (WT) and 8 (5xFAD).

H, Comparison of time spent in non-reward arms between Day 1 and Day 4 during the initial learning phase. Both WT mice and 5xFAD mice showed a significant reduction. Wilcoxon matched-pairs signed-rank test for WT, W = −45.00, p = 0.0039; paired t-test for 5xFAD, t_7 = 5.693, p = 0.0007. $^{**}p < 0.01$, $^{***}p < 0.001$. n = 9 (WT) and 8 (5xFAD).

I, Schematic of the maze configuration during the reversal learning phase, in which reward and non-reward arm identities were reassigned.

J, Representative heat maps showing arm exploration patterns of WT and 5xFAD mice on Day 1 and Day 4 of the reversal learning phase.

K–N, Quantification of behavioral performance during the reversal learning phase, including time spent in reward arms (K), number of pellets collected (L), time spent in control arms (M), and time spent in non-reward arms (N). Two-way RM ANOVA, genotype comparison: $F_{(1,15)}$ = 0.2265, p = 0.6410 for K; $F_{(1,15)}$ = 2.142, p = 0.1639 for L; $F_{(1,15)}$ = 0.0113, p = 0.9166 for M; $F_{(1,15)}$ = 0.1445, p = 0.7092 for O. n = 9 (WT) and 8 (5xFAD).

O, Comparison of time spent in non-reward arms between Day 1 and Day 4 during the reversal learning phase. Both WT and 5xFAD mice showed significant reductions. Wilcoxon matched-pairs signed-rank test for WT, W = −37.00, p = 0.0312; paired t-test for 5xFAD, t_7 = 3.667, p = 0.0080. $^{*}p < 0.05$, $^{**}p < 0.01$. n = 9 (WT) and 8 (5xFAD).

Rev3_Maj3

mPFC subregions regions

Numerous data in humans and rodents indicate a functional specificity of subregions of the medial prefrontal cortex, with differential involvement of the prelimbic, infralimbic, and orbitofrontal cortices in various functions, including reward and punishment, extinction, working memory, attention, value-based decision-making, goal-oriented behavior, and reversal learning. This aspect is not considered in the present study, despite its importance for refining the interpretation of the data. According to the mouse brain atlas by Paxinos and Franklin, the reported stereotaxic coordinates of the virus injection site (AP: 1.94 mm, ML: ±0.35 mm, DV: -2.20 mm) should target the PL and IL regions of the anterior part of the medial prefrontal cortex, suggesting that neurobiological measurements were performed in these regions. The authors should specify the location of their observations and manipulations and adapt the interpretation of their data accordingly.

Response: We thank the reviewer for raising this important point regarding the functional heterogeneity of medial prefrontal cortex (mPFC) subregions. We agree that the prelimbic (PL), infralimbic (IL), and orbitofrontal cortices have distinct and sometimes dissociable roles in reward processing, goal-directed behavior, and reversal learning.

Based on our stereotaxic coordinates (AP +1.94 mm, ML ±0.35 mm, DV −2.20 mm, relative to bregma) and post hoc histological verification, viral expression and subsequent recordings/imaging were largely confined to the anterior PL and IL regions of the mPFC, with minimal spread to adjacent areas and no involvement of the orbitofrontal cortex. Accordingly,

the neurobiological measurements reported in this study primarily reflect activity within these PL/IL subregions.

We have now clarified this point in the Methods (Line 667) and have refined the interpretation in the result section (Lines 358-360) to explicitly frame our findings in the context of PL/IL mPFC circuits, rather than the mPFC as a whole. Importantly, our conclusions are therefore restricted to these anterior mPFC subregions and their role in neuronal recruitment and behavioral flexibility during reversal learning.

Rev3_Maj4

Supplementary data

Paradoxically, this section includes more experiments than the main part, with some experiments being asmuch crucial as those of the main part. In particular, findinggs reported in Suppl. Figures 6,7,8,9,10,11)provide evidence that correcting hyperexcitability in abnormally activated regions and circuits of 5xFAD mice is sufficient to rescue behavioral and neuronal alterations, including the reduction of Abeta levels and the normalization of acetylcholine levels. This paradox definitely raises the question of the reorganization of the manuscript. One option would be to present in the main part the "dysfunctions" and their "rescue" in the same figures, and to remove in the supplementary data the experiments (pilocarpine, object novelty, investigations performed at 12 months) which are indirectly related to the central topic of the study(identification of an early deficit in 4-month old 5xFAD mice

Response: We thank the reviewer for this comment and carefully considered the organization of the manuscript and Supplementary Data. While several supplementary experiments (Suppl. Figures 6 and 7, now Suppl. Figures 8 and 10) provide important supporting evidence, we believe they are complementary rather than essential to the central narrative, which focuses on early corticostriatal circuit dysfunction in 4-month-old 5xFAD mice. Moreover, the general mPFC manipulation experiments (Suppl. Figures 8, 9, and 11; now Suppl. Figures 11, 12, and 14) produced effects similar to those observed with circuit-specific suppression but lacked projection specificity and are therefore presented as supportive data. The object novelty task (Suppl. Figure 10, now Suppl. Figure 13), although not part of the primary behavioral framework, is a widely used measure of cognitive function and provides additional evidence for behavioral improvement following circuit manipulation. The pilocarpine experiments offer independent and clinically relevant evidence of global network hyperexcitability, and the 12-month experiments demonstrate that corticostriatal hyperactivity persists into later disease stages.

Together, these supplementary experiments strengthen the overall conclusions without altering the main focus of the study. Our primary storyline focuses on the corticostriatal circuit in early AD, and the supplementary experiments support aspects of the main findings but are not required to establish the core conclusion. We therefore prefer to keep the main figures and central narrative concise and focused. Nevertheless, we fully respect the editor's judgment and are willing to further streamline the manuscript if recommended.

Rev3_Maj5

Methods

1. Experiments design

Experiments expected to link behavioral impairments to neurobiological alterations (e.g. electrophysiology)were carried out at age points which do not exactly coincide. Reversal learning: 3, 6, 12 months; Electrophysiology: 4 and 5 months; Pathway studies: 4 and 12 months; Seizures and EEG: not indicated. Maybe a diagram or a timeline specifying the ages of mice at each experimental phase might help to distinguish between neurobiological causal alterations and their long term consequences.

Response: We thank the reviewer for this helpful suggestion. We agree that clearly specifying the age at which each experiment was performed is important for distinguishing early neurobiological alterations from their longer-term consequences. We have now explicitly indicated the age of the animals tested for each experiment in both the figure legends (e.g., Lines 1088, 1150) and the Results section (e.g., Lines 73, 173, 195, 239) to improve clarity and facilitate comparison across experimental approaches.

Rev3_Maj6

2. Administration of 4-OHT with isoflurane

It is reported that "The 4-OHT was administered immediately following either the completion of the middle two initial RR20 sessions or after the reversal RR20 sessions. Mice were first introduced to an isoflurane chamber immediately after the operant session, and 4-OHT was injected i.p. The use of isoflurane is to reduce the pain and discomfort due to the 4-OHT/oil mix injection."

This sentence should be rewritten. It is unclear whether mice were anesthetized immediately after, or during, or before behavioral assessment thereby preventing to estimate anesthesia interference with performance or memory. Moreover, the justification for administering isofluorane before 4-OHT injections sounds a bit inconsistent with the fact that the 30-day i.p. injections of Compound 21 were administered in awake mice.

Response: We thank the reviewer for raising this important point. We have revised the description of the 4-OHT administration procedure to clearly specify the timing of isoflurane exposure relative to behavioral testing (Lines 546–551). Specifically, mice completed the behavioral session fully awake. Isoflurane anesthesia was applied only after completion of the session and immediately prior to the intraperitoneal injection of 4-OHT, solely to minimize pain and distress associated with injection of the oil-based solution. Thus, isoflurane exposure did not overlap with behavioral performance or memory assessment.

With respect to Compound 21 (C21) administration, isoflurane was not used and injections were performed in awake mice. C21 was dissolved in saline and does not cause substantial discomfort upon intraperitoneal injection, and therefore anesthesia was not required. In addition, C21 injections were conducted at least 6 hours after behavioral training to avoid potential interference with memory consolidation after behavior training. C21-mediated inhibition of cortical neurons was intended as a sustained, daily modulation of neuronal activity rather than an acute post-training manipulation.

Importantly, the C21-treated mice constituted a separate experimental cohort from those receiving 4-OHT, as these experiments were designed to address distinct mechanistic questions. We have clarified these procedural differences in the Methods section to avoid confusion.

Rev3_Maj7

3. Off-target potential effects of Compound 21 injections

There is evidence that, at high doses, Compound-21 also binds to endogenous ACh receptors with consequent increase in available and detectable acetylcholine (Jendrila M et al, Sci Rep 201, 9:4522). The range of the injected dose should be verified to discard this off-target effect.

Response: We thank the reviewer for this insightful comment and agree that verifying the specificity of Compound-21 (C21) is essential for the interpretation of chemogenetic data.

Methodological limitation: Radioligand binding assays, as used by Jendryka et al. (2019) to identify off-target receptor interactions, represent a **direct** biochemical approach to assess receptor binding. However, this methodology requires specialized infrastructure that is not available in our laboratory, and we are therefore unable to directly replicate these assays.

Verification of injected dose range: In Jendryka et al. (Reb_Figure 11), off-target binding of C21 to muscarinic receptors (M1–M3) was detected in an in vitro screening assay at a concentration of **10 μM** (10,000 nM). Pharmacokinetic data from the same study show that a 3 mg/kg systemic dose of C21 results in a peak free-drug CSF concentration of approximately **52 nM**. By linear extrapolation, the **1 mg/kg** dose used in our study is expected to yield **~17 nM** free-drug concentration in the CSF, which is approximately **580-fold lower** than the concentration (10 μM) required to detect off-target binding in vitro. Notably, Jendryka et al. conclude that even doses up to 10 mg/kg are unlikely to produce meaningful off-target behavioral effects in vivo.

Behavioral verification in non-DREADD controls: As emphasized in the field, the most relevant empirical safeguard against off-target effects is the inclusion of non-DREADD-expressing control animals. Consistent with this principle, C21 administration at 1 mg/kg did not induce behavioral changes in animals lacking DREADD expression in our study (Figure 7D, mCherry group did not learn reversal learning). This is in agreement with prior work by Tran et al. (2020) demonstrating that both acute (0.3–3 mg/kg) and chronic (1 mg/kg, repeated administration for 16 weeks) systemic C21 injections do not alter locomotion, exploration, anxiety-like behavior, or depression-related behaviors in non-DREADD-expressing mice even after C21 is not onboard. Together with the findings of Jendryka et al., these results support the recommendation that doses in the range of 0.4–1.0 mg/kg effectively activate DREADDs while avoiding nonspecific behavioral effects, as other accepted manuscripts at Nature Communications also use 1 mg/kg C21(Baker et al., 2023; Koutlas et al., 2025; Wang et al., 2025).

In summary, although we cannot directly perform radioligand binding assays, we have verified our injected dose range against established pharmacokinetic and behavioral benchmarks in the literature. The low predicted brain concentrations achieved in our study, combined with the absence of behavioral effects in non-DREADD-expressing control animals, strongly support the conclusion that the observed effects are DREADD-specific rather than due to off-target cholinergic modulation.

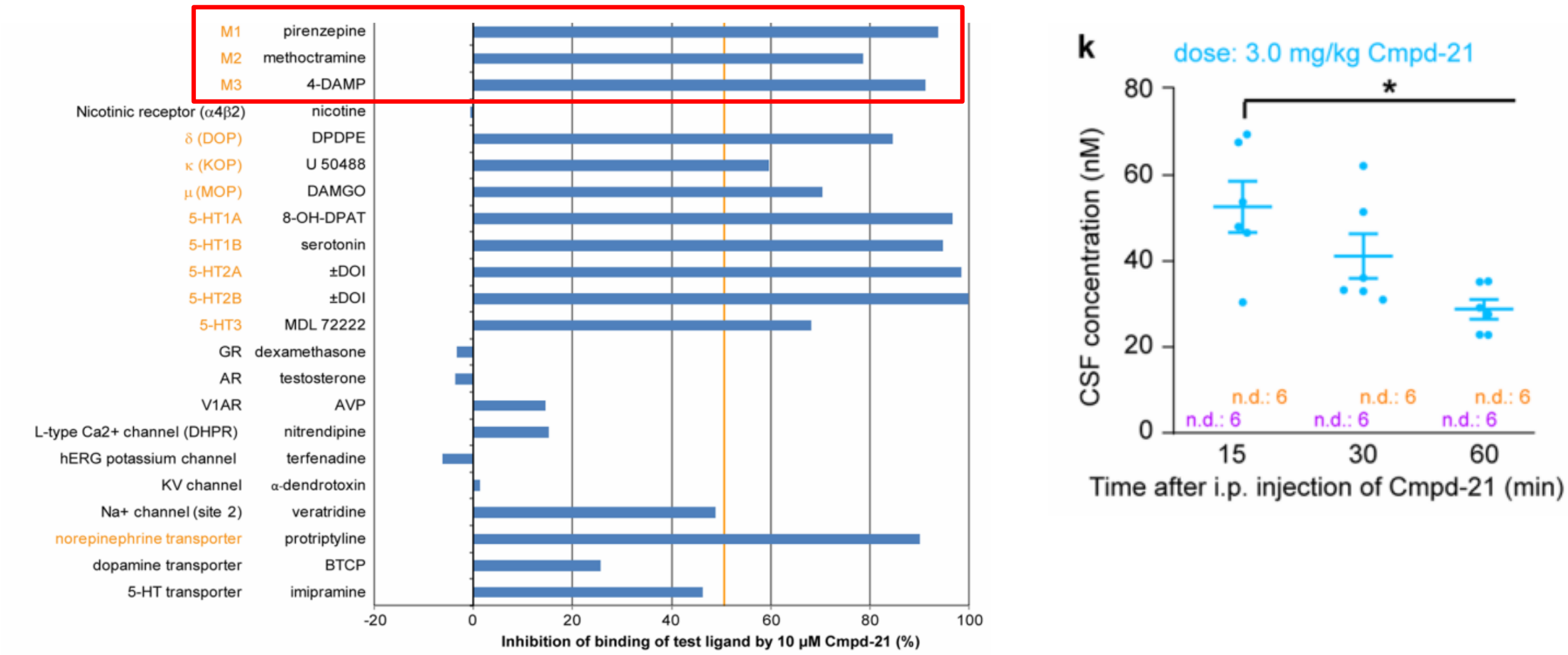

Reb_Figure 11. Off-target binding and CSF exposure of Compound 21 (C21). Adapted from Jendryka (2019).

In vitro screening detected off-target binding of C21 to muscarinic receptors (M1–M3) only at 10 μM (10,000 nM) (left). Pharmacokinetic data from the same study show that a 3 mg/kg systemic dose yields a peak free-drug CSF concentration of ~52 nM (Right).

Rev3_Maj8

4. Hippocampal data

Images provided in Suppl Fig3 show that trapped dentate gyrus neurons do not differ between WT and5xFAD in either initial or reversal learning This observation is in contrast with the report that hippocampal neurogenesis contribute to cognitive flexibility (Anacker & Hen, 2017 Nat Rev Neurosci 18, 335–346. This study should be cited and commented.

Response: We thank the reviewer for raising this apparent discrepancy. We fully agree that the hippocampus plays an important role in cognitive flexibility under many conditions, including in Alzheimer's disease, and that hippocampal neurogenesis has been implicated in this process, as discussed by Anacker and Hen (2017).

We have now added a discussion of this point to the Discussion section (Lines 388-392), explicitly acknowledging the limitations of our activity-trapping approach, the task dependency of hippocampal involvement, and the fact that our findings do not exclude an important role for hippocampal neurogenesis in cognitive flexibility under other conditions or paradigms.

Rev3_Maj9

5.Statistical analyses

Two-way ANOVA with genotype and sessions comparing RR20 performance between contingency training and contingency reversal, or comparing Val vs Dev scores between post-training and post-reversal devaluation tests the different ages mice were tested would have assessed more accurately inter-strain differences in behavior.

Response: We thank the reviewer for this helpful suggestion. In accordance with the recommendation, we directly compared RR20 performance between initial contingency training and contingency reversal using a two-way RM ANOVA with Genotype and Session as factors. This analysis revealed a significant Session × Genotype interaction, indicating that lever-press rates increased across sessions in WT mice but remained relatively stable in 5xFAD mice (Reb_Fig. 12A).

With respect to outcome devaluation, this comparison is presented in Fig. 1G and is reproduced here as Reb_Fig. 12B. Devaluation performance was quantified using a devaluation index ((Valued − Devalued)/(Valued + Devalued)). A two-way repeated-measures ANOVA with Genotype and Test Session (post-training vs. post-reversal) revealed a significant Genotype × Session interaction, demonstrating a selective impairment in outcome devaluation in 5xFAD mice following reversal learning.

Age was not included as an additional factor because the primary focus of the study is early-stage Alzheimer's disease, and all experiments were conducted at a single early disease time point. Behavioral data from older cohorts were not designed for systematic cross-age comparisons and were therefore not included in these analyses.

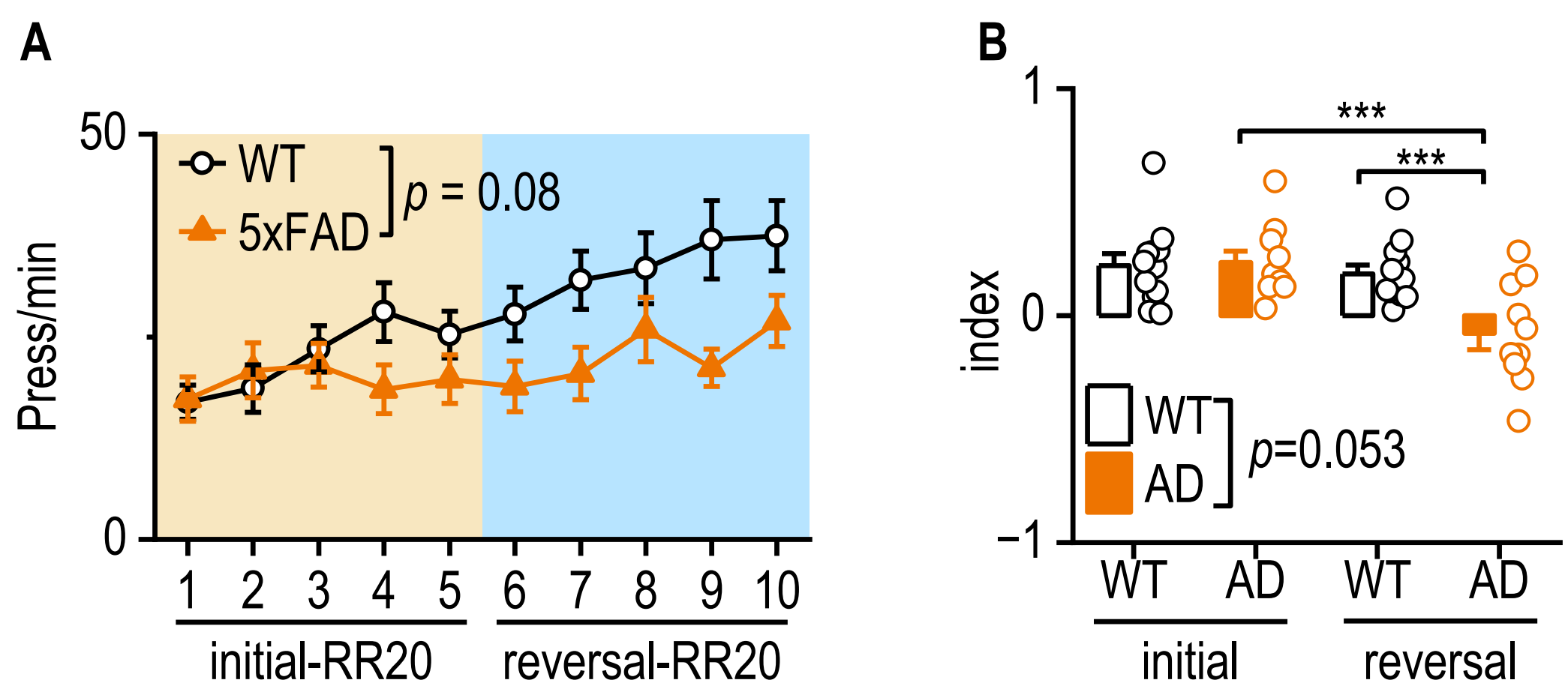

Reb_Figure 12. Instrumental performance and outcome devaluation index during initial and reversal learning.

A, Lever-press rates during initial RR20 (sessions 1–5) and reversal RR20 (sessions 6–10) training in WT and 5xFAD mice. Two-way RM ANOVA, session x genotype: $F_{(9,\ 180)} = 3.155$, $p = 0.0015$; genotype $F_{(1,\ 20)} = 3.325$, $p = 0.0832$. Data are adapted from Fig. 1C and Fig. 1E.

B, 5xFAD mice exhibited a significantly lower reversal index than their initial devaluation index as well as the reversal devaluation index of WT mice. The index is calculated as (Val-Dev)/(Val + Dev). Two-way RM ANOVA, genotype: $F_{(1,20)} = 4.216$, $p = 0.053$; interaction: $F_{(1,20)} = 8.518$, $p = 0.0085$; Sidak's multiple comparisons post-hoc analysis, 5xFAD initial vs reversal: t = 4.49, $p < 0.001$; 5xFAD reversal vs WT reversal: t = 0.341, $p = 0.002$. *** $p < 0.001$. Data are the same as shown in Fig. 1G

Rev3_Maj10

Theoretical framework

The authors alternately interpret the behavioral deficit in 5xFAD mice as an alteration in goal-oriented behavior or an alteration in cognitive flexibility. While both of these executive functions require the modification of previously learned responses, goal-oriented systems involve a specific sensitivity to the value of the outcome, which generates the action necessary to reach it. It is worth noting that outcome devaluation is considered one of the best methods for identifying goal-oriented behavior in rodents (Pierce-Messick et al., Nat Protoc. 2025, 20:518-538). Thus, the fact that 4-month old WT and 5xFAD mice exhibit significantly less lever presses for devalued outcomes (Dev) than for valued outcomes (Val) in the post-training devaluation test reveals that the mutant mice do exhibit goal-oriented behavior. This point should be clearly stated in the discussion. Indeed, the fact that they do not show devaluation after reversal training suggests the existence of experimental factors that overall abolish their prior ability to devaluate.

Response: We thank the reviewer for this insightful and important comment. We agree that outcome devaluation is a well-established assay for assessing goal-directed behavior, as described by Pierce-Messick et al. (2025). Consistent with this framework, both WT and 5xFAD mice at 4 months of age exhibited significantly reduced responding for devalued compared to valued outcomes in the post-training devaluation test, indicating intact goal-directed behavior at baseline. Importantly, although 5xFAD mice acquired the initial action–outcome contingency normally during instrumental training, they exhibited marked impairments during the reversal phase, suggesting a deficit in updating previously learned action–outcome associations rather than a primary impairment in goal-directed control.

The Discussion (Lines 380–384) has been revised to explicitly reflect this distinction and to ensure consistent terminology throughout.

Rev4_Con1

Reviewer #4 (Remarks to the Author):

Cognitive flexibility deficits emerge early in Alzheimer's disease (AD), yet their circuit basis is unclear. In5xFAD mice, the authors found early reversal learning impairments linked to hyperactivity in the medial prefrontal cortex (mPFC) and dorsomedial striatum (DMS). mPFC neurons and DMS direct pathway neurons were hyperexcitable, increasing inhibition of cholinergic interneurons and reducing striatal acetylcholine release. Chemogenetic suppression of the mPFC–DMS circuit normalized neurotransmission, lowered cortical Aβ, and rescued learning deficits, revealing a hyperactive corticostriatal pathway as a driverof cognitive inflexibility in early AD. Experiments were well executed. The manuscript is well written and highlights interesting features in excitability underlying changes in cognitive flexibility in the 5xFAD mouse at early stages of disease progression. However, although the phenomenon is very interesting, the manuscript lacks an investigation of the molecular bases underlying changes in excitability that one would have expected for a thorough investigation of the phenomenon.

Response: We thank the reviewer for this thoughtful suggestion. We agree that identifying the molecular determinants of neuronal hyperexcitability in early AD would be highly valuable,

particularly for translational applications, and we have added this perspective to the Discussion (lines 448–454).

The primary goal of the present study, however, was to define the circuit-level mechanism by which early corticostriatal hyperactivity drives cognitive inflexibility in 5xFAD mice. Using convergent approaches—including cell-type–specific electrophysiology, chemogenetic suppression of the mPFC→DMS pathway, acetylcholine sensor measurements, and Aβ pathology analyses, we provide causal evidence linking a hyperactive corticostriatal circuit to behavioral deficits. A comprehensive investigation of upstream molecular mechanisms (e.g., ion channel regulation, receptor composition, or intracellular signaling) would require another dedicated and systematic study.

We therefore view such analyses as an important future direction that can build upon and extend the circuit framework established here.

Rev5_Con1
Reviewer #5 (Remarks to the Author):
I co-reviewed this manuscript with one of the reviewers who provided the listed reports. This is part of the Nature Communications initiative to facilitate training in peer review and to provide appropriate recognition for Early Career Researchers who co-review manuscripts.

Response: We thank the reviewer for their careful evaluation of the manuscript and for the constructive comments provided in the reports.

References

Baker M, Kang S, Hong SI, Song M, Yang MA, Peyton L, Essa H, Lee SW, Choi DS (2023) External globus pallidus input to the dorsal striatum regulates habitual seeking behavior in male mice. Nat Commun 14:4085.

Bero AW, Yan P, Roh JH, Cirrito JR, Stewart FR, Raichle ME, Lee JM, Holtzman DM (2011) Neuronal activity regulates the regional vulnerability to amyloid-beta deposition. Nat Neurosci 14:750–756.

Golden LR et al. (2025) APOE4 to APOE2 allelic switching in mice improves Alzheimer's disease-related metabolic signatures, neuropathology and cognition. Nat Neurosci 28:2461–2475.

Huynh TV, Liao F, Francis CM, Robinson GO, Serrano JR, Jiang H, Roh J, Finn MB, Sullivan PM, Esparza TJ, Stewart FR, Mahan TE, Ulrich JD, Cole T, Holtzman DM (2017) Age-Dependent Effects of apoE Reduction Using Antisense Oligonucleotides in a Model of beta-amyloidosis. Neuron 96:1013–1023 e1014.

Jendryka M, Palchaudhuri M, Ursu D, van der Veen B, Liss B, Katzel D, Nissen W, Pekcec A (2019) Pharmacokinetic and pharmacodynamic actions of clozapine-N-oxide, clozapine, and compound 21 in DREADD-based chemogenetics in mice. Sci Rep 9:4522.

Koutlas I, Patrikiou L, van der Starre SE, Danko D, Wolterink-Donselaar IG, Luijendijk MCM, Adan RAH, Meye FJ (2025) Distinct ventral tegmental area neuronal ensembles are indispensable for reward-driven approach and stress-driven avoidance behaviors. Nat Commun 16:3147.

Liu Y et al. (2025) Prolonged exposure to food odors suppresses feeding via an olfactory bulb-to-hypothalamus circuit. Nat Commun 16:7892.

Luo Z, Chen J, Liu Y, Dai Y, Gao H, Zhang B, Ou H, So KF, Wei JA, Zhang L (2025) Treadmill exercise prevents stress-induced anxiety-like behaviors via enhancing the excitatory input from the primary motor cortex to the thalamocortical circuit. Nat Commun 16:939.

Matamales M, Skrbis Z, Hatch RJ, Balleine BW, Gotz J, Bertran-Gonzalez J (2016) Aging-Related Dysfunction of Striatal Cholinergic Interneurons Produces Conflict in Action Selection. Neuron 90:362–373.

Matamales M, McGovern AE, Mi JD, Mazzone SB, Balleine BW, Bertran-Gonzalez J (2020) Local D2- to D1-neuron transmodulation updates goal-directed learning in the striatum. Science 367:549–555.

Matamales M, Skrbis Z, Bailey MR, Balsam PD, Balleine BW, Gotz J, Bertran-Gonzalez J (2017) A corticostriatal deficit promotes temporal distortion of automatic action in ageing. Elife 6:e29908.

Oblak AL et al. (2021) Comprehensive Evaluation of the 5XFAD Mouse Model for Preclinical Testing Applications: A MODEL-AD Study. Front Aging Neurosci 13:713726.

Stagkourakis S, Williams P, Spigolon G, Khanal S, Ziegler K, Heikkinen L, Fisone G, Broberger C (2025) Maternal aggression driven by the transient mobilisation of a dormant hormone-sensitive circuit. Nat Commun 16:8553.

Tran FH, Spears SL, Ahn KJ, Eisch AJ, Yun S (2020) Does chronic systemic injection of the DREADD agonists clozapine-N-oxide or Compound 21 change behavior relevant to locomotion, exploration, anxiety, and depression in male non-DREADD-expressing mice? Neurosci Lett 739:135432.

Wang Y, Yue S, Yang F, Chen L, Singh A, Marciniak M, Wei W, Wang ZJ (2025) Prelimbic cortex to ventral tegmental area projection regulates early social isolation stress-potentiated heroin seeking in mice. Nat Commun 16:9541.

Wasen C, Beauchamp LC, Vincentini J, Li S, LeServe DS, Gauthier C, Lopes JR, Moreira TG, Ekwudo MN, Yin Z, da Silva P, Krishnan RK, Butovsky O, Cox LM, Weiner HL (2024) Bacteroidota inhibit microglia clearance of amyloid-beta and promote plaque deposition in Alzheimer's disease mouse models. Nat Commun 15:3872.

Point-by-point response to reviewers' concerns

Reviewer #1 (Remarks to the Author):

Reviewer 1, Concern 1 (Rev1_Con1)

This is an ambitious and potentially important research paper. The authors combine behavior, activity-dependent tagging, ex vivo electrophysiology, acetylcholine sensor imaging, and chemogenetic circuit inhibition to argue that early mPFC→DMS hyperactivity in 5xFAD mice suppresses striatal cholinergic function and drives early cognitive inflexibility. The study is conceptually strong and the overall model is interesting, especially the idea that executive dysfunction precedes overt spatial memory deficits and is linked to corticostriatal rather than hippocampal dysfunction at this stage. However, several central claims are currently stated more strongly than the data justify, and the manuscript would benefit from tighter statistical/reporting standards and a more careful treatment of causality.

The abstract is clear and impactful, but the causal language is too strong in its current form. The data support a model in which pathological corticostriatal hyperactivity is associated with, and likely contributes to, cholinergic dysfunction and reversal-learning deficits. However, the manuscript does not yet directly demonstrate that the dMSN→CIN→ACh node is itself necessary or sufficient for the behavioral phenotype. I would recommend softening phrases such as "disrupts cholinergic signaling and drives early cognitive inflexibility," unless the authors add a more direct mechanistic rescue at the CIN/ACh level.

Response: We thank the reviewer for this insightful and important comment. We agree that the original manuscript did not sufficiently distinguish between circuit-level causality and downstream mechanistic association, particularly in the way cholinergic changes were described.

In the revised manuscript, we have clarified that the primary causal conclusion supported by our data is at the level of the upstream corticostriatal circuit: early hyperactivity in the mPFC→DMS pathway contributes to cognitive inflexibility in 5xFAD mice. By contrast, we do not claim that the dMSN→CIN→ACh pathway has been directly established here as an independently necessary or sufficient mediator of the behavioral phenotype.

Accordingly, we have moderated the causal language throughout the Abstract, Results, and Discussion. In particular, cholinergic alterations are now framed as downstream circuit consequences associated with aberrant corticostriatal activity, rather than as a directly demonstrated causal bottleneck. We have also revised the manuscript more broadly to improve interpretive precision and reporting clarity.

Detailed responses to the reviewer's concerns regarding statistical/reporting rigor and mechanistic specificity are provided in **Rev1_Con4** and **Rev1_Con6, 7 and 8**, respectively. We thank the reviewer again for raising this important point, which helped us sharpen the central claims of the study and better align the manuscript's wording with what is directly supported by the data.

Major comments:
Rev1_Con2

Figure 1

My main concern is interpretive precision. Figure 1 supports an early deficit in updating action-outcome contingencies, but the authors should be careful not to overstate this as a general executive dysfunction phenotype on the basis of one reversal paradigm alone. In addition, the main figure and legend/reporting should make it absolutely transparent how sample sizes were defined for each behavioral panel and whether sex was balanced across cohorts. Finally, because the entire paper builds on this behavioral dissociation, effect sizes and confidence intervals would be especially useful here.

Response: We thank the reviewer for this important comment regarding interpretive precision. We agree that Figure 1 supports an early deficit in updating action–outcome contingencies during instrumental reversal learning and should not be overstated as evidence for a generalized executive dysfunction phenotype on the basis of a single reversal paradigm alone.

In response, we revised the broader framing of the manuscript to use more specific language. In particular, wording that was previously framed more broadly in terms of executive dysfunction is now framed more specifically in terms of cognitive inflexibility, which more accurately reflects the behavioral evidence provided by the instrumental reversal-learning task. We also added the following limitation statement to the Discussion: "Although instrumental reversal learning is an important component of executive function, the present data do not establish a generalized executive dysfunction phenotype at this stage and are limited to impaired updating of action–outcome contingencies in this task." (Lines 458-461)

Regarding reporting transparency, the Figure 1 legend reports sample sizes as the number of mice for each behavioral comparison. With respect to sex as a biological variable, both male and female mice were included, and the revised Methods now state explicitly that sexes were balanced across experimental groups where possible. In addition, we examined sex as a factor in the baseline reversal-learning cohort and did not detect a significant sex-dependent effect (Reb-Fig. 1). These findings are consistent with previous reports showing minimal sex differences in behavioral and pathological phenotypes at early stages of 5xFAD mice (Oblak et al., 2021). Therefore, data from both sexes were pooled for analysis. This clarification has been added to the revised manuscript.

We agree that effect sizes and confidence intervals are useful for interpreting the behavioral dissociation in Figure 1. To improve transparency, detailed statistical information together with the underlying numerical values for all analyses is provided in the Supplementary Source Data file. We thank the reviewer for raising this point, which helped us improve the precision, transparency, and reporting clarity of the manuscript.

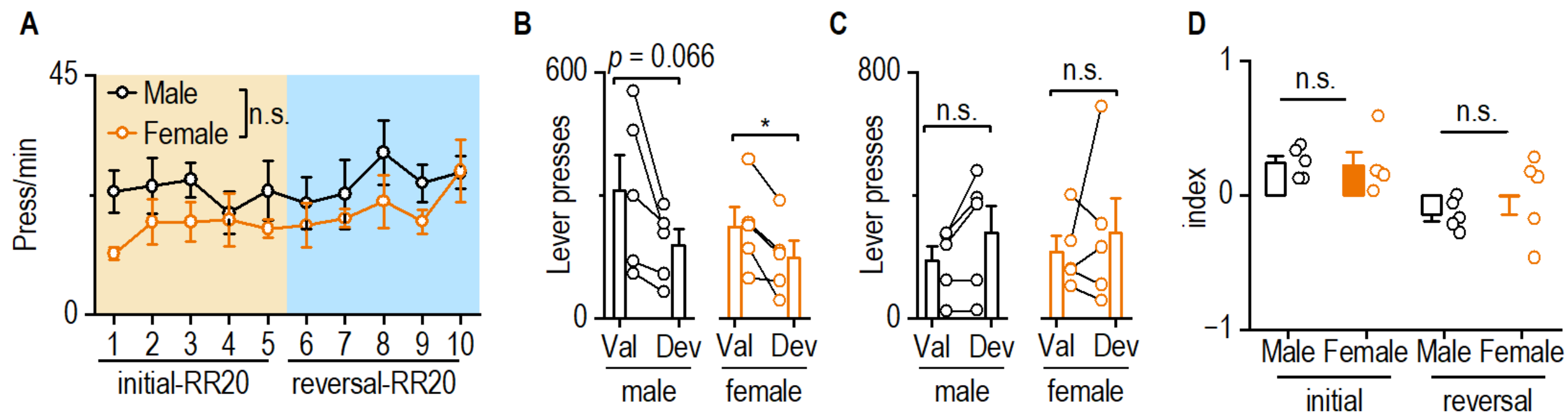

Reb-Figure 1. Sex does not influence instrumental learning or reversal performance in 5xFAD mice.

A, Lever-press rate across training. Male and female 5xFAD mice showed comparable response rates during both initial random-ratio 20 (RR20) training and reversal RR20 sessions (no significant sex effect). Two-way RM ANOVA, $F_{(1, 8)}$ = 2.026, p = 0.1925. n = 5 male and 5 female 5xFAD mice.

B, Outcome devaluation test following initial training. Both male and female mice made more responses on the valued lever than on the devalued lever, indicating acquisition of the initial action–outcome contingency. Pair t-test, t_4 = 2.508, p = 0.066 for male; t_4 = 3.659 p = 0.022 for female. n = 5 male and 5 female 5xFAD mice.

C, Outcome devaluation test following reversal training. Neither male nor female mice showed a preference for the valued over the devalued lever, indicating failure to update the action–outcome contingency after reversal. Pair t-test, t_4 = -2.273, p = 0.085 for male; Wilcoxon Signed Rank Test Z = 0.135 p = 1 for female. n = 5 male and 5 female 5xFAD mice.

D, Learning index during initial and reversal phases did not differ between sexes. Two-way RM ANOVA, $F_{(1, 8)}$ = 0.293, p = 0.603; Post-hoc analysis: comparisons for factor: sex within initial p = 0.888; Comparisons for factor: sex within reversal: p = 0.314. n = 5 male and 5 female 5xFAD mice.

Rev1_Con3

Figure 2

The ArcTRAP/c-Fos experiments are interesting and add an ensemble-level dimension to the story. The finding that reversal learning in 5xFAD mice is associated with increased activation/reactivation in mPFC and DMS is consistent with the circuit model proposed by the authors.

That said, this figure should be interpreted more cautiously. As the authors themselves later acknowledge, ArcTRAP integrates activity over relatively broad time windows and cannot resolve specific task epochs. Therefore, the data support abnormal recruitment/reactivation associated with reversal learning, but they do not prove that the same maladaptive ensemble dynamics are the mechanism of behavioral inflexibility. I would recommend softening the wording around "excessive reactivation" unless the authors can provide temporally sharper evidence. Clearer reporting of biological n at the animal level would also help.

Response: We thank the reviewer for this important point. We agree that the ArcTRAP/c-Fos experiments should be interpreted cautiously, as these data support abnormal recruitment/reactivation associated with reversal learning but do not by themselves establish that the same ensemble dynamics are the mechanism of behavioral inflexibility. To address this concern, we softened the wording around "excessive reactivation." In the revised manuscript, the Figure 2 title has been changed from **"Reversal learning in 5xFAD mice reactivates excessive mPFC and DMS neurons"** to **"Reversal learning in 5xFAD mice reactivates more mPFC and DMS neurons."** In addition, to improve the clarity of biological replication, we now state at the beginning of the Figure 2 legend that, for the quantification in panels C, E, H and I, each data point represents the mean value from 3–4 sections per mouse, and that biological n indicates the number of mice per group (Lines 1232-1233). We thank the reviewer for raising this point, which helped us improve the precision of the manuscript.

Rev1_Con4

Figure 3

This figure is convincing in showing that mPFC pyramidal neurons in young 5xFAD mice are hyperexcitable and receive increased excitatory input. The combination of intrinsic excitability, spontaneous EPSCs, evoked EPSCs, and AMPA/NMDA ratio gives a coherent picture of cortical hyperexcitability at an early stage.

The main issue here is statistical and inferential. For all electrophysiological analyses, the authors should clearly state whether statistics were performed at the neuron level or animal level, and ideally reanalyze key datasets using mixed-effects models that account for cells nested within animals. Without that, the strength of the conclusion is harder to judge. I also think the discussion should more clearly distinguish between evidence for increased excitatory transmission and the more speculative suggestion of impaired glutamate clearance/spillover.

Response: We thank the reviewer for this important and constructive comment. In the revised manuscript, we have clarified that electrophysiological statistical analyses were originally performed at the neuron level, and we now explicitly report both the number of recorded neurons and the number of animals in the Methods and corresponding figure legends (Methods, Lines 843–848; Figure legends, e.g., Lines 1243).

To directly address the reviewer's concern that neurons recorded from the same animal are not fully independent observations, we reanalyzed the key electrophysiological datasets using the recommended linear mixed-effects approach, which accounts for neurons nested within animals. Specifically, we focused on the principal datasets underlying our main conclusions, Fig. 3G, Fig. 4E, and Fig. 5D, and compared three analytical approaches: (1) linear mixed-effects models, (2) animal-level analysis using two-way repeated-measures ANOVA, and (3) neuron-level analysis, which was the original framework used in the manuscript.

We reanalyzed the data using a linear mixed-effects model with group, stimulus, and their interaction as fixed effects, and random effects to account for repeated measurements within neurons and neurons sampled within animals. Models were fitted using restricted maximum likelihood (REML), and fixed effects were evaluated using Type III ANOVA with Satterthwaite's approximation for degrees of freedom. As summarized in **Table 1**, all three analytical

approaches consistently identified a significant Group × Stimulus interaction across the key datasets. These results indicate that the main biological conclusion, that the two groups exhibit divergent response profiles as stimulus intensity increases, remains unchanged after accounting for the hierarchical structure of the data.

We agree that accounting for the hierarchical structure of the data is important. At the same time, in electrophysiological studies, the biological objective is often to characterize response properties at the level of individual neurons. In this setting, neuron-to-neuron variability is not simply technical noise, but a biologically meaningful feature of the system under study. For this reason, neuron-level analyses are often used in studies of single-cell physiology, provided that sampling across animals is appropriately controlled. Consistent with this practice, multiple animals were included in each experiment to ensure that neuronal sampling was representative across individuals (**Reb-Fig. 2**). In addition, neuron-level analyses continue to appear in recent electrophysiological studies, including reports published in *Nature Communications* (Liu et al., 2025; Luo et al., 2025; Stagkourakis et al., 2025). We therefore view neuron-level analysis as biologically informative in this context, while the added mixed-effects and animal-level analyses in this rebuttal letter further demonstrate that our conclusions are robust to the choice of statistical framework.

Finally, we have revised the Discussion to more clearly distinguish between results that directly support increased excitatory transmission and the more speculative interpretation regarding impaired glutamate clearance and/or spillover (Lines 420–427 and 471-475). We thank the reviewer again for this valuable suggestion, which has improved the clarity and rigor of the revised manuscript.

Table 1. Concordance of statistical results across analytical approaches for key electrophysiological datasets

Key dataset	mixed-effects model (neurons nested within animals)	2-way RM ANOVA (Animal-level analysis)	2-way RM ANOVA (Neuron-level analysis)
Fig. 3G<br>(eEPSCs in mPFC neurons)	Group:Stimulus<br>$F(5, 95) = 15.09$,<br>$P = 6.88 \times 10^{-11}$	stimulation x genotype<br>$F(5, 20) = 13.06$<br>$P < 0.0001$	stimulation x genotype<br>$F(1.590, 30.21) = 15.09$<br>$P < 0.0001$
Fig. 4E<br>(oEPSCs: mPFC-dMSNs)	Group:Stimulus<br>$F(10, 230) = 4.30$,<br>$P = 1.64 \times 10^{-5}$	stimulation x genotype<br>$F(10, 60) = 4.613$<br>$P < 0.0001$	stimulation x genotype<br>$F(1.639, 37.70) = 4.298$<br>$P = 0.0272$
Fig. 5D<br>(oIPSCs: dMSNs-CINs)	Group:Stimulus<br>$F(10, 450) = 5.52$,<br>$P = 9.17 \times 10^{-8}$	stimulation x genotype<br>$F(10, 40) = 5.927$<br>$P < 0.0001$	stimulation x genotype<br>$F(2.309, 103.9) = 42.51$<br>$P < 0.0001$
Comment	Reviewer suggested	Additional analysis	Currently used in the manuscript

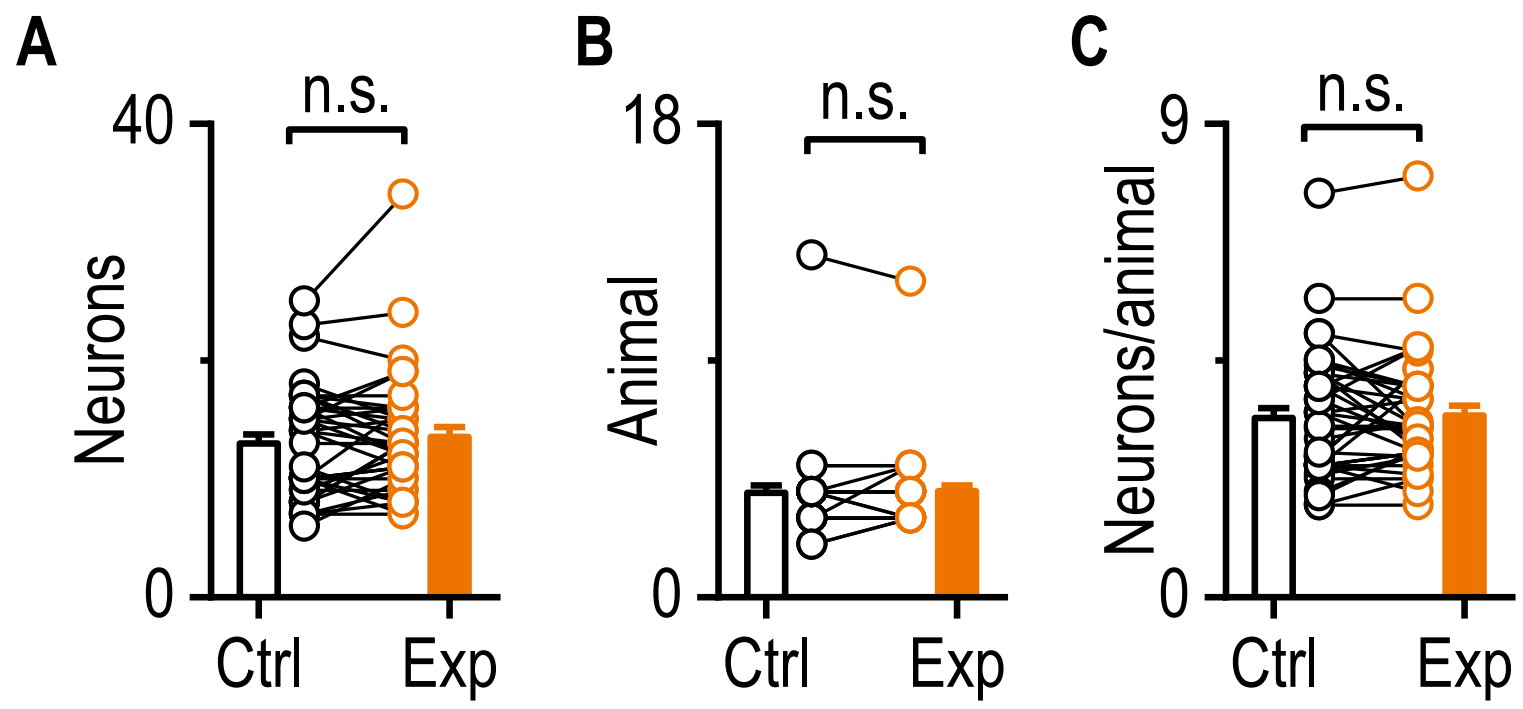

Reb-Figure 2. Comparable numbers of recorded neurons, animals, and neuron-to-animal ratios in control versus experimental groups.

A, Number of recorded neurons per graph panel in the control and experimental groups.
B, Number of animals used per graph panel in the control and experimental groups.
C, Neuron-to-animal ratio per graph panel, calculated by dividing the number of recorded neurons by the number of animals.

Each control–experimental pair corresponds to a single graph panel. In total, 38 pairs of electrophysiological and live-tissue confocal imaging experiments were included. No significant differences were observed between groups (paired t-test, $p > 0.05$). n.s., not significant.

Rev1_Con5
Figure 4

This is one of the most interesting figures in the paper. The selective enhancement of mPFC input onto dMSNs, but not iMSNs, provides real specificity and strengthens the mechanistic logic of the model. The persistence of this abnormality at 12 months is also notable.

Still, several points need clarification. First, the figure would benefit from more explicit reporting of viral targeting and exclusion criteria in the main text or figure legend. Second, the authors should be cautious with the language "selectively hyperactive," because what is shown directly is enhanced excitability/synaptic drive under the recording conditions, not necessarily in vivo firing selectivity during behavior. Third, the seizure and EEG data are useful as supportive evidence of broader network hyperexcitability, but they are relatively indirect with respect to the specific mPFC→dMSN pathway and should not be treated as pathway-specific confirmation.

Response: We thank the reviewer for this important point. We agree that the reporting of viral targeting and the interpretation of the Figure 4 data should be stated more precisely. In response, we revised the manuscript in several ways. First, we now state explicitly in the Results that AAV-Chronos-GFP was infused into the mPFC to enable optogenetic stimulation of mPFC terminals in the DMS while distinguishing dMSNs from iMSNs, and that viral expression was verified during recordings by confirming GFP signal at the injection site and labeled projections in the DMS (Lines 207-210). We also now state that slices were excluded if infection was insufficient or if DMS projections were weak or absent (Lines 207-209). Thus, the revised manuscript now provides explicit targeting-validation and exclusion criteria rather than relying only on the representative image in Fig. 4C.

We also revised the wording of this section to avoid overstating what is directly shown by the recordings. In the original version, the section title referred to the mPFC-to-dMSN circuit and dMSNs as “selectively hyperactive.” In the revised manuscript, this has been softened to “hyperactive,” and the concluding text now states more specifically that the recordings indicate enhanced excitatory drive onto dMSNs within the mPFC–DMS circuit.

We further agree that the seizure-susceptibility and EEG assays are indirect with respect to the specific mPFC→dMSN pathway. Our intention was not to use these in vivo measures as pathway-specific confirmation, but rather as supportive evidence of broader network hyperexcitability in 5xFAD mice. We recognize, however, that the prior wording and placement within the Figure 4 section may not have made this distinction sufficiently explicit. We therefore revised the text to more clearly separate the ex vivo circuit-specific findings from the seizure/EEG data and to state that the latter provide independent evidence of broader network-level hyperexcitability rather than pathway-specific confirmation (Lines 224-225 and 234-237). We thank the reviewer for raising these concerns, which helped us improve the clarity, precision, and interpretive restraint of the manuscript.

Rev1_Con6
Figure 5

This is the key mechanistic bridge in the manuscript. The evidence that dMSN-->CIN inhibition is enhanced, CIN spontaneous firing is reduced, and spontaneous ACh events are decreased is internally coherent and fits the proposed model well. This is an important strength of the paper.

However, this figure still stops short of demonstrating that reduced CIN/ACh signaling is the necessary mediator of the behavioral phenotype. The manuscript would be substantially strengthened by a direct rescue experiment at this node, for example restoring CIN activity or cholinergic tone and testing whether reversal learning improves. Without such an experiment, the safest conclusion is that altered dMSN→CIN signaling is a plausible and well-supported intermediate mechanism, not yet a definitively established causal bottleneck.

Response: We thank the reviewer for this important point. We agree that Figure 5 does not directly demonstrate that reduced CIN/ACh signaling is the necessary mediator of the behavioral phenotype. We also agree that a direct rescue experiment at this node, such as restoring CIN activity or cholinergic tone during reversal learning, would provide a stronger test of necessity and would further strengthen the study.

At the same time, we believe that Figure 5 provides an important mechanistic bridge linking corticostriatal hyperactivity to downstream cholinergic dysfunction. Specifically, our data show that in 5xFAD mice: (1) dMSN-to-CIN inhibitory transmission is enhanced, (2) both presynaptic and postsynaptic components of inhibitory drive onto CINs are increased, (3) spontaneous firing of CINs is reduced while evoked firing remains intact, (4) spontaneous striatal ACh release is decreased, and (5) CIN-to-MSN inhibition is also reduced. Together, these convergent findings support altered dMSN-to-CIN signaling as a plausible and well-supported intermediate mechanism linking corticostriatal hyperactivity to reduced striatal cholinergic output. However, we agree that these data do not yet establish this node as a definitively necessary causal bottleneck for the behavioral deficit.

To address this concern, we revised the manuscript to reduce the specificity of our mechanistic claims, while preserving the mechanistic significance of the Figure 5 findings. In the Figure 5 Results section, we retained cautious wording in the interpretation of the CIN physiology data, stating that the reduced spontaneous firing is "consistent with CIN hypoactivity arising from increased inhibitory input rather than changes in intrinsic excitability," and concluding that the findings indicate "coordinated alterations in dMSN–CIN interactions and cholinergic signaling in 5xFAD mice, consistent with downstream circuit changes associated with corticostriatal hyperactivity." Additionally, we remove the sentence that "This disruption in cholinergic signaling may contribute to the early cognitive inflexibility observed in AD." Thus, the Figure 5 text now emphasizes a mechanistically coherent intermediate pathway rather than a directly established behavioral bottleneck.

We also revised the broader framing in the Abstract, Results, and Discussion to ensure that the downstream CIN/ACh node is not presented with stronger causal certainty than the data support. In the Abstract, enhanced corticostriatal transmission is described as being **associated with** strengthened inhibition of CINs, reduced spontaneous firing, and diminished striatal ACh release, while the intervention is described as reducing cortical amyloid accumulation, decreasing glutamatergic transmission, increasing ACh levels, and rescuing reversal learning, without claiming that cholinergic restoration itself is proven to mediate the behavioral rescue. In the later Results, these intervention effects are described as concurrent outcomes of circuit inhibition. In the Discussion, we now explicitly state that, although mPFC-to-DMS circuit inhibition restores both cholinergic signaling and behavioral performance, the relative contribution of striatal cholinergic mechanisms to this rescue remains to be determined.

Accordingly, rather than claiming that Figure 5 establishes a necessary causal bottleneck, the revised manuscript presents altered dMSN→CIN→ACh signaling as a strong, biologically coherent, and well-supported intermediate mechanism within the broader corticostriatal model. A direct cell-type-specific rescue of CIN/ACh signaling during reversal learning is an important direction for future work, but is beyond the scope of the current revision. This revised framing is also consistent with our responses to Rev1_Con7 and Rev1_Con8, where we similarly reduced mechanistic specificity and clarified the distinction between upstream circuit causality and downstream mechanistic inference.

We thank the reviewer for raising this important issue, which helped us improve the precision, balance, and interpretive restraint of the manuscript. These comments prompted us to more clearly distinguish between a well-supported intermediate mechanism and a directly established necessary causal node.

Rev1_Con7

Figure 6

The long-term chemogenetic inhibition experiment is important because it adds an intervention dimension and links circuit activity to amyloid burden, synaptic transmission, and striatal ACh output. The result that projection-specific mPFC→DMS inhibition reduces cortical plaque burden and normalizes downstream readouts is potentially very significant.

At the same time, this figure also illustrates the manuscript's main interpretive problem: the intervention changes many variables at once. Because chronic hM4Di/C21 manipulation affects Aβ, cortical transmission, striatal transmission, and ACh, it is difficult to assign behavioral rescue specifically to cholinergic normalization. The authors should therefore reduce the specificity of the mechanistic claims, unless they add a more targeted experiment downstream. This section also needs especially careful reporting of viral spread, projection specificity, treatment controls, and how long after the last C21 injection the measurements were made.

Response: We thank the reviewer for this important point. We agree that chronic chemogenetic inhibition of mPFC-to-DMS projection neurons alters multiple downstream features in parallel, including cortical Aβ burden, cortical and striatal glutamatergic transmission, striatal cholinergic signaling, and behavioral performance. Therefore, our current data do not allow us to assign the rescue of reversal learning to any single downstream mechanism.

Our interest in striatal cholinergic signaling was based on **substantial** prior literature showing that DMS cholinergic interneurons (CINs) and their associated acetylcholine (ACh) signaling are critical components of this **exact** reversal-learning paradigm. Bradfield et al. (2013) showed that reducing cholinergic signaling in the DMS selectively impaired learning after action–outcome contingency reversal while sparing initial acquisition. Matamales et al. (2016) further showed that selective depletion of DMS CINs in young mice reproduced the aged phenotype, namely impaired reversal learning with preserved initial learning, similar to the pattern we observed in our Figure 1. Ma et al. (2022) showed that enhancing DMS CIN-related function rescued reversal-learning deficits in another disease model. More recently, Gangal et al. (2023) showed that chemogenetic or time-locked optogenetic inhibition of DMS CINs impaired reversal learning, and Huang et al. (2024) showed that disrupting CIN pause dynamics during reversal training impaired acquisition of the reversed contingencies. Together, this prior literature supports the idea that DMS CIN/ACh signaling is a strong candidate contributor of behavioral flexibility in this exact task.

At the same time, we agree with the reviewer that the present study does not directly demonstrate that restoration of striatal cholinergic signaling is the mechanism responsible for the behavioral rescue produced by chronic mPFC-to-DMS inhibition. To address this concern, we **revised the manuscript to reduce** the specificity of our mechanistic interpretation. In the revised text, the effects of sustained mPFC-to-DMS inhibition on cortical Aβ accumulation, corticostriatal transmission, striatal ACh signaling, and reversal learning are described as concurrent outcomes of circuit inhibition, rather than as a directly established causal sequence (Abstract, Lines 21-25; Results, Lines 324–326; Discussion, Lines 366–376 and 408–410). We also revised the schematic legend and accompanying text to emphasize these as concurrent circuit and behavioral outcomes of the intervention (Figure 7G,H). More specifically, Fig. 7G is intended as a literature-informed hypothetical model that integrates our findings with prior work implicating DMS cholinergic signaling in reversal learning, whereas Fig. 7H is intended as the more conservative summary of what is directly supported by our intervention data, namely concurrent improvement in circuit, cholinergic, and behavioral readouts following sustained mPFC-to-DMS inhibition. In addition, we now state explicitly in the Discussion that, although cholinergic signaling and behavioral performance improve in parallel, the relative contribution of striatal cholinergic restoration to the behavioral rescue remains unresolved (Discussion, Lines 407–410).

“viral spread, projection specificity”

We thank the reviewer for highlighting the need for more careful reporting of viral spread and projection specificity. In response, we **added new histological data** documenting the location and spread of hM4Di-mCherry expression across representative coronal sections of the mPFC, together with quantitative measurements of expression extent (**Reb-Fig. 3** and **Table 2** below; Supplementary Fig. 11). These data provide direct validation of the anatomical distribution of viral expression in the cortical region. We also now clarify the projection-defined dual-virus design in the revised manuscript: retrograde Cre was delivered to the DMS, while Cre-dependent AAV-DIO-hM4Di-mCherry or AAV-DIO-mCherry was delivered to the mPFC, thereby restricting expression to DMS-projecting mPFC neurons (Results, Lines 296–300).

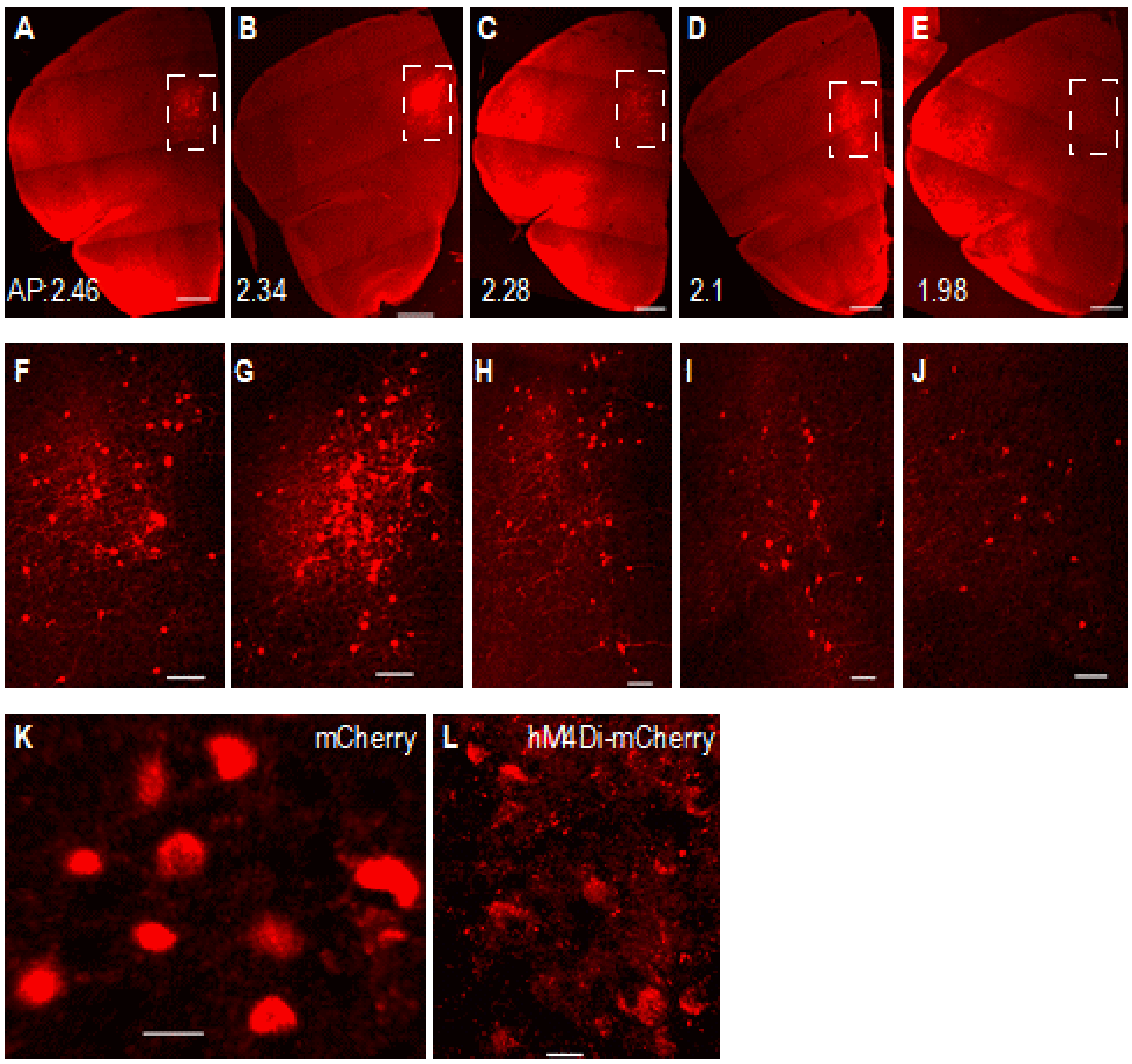

Reb-Figure 3. Confocal images showing the location and spread of hM4Di-mCherry expression (Same as Supplementary Figure 11)

A–E, Coronal sections of the mPFC displaying mCherry expression from bregma AP 2.46 to 1.98 mm. Scale bar: 500 μm.

F–J, Enlarged views of the dashed boxes in A–E, showing mCherry-positive neurons. Scale bar: 100 μm.

K, L, High-magnification images comparing mCherry expression in animals infused with AAV-DIO-mCherry (K) and AAV-DIO-hM4Di-mCherry (L). Scale bar: 20 μm. Note: Panels A–J were obtained from a mouse infused with AAV-DIO-mCherry.

Table 2. Summary of mCherry-expressing areas, diameters, and infected neurons

AP (mm)[$]	Area (μm²)	Diameter (μm)	Neuron infected[&]
2.46	1.06×10^6	1158	30
2.34	1.09×10^6	1178	45
2.28	1.36×10^6	1330	40
2.1	1.05×10^6	1156	17
1.98	9.22×10^5	1083	12
Mean ± SE	$1.11 \times 10^6 \pm 7.19 \times 10^4$	1190 ± 40.6	

[$]Total rostrocaudal distance sampled: 480 μm; [&]total neurons counted: 144.

"treatment controls"

We thank the reviewer for this important point. We revised the manuscript text (Results, Lines 296–305) and Fig. 6A (**Reb-Fig. 4A** below, for reviewer convenience) to clarify the treatment-control design by explicitly labeling the control virus as AAV-DIO-mCherry. Specifically, 5xFAD mice received either AAV-DIO-hM4Di-mCherry or the control virus AAV-DIO-mCherry. After ~6 weeks of viral expression, both groups received the same daily C21 treatment for 30 days. Therefore, the appropriate control in this experiment was the C21-treated mCherry-expressing group. This design controls for viral surgery, repeated injections, and C21 exposure, allowing the observed group differences to be attributed primarily to hM4Di-mediated chemogenetic inhibition.

"how long after the last C21 injection the measurements were made"

We thank the reviewer for this important point. We revised the manuscript text (Results, Lines 300–305 and 332–335; Methods, Lines 670–674) and Figs. 6A and 7A to clarify the timing of C21 administration relative to behavioral testing and post-treatment measurements. During the 30-day instrumental-task phase, mice first completed the daily instrumental session, and C21 was administered at least 6 h later (Fig. 7A, or **Reb-Fig. 4B** for reviewer convenience). This timing was used to minimize a potential effect of chemogenetic inhibition on memory consolidation shortly after each behavioral session (Nader and Hardt, 2009; Barak et al., 2013). After completion of the 30-day C21 treatment, post-treatment measurements were performed on a defined timeline (Fig. 6A, or **Reb-Fig. 4A** for reviewer convenience). The NOR procedure began 24 h after the last C21 injection, and the final NOR test occurred approximately 72 h after the last injection. Electrophysiological recordings, striatal ACh measurements, and histological analyses were then performed after completion of behavioral testing, within one week of the final injection. These revisions are now stated explicitly in the manuscript text and clarified in the revised figures.

We thank the reviewer for raising these critical questions, which have helped us improve the clarity, precision, and overall rigor of the manuscript. These comments were particularly valuable in prompting us to refine our mechanistic interpretation so that the conclusions more accurately reflect the evidence provided by the current data.

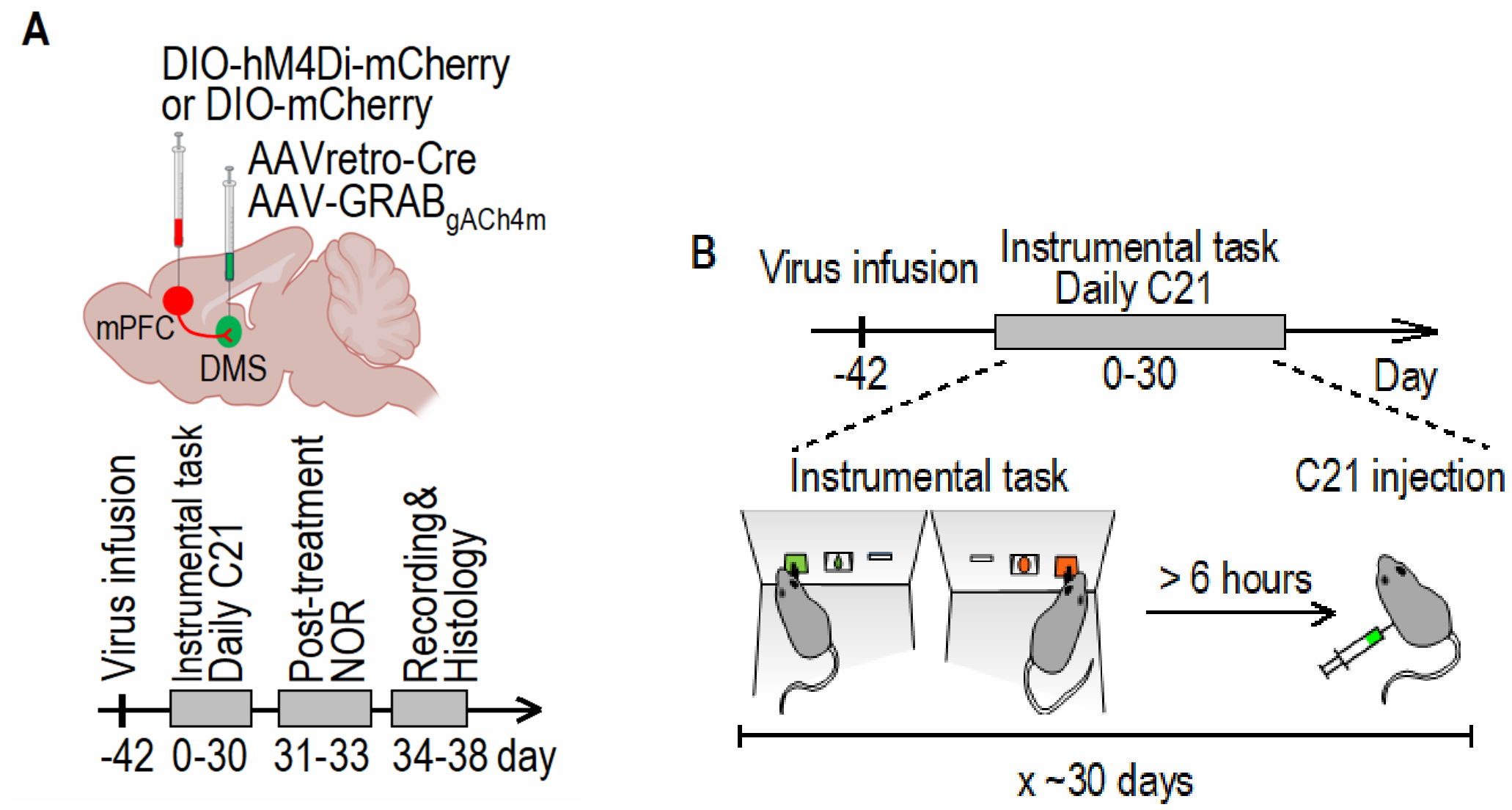

Reb-Figure 4. Experimental design and timing.

A. Schematic illustrating the viral infusion strategy (top) and overall experimental timeline (bottom) (same as **Figure 6A**).

B. Expanded view of the 0–30 day instrumental training / daily C21 phase shown in Fig. 6A. Mice underwent daily instrumental sessions for ~30 days, and C21 was administered at least 6 h after each session (same as **Figure 7A**).

Rev1_Con8
Figure 7

The behavioral rescue is compelling and gives the paper translational relevance. The fact that sustained mPFC→DMS inhibition restores reversal-learning performance in 5xFAD mice is an important result and fits well with the overall circuit model. The additional object-recognition improvement is also interesting.

My concern is again one of mechanistic specificity. Because the same chronic manipulation also reduced cortical Aβ accumulation and altered excitatory transmission, Figure 7 cannot by itself show that reversal-learning rescue occurred specifically because cholinergic signaling was restored. The figure therefore supports a strong causal role for the upstream circuit, but not yet the full downstream pathway claimed in the manuscript. This distinction should be reflected in the wording of the Results and Discussion.

Response: We thank the reviewer for this important point. We agree that Figure 7 supports a strong causal role for the upstream mPFC-to-DMS circuit in the behavioral rescue, but does not by itself establish that reversal-learning rescue occurred specifically because striatal cholinergic signaling was restored. As the reviewer noted, the same chronic manipulation also reduced cortical Aβ accumulation and altered excitatory transmission. Therefore, based on Figure 7 alone, the behavioral rescue cannot be assigned to any single downstream mechanism.

This concern substantially overlaps with the broader issue of mechanistic specificity raised in Rev1_Con7, and we have revised the manuscript accordingly. In particular, we reduced the specificity of the mechanistic interpretation in the Results and Discussion so that the effects of sustained mPFC-to-DMS inhibition on cortical Aβ accumulation, corticostriatal transmission, striatal ACh signaling, and reversal-learning performance are now described as concurrent outcomes of circuit inhibition, rather than as a directly established causal sequence (Abstract, Lines 23–25; Results, Lines 324–326 and 329–331; Discussion, Lines 366–376 and 408–410). Thus, while the revised manuscript continues to support a strong role for the mPFC-to-DMS circuit in the behavioral phenotype, it no longer overstates the downstream mechanistic conclusion.

We also revised the wording associated with Figure 7 to better reflect this distinction. Specifically, Figure 7G is intended as a literature-informed hypothetical model that integrates our findings with prior work implicating DMS cholinergic signaling in reversal learning (Bradfield et al., 2013; Matamales et al., 2016; Ma et al., 2022; Gangal et al., 2023; Huang et al., 2024), whereas Figure 7H is intended as the more conservative summary of what is directly supported by our intervention data, namely concurrent improvement in circuit, cholinergic, and behavioral readouts following sustained mPFC-to-DMS inhibition. Consistent with this, the revised figure legend now emphasizes concurrent circuit and behavioral outcomes of the intervention, rather than a directly established cholinergic causal chain.

In addition, we now state explicitly in the Discussion that, although cholinergic signaling and behavioral performance improve in parallel, the relative contribution of striatal cholinergic restoration to the behavioral rescue remains unresolved (Discussion, Lines 408–410). We believe these revisions now align the wording of the Results, Discussion, and Figure 7 more closely with what is directly supported by the current data.

For the reviewer's convenience, please also see our response to Rev1_Con7, where we address the related concern that chronic mPFC-to-DMS inhibition alters multiple downstream variables in parallel. We thank the reviewer for raising this important concern, which helped us improve the clarity, precision, and interpretive restraint of the manuscript. These comments were particularly valuable in prompting us to refine the wording so that our conclusions more accurately reflect what is directly supported by the present data.

Rev1_Con9

Summary of the overall concern

This is a strong and potentially impactful manuscript with a compelling central idea and a rich dataset. The behavioral dissociation is interesting, the pathway specificity in Figure 4 is a major strength, and the CIN/ACh data are mechanistically suggestive and important. However, the current version overstates the level of causal proof, and the statistical/reporting framework needs to be tightened substantially. With major revision, and ideally with a direct test of the CIN/ACh node, this study could become suitable for publication.

Response: We thank the reviewer for this thoughtful overall assessment. The concerns summarized here overlap substantially with issues addressed in detail in our responses to

Rev1_Con1 and Rev1_Con4–8. For clarity, we summarize below how these overarching points were addressed in the revised manuscript.

Table 3. Summary of revisions addressing the overarching concerns raised in Rev1_Con9

Overall concern	Revision made	See detailed response
Causal claims were overstated	Softened causal language	Rev1_Con1
CIN/ACh node not directly proven	Reframed as intermediate mechanism	Rev1_Con6
Rescue not attributable to one pathway	Described as concurrent outcomes	Rev1_Con7, Rev1_Con8
Statistical/reporting framework needed strengthening	Added mixed-effects reanalysis and clearer reporting	Rev1_Con4
Interpretive precision needed improvement	Revised wording throughout	Rev1_Con1, Rev1_Con4, Rev1_Con6, 7, 8

Rev2
Reviewer #2 (Remarks to the Author):
The authors have adequately addressed the points raised in my previous revision. The manuscript has improved accordingly and I have no further comments or concerns.

Response: We thank the reviewer for the thoughtful evaluation. We are pleased that the revised manuscript has addressed the previous concerns.

Rev3
Reviewer #3 (Remarks to the Author):
The authors have satisfactorily addressed the 10 points raised by my co-reviewer and myself. We particularly appreciated the additional behavioral experiments in which (i) the initial RR20 procedure was immediately followed by a reversed RR20 task without a preceding devaluation session, definitively ruling out motivational confounds(Rev3Maj1) and (ii) showing that 3xFAD mice perform well in a reversal test run in a radial maze, demonstrating that their deficit in cognitive flexibility is selective to the goal-directed/instrumental domain (Rve3Maj2).

The following issues were also satisfactorily addressed:
Rev3Maj3 (limiting the behavioral deficit to the PL/IL circuit rather than the mPFC as a whole), Rev3Maj6 (detailing the timing of isoflurane exposure relative to behavioral testing), Rev3Maj7

(discussing extensively why off-target (AchR)effects of compound 21 injections are unlikely), Rev3Maj8 (considering the potential involvement of hippocampal neurogenesis in cognitive flexibility), RevMaj9 (using two-way RM ANOVAs with genotype and session as main factors to validate inter-strain behavioral differences through significant genotype x session interactions), Rev4Maj10 (specifying the cognitive flexibility deficit of 5XFAD mice at the light of the Pierce-Messick et al., 2025 distinction between a deficit in updating previously learned action-outcome associations and a primary impairment in goal-directed control.

The "Supplementary Data" section, which includes more experiments than the "Main Data" section, has been left unchanged but the authors give justifications for that (Rev3Maj4). Also, the fact that behavioral and neurobiological investigations conducted at different ages partially support causal relationships has been taken into account (Rev3Maj5).

With regard to our area of expertise, we consider the manuscript suitable for publication.

Response: We thank the reviewer for the careful evaluation and are pleased that the revisions have satisfactorily addressed the concerns and improved the manuscript.

Rev4

Reviewer #4 (Remarks to the Author):

I appreciate the authors' acknowledgment of the molecular dimension in their revised discussion. However, the added sentence remains largely generic and does not substantively address the concern raised. For a study of this scope published in Nature Communications, I would expect the authors to leverage their own circuit-level findings as a foundation for specific molecular hypotheses, even in the absence of direct experimental evidence.

For instance, the documented mPFC hyperexcitability could invite speculation regarding reduced voltage-gated potassium channel expression or functon (e.g., Kv4.2 or Kv1 subfamily members), or impaired after hyperpolarization conductances — all of which have precedent in amyloid-driven neuronal dysfunction. Similarly, the enhanced glutamatergic corticostriatal transmission they report could be linked to known amyloid-associated mechanisms such as AMPA receptor subunit upregulation, reduced astrocytic glutamate clearance via GLT-1/EAAT2, or increased presynaptic release probability. Finally, the suppression of striatal cholinergic interneuron firing could be discussed in the context of M2 receptor dysregulation or nicotinic receptor desensitization, both of which are plausible downstream consequences of the circuit pathology described.

Response: We thank the reviewer for this valuable and insightful comment. In response, we substantially expanded the Discussion to include more specific, hypothesis-driven molecular interpretations grounded in our circuit-level findings. Specifically, we now discuss potential contributors to mPFC hyperexcitability, including impaired Kv4.2/Kv1-mediated repolarizing currents and reduced adaptation currents; mechanisms that may enhance corticostriatal glutamatergic transmission, including impaired astrocytic glutamate clearance, increased presynaptic release probability, and altered postsynaptic AMPA receptor signaling; and additional cholinergic mechanisms that could exacerbate CIN hypoactivity, including muscarinic autoreceptor dysregulation and nicotinic receptor dysfunction. These additions are now

incorporated into the revised Discussion (lines 462–489). We thank the reviewer for this suggestion, which helped strengthen the mechanistic depth and clarity of the manuscript.

Rev5

Reviewer #5 (Remarks to the Author):

I co-reviewed this manuscript with one of the reviewers who provided the listed reports. This is part of the Nature Communications initiative to facilitate training in peer review and to provide appropriate recognition for Early Career Researchers who co-review manuscripts.

Response: We thank the reviewer for their evaluation and for the constructive comments provided in the reports.

References

Barak S, Liu F, Ben Hamida S, Yowell QV, Neasta J, Kharazia V, Janak PH, Ron D (2013) Disruption of alcohol-related memories by mTORC1 inhibition prevents relapse. Nature Neuroscience 16:1111–U1185.

Bradfield LA, Bertran-Gonzalez J, Chieng B, Balleine BW (2013) The thalamostriatal pathway and cholinergic control of goal-directed action: interlacing new with existing learning in the striatum. Neuron 79:153–166.

Gangal H, Xie X, Huang Z, Cheng Y, Wang X, Lu J, Zhuang X, Essoh A, Huang Y, Chen R, Smith LN, Smith RJ, Wang J (2023) Drug reinforcement impairs cognitive flexibility by inhibiting striatal cholinergic neurons. Nat Commun 14:3886.

Huang Z, Chen R, Ho M, Xie X, Gangal H, Wang X, Wang J (2024) Dynamic responses of striatal cholinergic interneurons control behavioral flexibility. Sci Adv 10:eadn2446.

Liu Y et al. (2025) Prolonged exposure to food odors suppresses feeding via an olfactory bulb-to-hypothalamus circuit. Nat Commun 16:7892.

Luo Z, Chen J, Liu Y, Dai Y, Gao H, Zhang B, Ou H, So KF, Wei JA, Zhang L (2025) Treadmill exercise prevents stress-induced anxiety-like behaviors via enhancing the excitatory input from the primary motor cortex to the thalamocortical circuit. Nat Commun 16:939.

Ma T, Huang Z, Xie X, Cheng Y, Zhuang X, Childs MJ, Gangal H, Wang X, Smith LN, Smith RJ, Zhou Y, Wang J (2022) Chronic alcohol drinking persistently suppresses thalamostriatal excitation of cholinergic neurons to impair cognitive flexibility. J Clin Invest 132:e154969.

Matamales M, Skrbis Z, Hatch RJ, Balleine BW, Gotz J, Bertran-Gonzalez J (2016) Aging-Related Dysfunction of Striatal Cholinergic Interneurons Produces Conflict in Action Selection. Neuron 90:362–373.

Nader K, Hardt O (2009) A single standard for memory: the case for reconsolidation. Nature Reviews Neuroscience 10:224–234.

Oblak AL et al. (2021) Comprehensive Evaluation of the 5XFAD Mouse Model for Preclinical Testing Applications: A MODEL-AD Study. Front Aging Neurosci 13:713726.

Stagkourakis S, Williams P, Spigolon G, Khanal S, Ziegler K, Heikkinen L, Fisone G, Broberger C (2025) Maternal aggression driven by the transient mobilisation of a dormant hormone-sensitive circuit. Nat Commun 16:8553.

Point-by-point response to reviewers' concerns

Reviewer #1 (Remarks to the Author):

The authors have substantially improved the manuscript. The revised version addresses the main conceptual concern by framing the strongest causal conclusion at the level of upstream mPFC→DMS corticostriatal hyperactivity, while treating dMSN-->CIN-->ACh changes as associated downstream mechanisms rather than as a proven necessary/sufficient behavioral bottleneck. This is reflected in the Abstract, where cholinergic changes are described as coincident with enhanced corticostriatal transmission and the behavioral rescue is attributed to sustained corticostriatal inhibition rather than to direct CIN/ACh rescue alone.

The behavioral framing is also improved. The authors now focus the claim on impaired instrumental reversal learning/action–outcome updating, and the merged manuscript appropriately distinguishes this from broader spatial learning deficits, which are absent at 3 months and emerge later. The added clarification that the data do not establish a generalized executive dysfunction phenotype is appropriate and important.

The statistical response is mostly satisfactory. The authors performed the requested mixed-effects reanalysis for key electrophysiological datasets, and the reported results support the robustness of the main conclusions after accounting for cells nested within animals. The manuscript now states that behavioral and histology data are analyzed at the animal level, live imaging at the slice level, and electrophysiology at the neuron level with animal numbers provided in legends/source data. This substantially improves transparency.

The revisions to Figure 4 and the seizure/EEG interpretation are also acceptable. The revised manuscript now states viral targeting/exclusion criteria for mPFC terminal stimulation in DMS and clearly separates ex vivo pathway-specific evidence from seizure/EEG evidence of broader network hyperexcitability.

I do not think additional experiments are required at this stage. In particular, I would no longer insist on a CIN/ACh rescue experiment, provided the authors preserve the current more cautious interpretation. However, I recommend minor textual revision before acceptance to remove a few residual overstatements:

In the Abstract, "pathological corticostriatal hyperactivity acts as a circuit mechanism underlying early cognitive inflexibility in Alzheimer's disease" should be softened to "contributes to early cognitive inflexibility in a mouse model of Alzheimer's disease" or similar. The upstream circuit manipulation supports causality in 5xFAD mice, but the current wording remains slightly broad for human AD.

In the Discussion, the sentence stating that CIN hypoactivity "results from excessive GABAergic inhibition" and that this cascade "compromises striatal output and contributes to behavioral rigidity" should be softened to "is consistent with" and "may contribute," because the relative contribution of striatal cholinergic restoration to behavioral rescue is explicitly unresolved.

The phrase "excessive reactivation" still appears in the Discussion, despite the authors' response that this wording was softened. I recommend changing it to "increased reactivation" or "greater reactivation," consistent with the revised Figure 2 title.

The mixed-effects reanalysis should be included in the manuscript or supplement/source data, not only in the rebuttal, because it directly addresses the independence concern for the electrophysiology datasets.

Response: We sincerely thank the reviewer for the thoughtful comments and positive evaluation of our revised manuscript. We are grateful that the reviewer found the revisions satisfactory and that no additional experiments are required at this stage.

We have addressed the remaining textual overstatements noted by the reviewer as follows:

- **Abstract:** We softened the final sentence to avoid an overly broad causal statement regarding human Alzheimer's disease and revised the wording to indicate that corticostriatal hyperactivity "contributes to early cognitive inflexibility in a mouse model of Alzheimer's disease" (Line 36).
- **Discussion:** We revised the language describing CIN hypoactivity and downstream circuit effects to use more cautious wording, including "is consistent with" and "may contribute," to reflect that the relative contribution of striatal cholinergic restoration to behavioral rescue remains unresolved (Line 415).
- **Discussion wording:** We changed "excessive reactivation" to "greater reactivation," consistent with the revised Figure 2 title (Line 428).
- **Mixed-effects reanalysis:** We have included the detailed statistical information for the key mixed-effects reanalyses, previously presented in the rebuttal, in the source data file to address the reviewer's concern regarding statistical independence in the electrophysiological datasets.

We thank the reviewer again for their thoughtful suggestions, which have helped improve the clarity, rigor, and overall quality of the manuscript.
